# Peripheral blood epi-signature of Claes-Jensen syndrome enables sensitive and specific identification of patients and healthy carriers with pathogenic mutations in *KDM5C*

**DOI:** 10.1186/s13148-018-0453-8

**Published:** 2018-02-14

**Authors:** Laila C. Schenkel, Erfan Aref-Eshghi, Cindy Skinner, Peter Ainsworth, Hanxin Lin, Guillaume Paré, David I. Rodenhiser, Charles Schwartz, Bekim Sadikovic

**Affiliations:** 1 Department of Pathology and Laboratory Medicine, Western University, London, Ontario Canada; 2 Molecular Genetics Laboratory, Molecular Diagnostics Division, London Health Sciences Centre, London, Ontario Canada; 3 Greenwood Genetics Center, Greenwood, SC USA; 4 Department of Pathology and Molecular Medicine, McMaster University, Hamilton, Ontario Canada; 5 Departments of Pediatrics, Biochemistry and Oncology, Western University, London, Ontario Canada; 6 Department of Pathology and Laboratory Medicine, Victoria Hospital, London Health Sciences Centre, 800 Commissioner’s Road E, B10-104, London, Ontario N6A 5W9 Canada

**Keywords:** KDM5C, DNA methylation, Variants of unknown significance, X-linked intellectual disability, Claes-Jensen

## Abstract

**Background:**

Claes-Jensen syndrome is an X-linked inherited intellectual disability caused by mutations in the *KDM5C* gene. Kdm5c is a histone lysine demethylase involved in histone modifications and chromatin remodeling. Males with hemizygous mutations in *KDM5C* present with intellectual disability and facial dysmorphism, while most heterozygous female carriers are asymptomatic. We hypothesized that loss of Kdm5c function may influence other components of the epigenomic machinery including DNA methylation in affected patients.

**Results:**

Genome-wide DNA methylation analysis of 7 male patients affected with Claes-Jensen syndrome and 56 age- and sex-matched controls identified a specific DNA methylation defect (epi-signature) in the peripheral blood of these patients, including 1769 individual CpGs and 9 genomic regions. Six healthy female carriers showed less pronounced but distinctive changes in the same regions enabling their differentiation from both patients and controls. Highly specific computational model using the most significant methylation changes demonstrated 100% accuracy in differentiating patients, carriers, and controls in the training cohort, which was confirmed on a separate cohort of patients and carriers. The 100% specificity of this unique epi-signature was further confirmed on additional 500 unaffected controls and 600 patients with intellectual disability and developmental delay, including other patient cohorts with previously described epi-signatures.

**Conclusion:**

Peripheral blood epi-signature in Claes-Jensen syndrome can be used for molecular diagnosis and carrier identification and assist with interpretation of genetic variants of unknown clinical significance in the *KDM5C* gene.

**Electronic supplementary material:**

The online version of this article (10.1186/s13148-018-0453-8) contains supplementary material, which is available to authorized users.

## Background

DNA methylation and histone modifications are the most widely studied epigenetic mechanisms, involved in the control of gene expression and maintenance of genomic stability. Post-translational modifications of histone proteins are coordinated by a variety of enzymes that catalyze histone methylation, demethylation, acetylation, deacetylation, phosphorylation, ubiquitination, SUMOylation, and ADP-ribosylation [1]. Mutations in the genes involved in the modification of histones cause a broad spectrum of Mendelian disorders [2]. Among these are mutations in the X-linked gene *KDM5C*, which encodes the histone H3 lysine 4 (H3K4) demethylase protein and causes Claes-Jensen syndrome [2].

The clinical manifestations in affected males carrying hemizygous *KDM5C* mutations include intellectual disability, impairments in adaptive behavior, slowly progressive spastic paraplegia, seizures, and facial dysmorphism [3, 4]. *KDM5C* is among the genes that escape X-chromosome inactivation; therefore, female mutation carriers usually remain unaffected but can demonstrate mild learning deficits [5].

Recent studies have suggested a bidirectional relationship between DNA methylation and histone modification which regulates locus-specific gene activity [6]. For instance, the DNA methyltransferase Dnmt3b1 selectively binds to the bodies of transcribed genes, leading to their de novo methylation, and its recruitment requires co-transcriptional deposition of histone H3 trimethylation at lysine 36 [7]. These findings have encouraged global efforts to elucidate the specific underlying molecular mechanisms that may be altered in epigenetic syndromes and to identify epi-signatures that can be used for diagnosis, particularly in those patients whose clinical manifestations are associated with a phenotypic spectrum shared across more than one syndrome, a situation where a specific clinical diagnosis is difficult to make.

Using a genome-wide DNA methylation analysis, we and others have identified specific DNA methylation epi-signatures in the peripheral blood of patients with a number of genetic diseases that result from the disruption of epigenomic machinery, including alpha-thalassemia/mental retardation X-linked (ATRX) syndrome [8], Floating-Harbor syndrome [9], DNA methyltransferase 1 (DNMT1)-associated autosomal dominant cerebellar ataxia, deafness, and narcolepsy syndrome [10], along with Kabuki [11, 12] and Sotos syndromes [13]. These epi-signatures from peripheral blood specimens can be used to accurately identify patients with these conditions, to assist with the interpretation of genetic variants of unknown significance in related genes, and ultimately, to be used as part of the routine molecular screening protocols in patients with a broad range of developmental delay and intellectual disability disorders [14, 15].

In this report, we used a high-resolution genome-wide methylation microarray analysis to describe a specific methylation profile in peripheral blood samples of a cohort of affected males with loss-of-function *KDM5C* mutations and a similar but less pronounced methylation change in healthy female carriers. We demonstrate that the DNA methylation signature of Claes-Jensen syndrome has the potential to be used as a diagnostic tool for detection of both patients and healthy carriers with *KDM5C* mutations. We further show that this epi-signature is highly specific to *KDM5C* mutations, but not to the broad range of other Mendelian diseases resulting from the disruption of genes in the epigenomic machinery or other forms of developmental delay and intellectual disabilities (DD/ID).

## Methods

### Study cohort

Peripheral blood samples from patients referred for genetic testing at the Greenwood Genetic Center were collected for methylation study. All patients were screened for mutations in the *KDM5C* and variants assessed according to the American College of Medical Genetics Guidelines for interpretation of genomic sequence variants [16]. The study included ten male patients affected with Claes-Jensen syndrome and eight healthy females carrying pathogenic mutations in *KDM5C*. All male subjects were clinically confirmed to be affected by the Claes-Jensen syndrome. The age of the patients was recorded at the time of blood draw. The mutation status and demographics of all of the patients are shown in Table 1. These patients and carriers are members of three unrelated families for whom comprehensive clinical, molecular, and mutation assessments have been previously reported by Abidi et al. [17]. Controls were selected from our lab reference cohort of individuals with no known aberrant epigenomic change, including examination for imprinting defects, abnormal methylation pattern associated with other developmental syndromes, and representation of outlier profiles in principle component analysis. This reference cohort includes individuals that were previously selected from a larger cohort of about 1000 individuals across the broad range of age, sex, and ethnicity distribution.

**Table 1:** Clinical and molecular characteristics of male patients and female mutation carriers referred for methylation study

Sample ID	Sex	Age (years)	Disease status	Mutation	Cohort
3694a	M	28	Patient	c.4439_4440delAG; p.R1481GfsX9	Discovery/training
3695a	M	26	Patient	c.4439_4440delAG; p.R1481GfsX9	Discovery/training
3696a	F	51	Carrier	c.4439_4440delAG; p.R1481GfsX9	Training
12551D	F	55	Carrier	c.229G>A; p.A77T	Training
cms6013	M	37	Patient	c.229G>A; p.A77T	Testing
cms13123A	F	66	Carrier	c.229G>A; p.A77T	Testing
cms13755	M	13	Patient	c.229G>A; p.A77T	Discovery/training
cms13756	F	17	Carrier	c.229G>A; p.A77T	Testing
cms13757	F	39	Carrier	c.229G>A; p.A77T	Training
cms1179	F	54	Carrier	c.1510G>A; p.V504M	Training
cms1180	M	30	Patient	c.1510G>A; p.V504M	Discovery/training
cms1181	M	26	Patient	c.1510G>A; p.V504M	Testing
cms1224	F	54	Carrier	c.1510G>A; p.V504M	Training
cms1242	M	8	Patient	c.1510G>A; p.V504M	Discovery/training
cms1243	F	31	Carrier	c.1510G>A; p.V504M	Training
cms13185	M	2	Patient	c.1439C>T; p.P480L	Discovery/training
cms13186	M	6	Patient	c.1439C>T; p.P480L	Testing
cms4919B	M	42	Patient	c.1583+5G>A; p.E468GfsX2	Discovery/training

*fs* frameshift

### Methylation array and quality assessment

Genomic DNA was extracted from peripheral blood using standard techniques. Following bisulfite conversion, DNA methylation analysis was performed using the Illumina HumanMethylation450 bead chip (San Diego, CA), according to the manufacturer’s protocol at the Genetic and Molecular Epidemiology Laboratory at McMaster University and the London Health Sciences Molecular Genetic Laboratory. This array covers above 485,000 human genomic methylation CpG sites, including 99% of RefSeq genes and 96% of CpG islands. Methylated and unmethylated intensity data were generated as *idat* files and imported into R 3.4.0 for analysis. Normalization was performed using Illumina normalization method with background correction using *minfi* package [18]. Probes with detection *p* value > 0.01 were excluded from the downstream analysis. For further quality improvement, probes located on chromosomes X and Y, probes known to contain SNPs at the CpG interrogation or the single nucleotide extension, and probes known to cross-react with sex chromosomes were removed. As well, the samples representing discordance between the predicted and labeled sex were not used in the analysis. All of the samples were examined for genome-wide methylation density, and those deviating from the bimodal distribution were excluded. The methylation analysis of all of the controls in this study was performed in the same facility as patients, and the same data processing pipeline was used for all of them. A factor analysis using principle component analysis was performed to rule out the possibility of a batch effect or other sources of variability.

### Selection of discovery/training and testing cohorts and controls

**Fig. 1:**
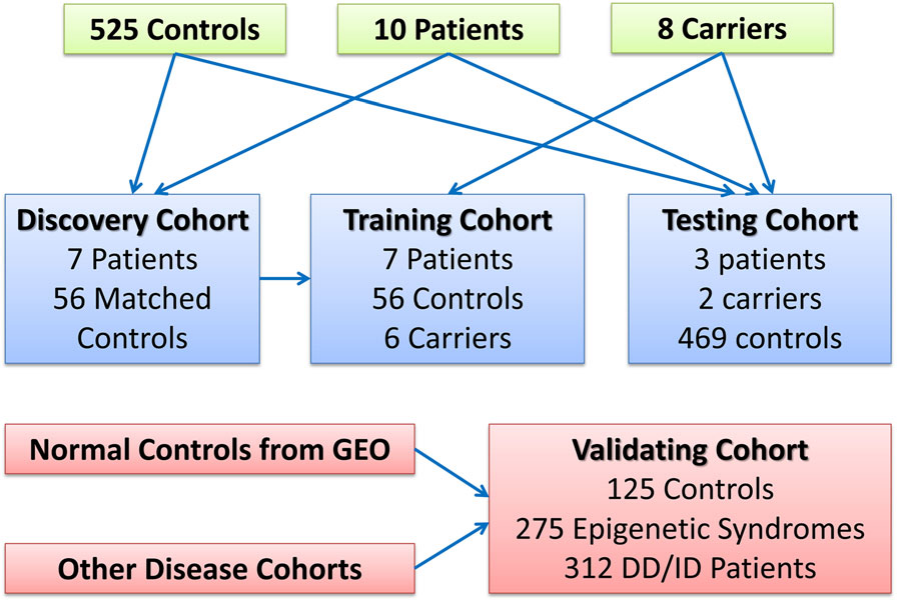
Schematic description of the study cohort

The identification of epi-signature was performed using a randomly selected 75% subset of the male patients (*discovery/training set*) using *caTools* package. The remaining male patients were used as a *testing cohort* to assess the performance of the classification model developed later in the study. Female carriers were divided into a training and testing cohort according to the same rule as affected males, but were only used for training and validating the classification model. For the patients in the discovery cohort (75% of affected males), a sex- and age-matched control group was selected from the reference control group using *MatchIt* package. This matched control group was used in both identifications of the epi-signature and training of the classification model (Table 1, Fig. 1).

### Identification of the epi-signature

The methylation levels for each probe were measured as beta value, calculated from the ratio of the methylated signals vs. the sum of unmethylated and methylated signals, ranging between 0 (no methylation) and 1 (full methylation). This value was used for biological interpretation and visualization. For the purpose of statistical analysis, wherever a normal distribution was required, beta values were transformed to *M* values using the following logit equation: log2(beta/(1 − beta)). To identify the differentially methylated probes, a linear regression modeling using the *limma* package was used [19]. The analysis was adjusted for blood cell type compositions predicted using *minfi* package. The generated *p* values were moderated using the *eBayes* function in *limma* package and corrected for multiple testing using Benjamini and Hochberg method as the default method implemented in the *limma* package. Probes with a corrected *p* value < 0.01 and a methylation difference greater than 10% were considered significant. The identified signature was examined using unsupervised hierarchical clustering to determine its ability in separating the patients from controls.

### Identification of the differentially methylated regions (DMRs)

To find genomic regions harboring methylation changes, a bump hunting approach was used by the *bumphunter* package [20]. The analysis considered regions with greater than 10% change in the overall methylation between cases and controls with gaps no more than 500 base pairs among neighboring CpGs. The 10% methylation cutoff was chosen to avoid reporting of methylation patterns with low-effect size or those influenced by variability in microarray technology. As suggested by the package, a 1000 bootstrapping procedure was performed to compute the family-wise error rate (FWER). We selected regions containing a minimum of three consecutive probes and FWER < 0.01. The identified regions were mapped to CpG islands and coding genes. *Gviz* package was used for visualization of the identified regions.

### Constructing and validating a classification model for Claes-Jensen syndrome

The identified signature was used to build a classification model for Claes-Jensen syndrome. *Caret* package [21] was used for feature selection from the signature. First, a receiver’s operating characteristic curve analysis was performed to identify the most differentiating probes. Those probes with full differentiation between cases and controls (area under the curve = 1.00) were retained. Next, pairwise correlations among the remaining probes were measured to identify and exclude the redundant signals with *R*-squared cutoff > 0.8. A multi-class support vector machine (SVM) with radial basis function kernel was trained on the remaining probes using *e1071* package. To determine the best hyperparameters and to measure the accuracy of the model, a tenfold cross-validation was performed. In this process, the training set was divided into tenfolds. Ninefolds were used for training the model and onefold for testing. After repeating this iteration for of all of the tenfolds, the mean accuracy was calculated and the hyperparameters with the optimal performance were selected. For every sample, the model was set to generate three classification scores between 0 and 1 as the probability of having a methylation profile related to full epi-signature as seen in affected patients, partial signature as seen in healthy carriers, and normal methylation profile as seen in controls. To assess the sensitivity of the model, the *testing cohort*, which was not used for identification of the signature or construction of the SVM, was supplied to the model. To determine the specificity, we supplied all of the healthy subjects, which were not used in the earlier stages of the study, to the model. We complemented this healthy cohort with a cohort of methylation database from healthy subjects downloaded from GEO (accession ID: GSE97362). To understand whether the signature of *KDM5C* mutation is sensitive to other medical conditions representing developmental delay and intellectual disabilities, we tested a group of patients with a confirmed clinical and molecular diagnosis of various diseases of such kind using the constructed model including patients with autism spectrum disorders, imprinting defect disorders, RASopathies, chromosomal aberrations, and Down syndrome. As well, it was tested whether this classifier is sensitive to other diseases of epigenomic machinery including ones with published epi-signatures. These included patients with *DNMT1*-associated autosomal dominant cerebellar ataxia, deafness, and narcolepsy, ATRX, and Floating-Harbor syndrome, collected from the Children’s Hospital of Eastern Ontario, and samples from patients with Saethre-Chotzen Syndrome, Coffin-Siris syndrome, Coffin-Lowry syndrome, Rett syndrome, Kabuki syndrome, and CHARGE syndrome, collected from the Greenwood Genetic Center. The same methylation array procedures as described earlier were conducted for them. The CHARGE and Kabuki cohorts were supplemented by methylation array files publically available from GEO (GSE97362). Files from patients with Sotos and Weaver syndromes were also downloaded from GEO (GSE74432) and added to this testing cohort.

## Results

### Description of the study cohort

The *discovery cohort* was composed of seven male patients affected with Claes-Jensen syndrome (mean age ± SD 21.2 ± 14.3). A group of 56 healthy male individuals (mean age ± SD 19.3 ± 12.2) from our reference cohort were matched with the patients for identification of the epi-signature. The *training cohort*, which was used for development of a classification model, was composed of the discovery cohort in addition to six healthy female carriers of the *KDM5C* mutations. The *testing cohort*, which was only used to measure the performance of the classification model, is composed of three affected male patients and two healthy female carriers. Table 1 shows the demographic and molecular characteristics of all of these subjects. Figure 1 shows a schematic representation of the overall study cohort.

### Epigenomic profiling of *KDM5C* mutation

From a total of 427,492 CpG probes in the Illumina Infinium methylation 450k array that passed the quality assessment, 1769 probes were found to have > 10% methylation difference between the affected male patients and controls with multiple testing corrected *p* value < 0.01 (equal to a nominal *p* value < ~0.00000001), adjusted for blood cell type compositions using *limma* regression modeling (Additional file 1: Table S1). Unsupervised hierarchical clustering generated by using these probes revealed a unique methylation profile, completely separating the patients from controls. Most of these probes were hypomethylated in the patients relative to the controls (1271 hypomethylated vs. 498 hypermethylated, Additional file 1: Table S1, Figure S1), and the majority (*n* = 1395) were located inside or nearby a CpG island. Also, a large proportion was located inside or nearby coding genes, a significant number of which contained multiple significant probes (Additional file 1: Table S1).

### Mapping of differentially methylated genomic regions (DMRs)

Using a “bump hunting” approach, we identified nine genomic coordinates containing a minimum of three CpG probes, an average regional methylation difference > 0.10, and a family-wise error rate (FWER) < 0.01 (Table 2). The vast majority of these regions overlap protein-coding genes, and all are found to be located on or in the vicinity of a CpG island. Except for a ~ 1.2-kb region on chromosome 15, all of the identified segments are hypomethylated in patients relative to controls. This segment is annotated to the first intron of *MIR9*-*3HG* and is the longest and one of the most differentially methylated regions identified in this study.

**Table 2:** Regions differentially methylated in Claes-Jensen syndrome

Chromosome	Start	End	Width (bps)	Methylation difference	Probe count	FWER	Overlapping gene	Distance to CpG island
chr15	89,919,993	89,921,182	1190	+ 0.28	8	0.001	*MIR9*-*3HG*	0
chr17	7,486,551	7,486,874	324	− 0.29	7	0.001	*MPDU1* (1121)^a^	0
chr6	164,092,410	164,093,099	690	− 0.32	6	0.001		0
chr13	113,242,878	113,243,141	264	− 0.33	3	0.004	*TUBGCP3* (396) ^a^	221
chr2	25,383,404	25,384,809	1406	− 0.26	7	0.005	*POMC*	0
chr5	176,559,334	176,559,563	230	− 0.33	3	0.005	*NSD1* (1270) ^a^	0
chr2	232,348,334	232,348,794	461	− 0.28	4	0.008		0
chr1	7,887,199	7,887,560	362	− 0.24	5	0.009	*PER3*	0
chr16	2,801,793	2,801,952	160	− 0.31	3	0.01	*SRRM2-AS1*	0

Methylation difference is calculated by subtracting average regional methylation levels in controls from the patients (patients − controls)

*FWER* family-wise error rate, *bps* base pairs

^a^Distance in base pair from the transcription start site

### Evaluation of healthy female carriers with *KDM5C* mutations using the epi-signature

**Fig. 2:**
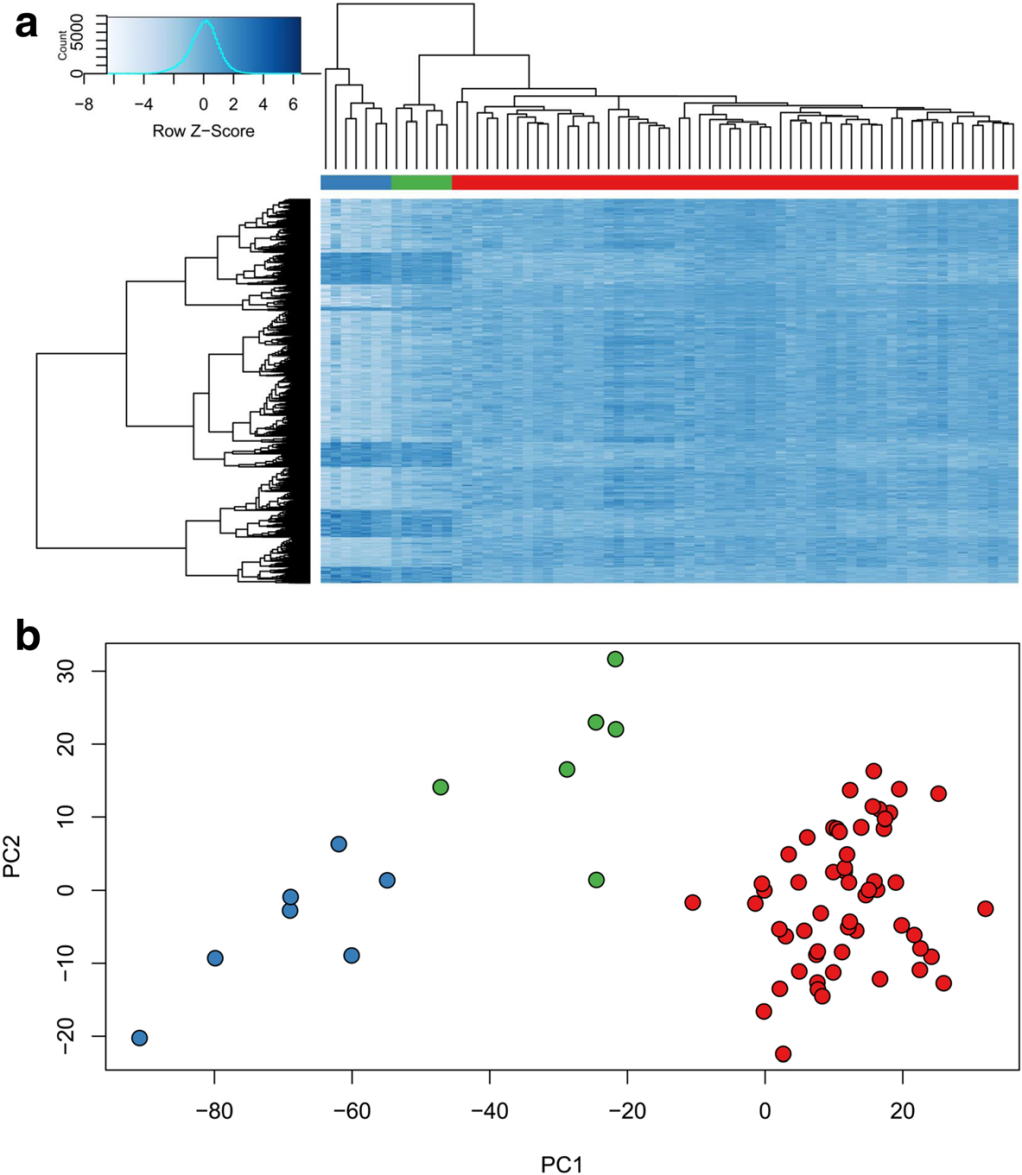
Clustering of the patients, carriers, and controls using the epi-signature: **a** unsupervised hierarchical clustering of patients, carriers, and normal controls shows two distinct clusters for patients (blue bar), and controls (red bar), and an intermediate cluster for the carriers (green bar). The rows represent the individual probes, and the columns represent the individual samples. Dark blue represents hypermethylation, and light blue represents hypomethylation in patients relative to the controls. **b** The first two components of the principal component analysis of patients (blue), carriers (green), and controls (red), based on the methylation status of the probes in the epi-signature, show a complete separation of patients from controls, while the carriers are placed in between the two

**Fig. 3:**
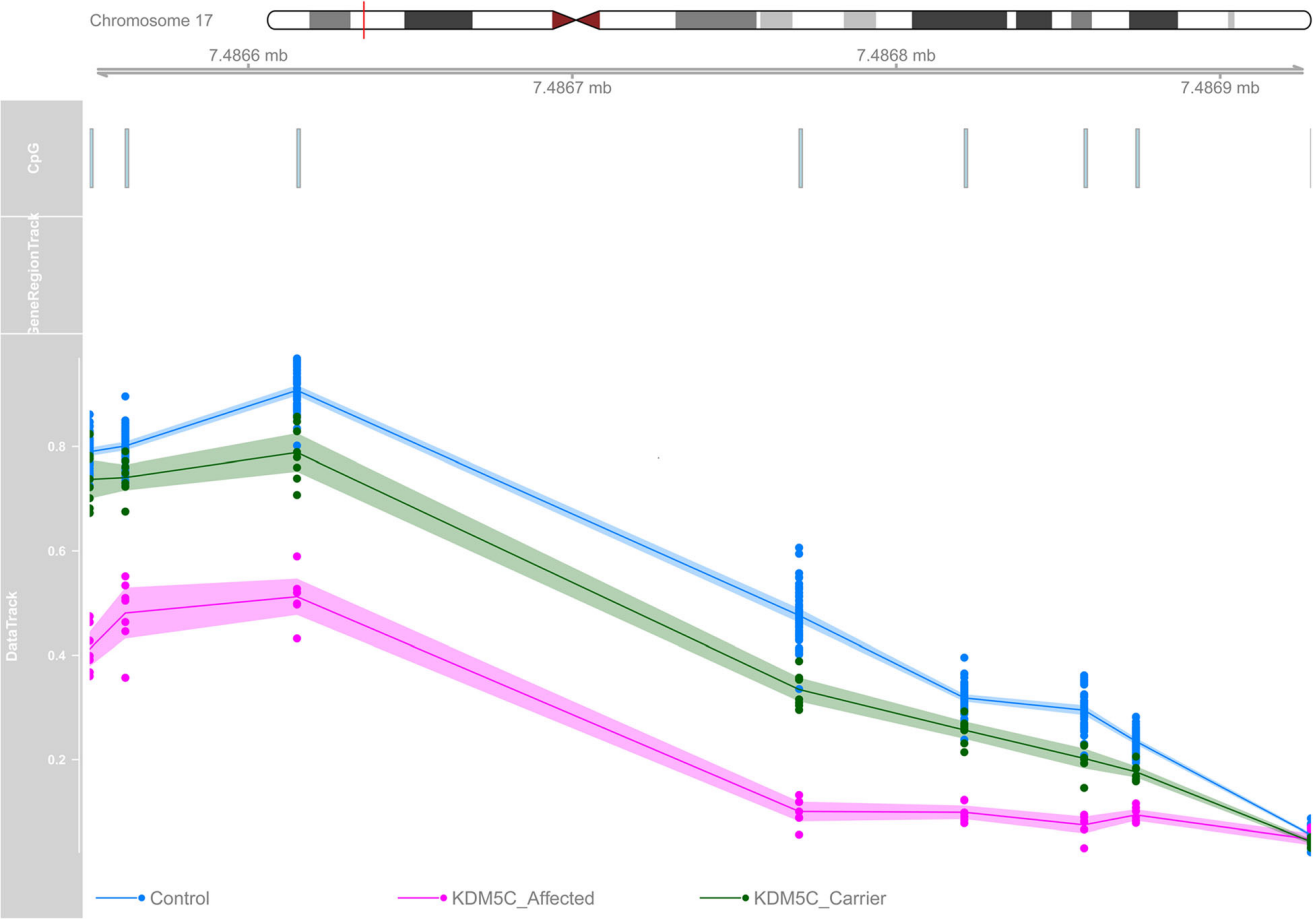
Hypomethylation of chr17:7486551-7486874: This segment is annotated to the promoter of *MPDU1*, which with an average hypomethylation of 29%, is one of the most differentially methylated regions between the patients (pink) and controls (blue). The inclusion of healthy carriers (green) generates a methylation pattern between the two groups. Track 1, chromosome ideogram; track 2, CpG probes; track 3, gene region; track 4, methylation level data; line, average methylation; shadow, 95% confidence interval; dots, methylation values from every single sample (0–1)

When healthy female carriers were added to the hierarchical clustering using the identified epi-signature of the patients, they did not group with either of the controls or the patients; rather, they represented an intermediate methylation pattern, residing them in between the two (Fig. 2a). A similar pattern was observed using the principal component analysis (Fig. 2b). Evaluation of the DMRs suggested a similar scenario, where the healthy mutation carriers showed an intermediate level of methylation as compared with the patients and controls for all of the identified regions (Fig. 3). These observations suggested that the epi-signature of the Claes-Jensen syndrome can be used for detection of both affected patients and healthy mutation carriers since it represents a specific pattern in each of these situations.

### Development and validation of a classification model for patients and carriers

**Fig. 4:**
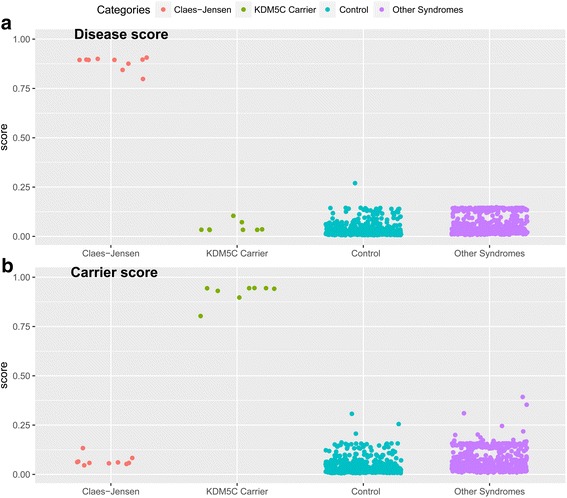
Probability scores generated by the classification model: A multi-class SVM classifier concurrently generates two scores for every subject as the probability of having a DNA methylation profile similar to patients with Claes-Jensen syndrome (**a**) and healthy carriers of *KDM5C* mutations (**b**). *y*-axis represents scores 0–1, with higher scores indicating a higher chance of carrying a methylation profile related to any of the two statuses. The *x*-axis represents the classification scores for 10 patients with Claes-Jensen syndrome, 8 healthy carriers of *KDM5C* mutations, a total of 650 normal controls, and 587 patients with other conditions that present with intellectual disability as described in the “Methods” and “Results” sections. By default, the SVM classifier defines a cutoff of 0.5 for predicting the class; however, the vast majority of the tested individuals received a score close to 0 or 1. Therefore, for the purpose of better visualization, the points are jittered. Every point represents the probability score received for a single sample. This figure represents scores obtained by both the subjects in the training and testing cohorts

To develop a classification model with the capacity of detecting both patients and carriers, a multi-class support vector machine (SVM) with radial basis function kernel was trained using a subset of 198 most differentiating and non-redundant probes selected from the epi-signature of Claes-Jensen syndrome (Additional file 1: Tables S2). The training was performed on the training cohort composed of 7 patients, 6 female carriers, and 56 controls. For every given subject, the model was set to generate three classification scores between 0 and 1 as the probability of having a methylation profile similar to those seen in patients, healthy carriers, and controls. A tenfold cross-validation of this model revealed an accuracy of 100%, and it correctly predicted the class of all of the subjects that were used for its training (Fig. 4).

Next, to determine the predictive power of this model on samples which were not used for its training, we supplied the model with the testing cohort (three patients and two carriers). These subjects were not used for the identification of the epi-signature or construction of the model. All of these samples received high scores for the correct class which they belonged to and low scores for the other two categories (Fig. 4). To estimate the specificity of our method, we used 469 healthy controls from our reference cohort that were not used earlier in this study. As well, we downloaded the methylation data from a set of 125 healthy subjects available publically from GEO (accession ID GSE97362). All of these samples received very low scores by our model for having a methylation profile as seen in either of the patients or the carriers (Fig. 4), suggesting that this model is 100% specific to the methylation profile of *KDM5C* mutation using these two large confirmation datasets.

### The classification model for Claes-Jensen syndrome is not sensitive to other diseases of epigenomic machinery or other DD/ID conditions

To determine whether this model can distinguish the epigenomic profile of *KDM5C* mutation from other syndromes resulting from the defects in epigenomic machinery, we supplied the model with a cohort of 275 samples, composed of patients with both clinical and molecular diagnoses of ATRX syndrome (*n* = 19), Floating-Harbor syndrome (*n* = 17), CHARGE syndrome (*n* = 83), *DNMT1*-associated autosomal dominant cerebellar ataxia, deafness, and narcolepsy (*n* = 5), Sotos syndrome (*n* = 38), Coffin-Lowry syndrome (*n* = 11), Coffin-Siris syndrome (*n* = 9), Kabuki syndrome (*n* = 44), Weaver syndrome (*n* = 7), Saethre-Chotzen syndrome (*n* = 25), and Rett syndrome (*n* = 17). All of these patients received low scores for having methylation profiles similar to those generated by *KDM5C* mutations (Fig. 4). Next, to determine the performance of this classifier in patients with developmental delay/intellectual disability (DD/ID) of other etiologies, the model was supplied with the methylation profile of 146 patients with autism spectrum disorders, 12 patients with various chromosomal abnormalities, 7 patients with Down syndrome, 50 patients with imprinting conditions (Angelman, Beckwith-Wiedemann, Prader-Willi syndrome), and 97 patients with various forms of RASopathies. All of these subjects had a confirmed clinical and molecular diagnosis of the mentioned conditions. Similar to the previous observations, all of these patients received low scores for the disease and carrier categories in our classification model, further suggesting that the epi-signature of *KDM5C* mutation is highly specific to Claes-Jensen type of intellectual disability (Fig. 4).

## Discussion

This study described a genome-wide DNA methylation signature specific to patients with Claes-Jensen syndrome, resulting from pathogenic mutations in *KDM5C.* It also demonstrated that a similar but less pronounced pattern of DNA methylation exists in healthy individuals who carry these pathogenic mutations. These methylation patterns seem to be highly specific to Claes-Jensen syndrome and not be sensitive to other diseases that result from the disruption of the epigenomic machinery or other conditions that present with DD/ID. These findings are consistent with the emerging evidence that unique germline DNA methylation patterns can be found where the epigenomic machinery is disrupted. This will have a direct clinical application in disease screening and differential diagnosis of Claes-Jensen syndrome.

The first published study to examine the DNA methylation changes in patients with Claes-Jensen syndrome was conducted by Grafodatskaya et al. [22] using HumanMethylation 27 BeadChip array, where the authors revealed recurrent global DNA hypomethylation changes in the peripheral blood sample of affected male individuals. The main limitation of that study was the use of a small cohort of controls and a low-resolution microarray platform (27,000 probes vs. 450,000 probes). Given the limited overlap of the probe sets between these two microarray technologies (< 5%), it is not possible to make a direct comparison between the findings of the two studies. However, both studies show a distinct pattern of DNA methylation in Claes-Jensen patients with *KDM5C* mutations, mainly composed of hypomethylated regions, as compared with the controls. This hypomethylation is most likely related to the loss of lysine demethylase activity of Kdm5c, which has been shown to be required for the maintenance of global DNA methylation [23]. However, the smaller degree of hypermethylation changes observed in this study might suggest that the consequence of the Kdm5c function loss is beyond its immediate targets, and the cascade of events that happen following its disruption may lead to a downstream imbalance in the regulation of many genes and genomic features.

Claes-Jensen syndrome is not the only condition caused by mutations in genes that are involved in regulating epigenetic machinery and chromatin regulation. More than 50 such disorders have been reported. One of the common clinical findings of these disorders is variable degrees of intellectual disability. The exact molecular mechanism involved in the pathophysiology of these disorders has not been well established. Accumulating evidence suggests that mutations affecting the function of these genes can result in altered epigenomic and transcriptional regulation of a large number of genes in the downstream pathways, which could eventually lead to the emergence of a variety of clinical features associated with these conditions. In line with this theory, our previous studies have identified specific epigenetic signatures for several disorders, including X-linked mental retardation with α-thalassemia, Floating-Harbor syndrome, DNMT1-associated cerebellar ataxia, deafness, and narcolepsy syndrome, as well as Kabuki syndrome [8–12]. These disorders are all caused by loss of function mutations in different genes involved in epigenomic machinery. Similarly, a unique methylation epi-signature has been reported for Sotos syndrome, caused by mutations in the *NSD1* gene, which encodes histone H3 lysine 36 methyltransferase [13]. Of interest, we also found a hypomethylated region in the promoter of the *NSD1* gene (mutations of which lead to Sotos syndrome) in patients with Claes-Jensen syndrome. This suggests that elucidation of the involved biological mechanisms will require a comprehensive pathway and system analysis. It is likely that a variety of shared pathways and gene networks, potentially in a hierarchical order, are mutually altered across many of these conditions. This raises the question whether the DNA methylation epi-signature of the *KDM5C* mutations is similar to other diseases that result from the defects in histone modifications, specifically dysregulation of H3K4 residue marks. We tried to answer this question by supplying a large cohort of such diseases, including 44 patients with Kabuki syndrome that results from mutations in *KMT2D* (another regulator of the H3K4) to our classification model for *KDM5C* epi-signature. All of these subjects received low scores for having a DNA methylation profile similar to the carriers or patients, suggesting that although the initial event might involve overlapping targets in the large network of histone modifier proteins, the downstream changes are unique to every condition.

A fascinating feature of Claes-Jensen syndrome is while the clinical consequence of Kdm5c disruption in humans is mainly neuropsychological, the biological features of Kdm5c function loss can be present in all cells. The disease is caused by germline mutations, and specific gene expression patterns have been observed in the peripheral blood of the affected individuals [24]. This suggests that changes in the DNA methylation, similar to gene expression changes, can be observed in multiple tissues without presenting a phenotype. If the disease DNA methylation pattern is established early in the development, it can be maintained by DNMT1 in multiple lineages including the peripheral blood [14]. This is consistent with our observation that the *KDM5C* mutations generated a specific DNA methylation profile in the peripheral blood of the patients and carriers, completely distinct from what can be observed in healthy individuals or those affected by other forms of DD/ID. Although it is not clear to what extent the changes that we observed in this study represent the defective biological processes that occur in the nervous system of the patients with this form of intellectual disability, they will have a great potential for clinical screening and molecular diagnosis.

The clinical diagnosis of Claes-Jensen syndrome is challenging by the fact that *KDM5C* mutations are responsible for only 1–4% of all types of X-linked inherited intellectual disabilities. The differential diagnosis is further complicated by the overlapping clinical features of this syndrome with the numerous DD/ID disorders that present with intellectual disability. In addition, the clinical phenotype associated with mutations in the *KDM5C* gene shows a degree of variability with regard to the facial dysmorphism and cognitive impairment [25], making the diagnosis difficult by merely relying on the clinical findings. Similarly, sequence variant screening does not always provide a definitive answer. Routine clinical molecular diagnosis is performed on exons and exon-intron boundaries, and variants in non-coding regions are not often screened (e.g., promoter, intron). Within coding regions, missense and in-frame indels are not easily interpreted and are most often classified as variants of unknown clinical significance (VUS). Classification and interpretation of such variants are a challenge in molecular diagnostics and genetic counseling, despite the fact that they can induce protein truncation through altering the protein synthesis, stability, post-translational modification, and interaction with other proteins, or induce haploinsufficiency through a dominant-negative effect [26]. As an example in this study cohort, without RNA analysis [17], it would not have been possible to establish a pathogenic status for the c.1583+5G>A variant that was found in patient cms4919B (Table 1). Our classification model based on DNA methylation that is presented in this study has an optimal performance in detecting both patients with Claes-Jensen syndrome and healthy carriers with pathogenic variants in *KDM5C*. Using multiple large cohorts of healthy control subjects and patients with various forms of DD/ID conditions, we demonstrated that this model is highly specific to the methylation profile generated by *KDM5C* mutations. This can be used as a clinical test for resolving the cases where sequence variant assessment alone could not provide a definitive answer. This method is particularly under development in other diseases that result from the disruption of epigenomic machinery [8–13].

While *KDM5C* sequencing remains the standard approach for molecular assessment of patients with suspected Claes-Jensen syndrome, a genomic DNA methylation analysis may be used to augment this approach. In particular, DNA methylation profiling can be useful for reclassification of VUSs that are commonly detected by sequencing in clinical laboratories. An alternative, broader approach may involve routine genomic DNA methylation screening of patients with a broad range of developmental disorders for which known DNA methylation defects are identified such as conditions due to imprinting defects (Prader-Willi, Angelman, Beckwith-Weidman, Silver-Russell syndromes), uniparental disomy, Fragile X syndrome, and disorders with known peripheral blood epi-signatures [14, 27]. Given the technical cost of this array-based approach is similar to the cost of an average single-gene molecular test, genomic DNA methylation profiling may have a potential to be applied as an initial screen to augment current genomic screening tests including CNV microarray testing and exome sequencing.

## Conclusion

In conclusion, this study reports a specific methylation “epi-signature” resulting from loss of function mutations in the *KDM5C* gene that can be used to diagnose males with X-linked intellectual disability and asymptomatic female carriers. Our highly specific classification model that is presented here for this condition will potentially have a direct application for the screening of patients with overlapping characteristics with Claes-Jensen syndrome and may help classify variants of the unknown clinical significance in the *KDM5C* gene. This unique DNA methylation epi-signature along with peripheral blood epi-signatures from other genetic conditions, imprinting disorders [28], Fragile X syndrome [29], and other diseases that remain to be discovered, provides an avenue for the implementation of genomic DNA methylation screening for routine clinical testing of patients with a broad range of hereditary developmental and/or intellectual disability disorders. Our classification algorithm using 198 CpGs can be used as a cost-effective targeted assay for screening of subjects suspected to have Claes-Jensen syndrome, for whom the sequence variant analysis is not conclusive. Alternatively, these subjects can be screened using a genome-wide approach if other epigenetic abnormalities are suspected or differential diagnosis is not possible merely based on clinical findings.

## Additional file

Additional file 1: **Table S1.** CpG probes differentially methylated between the affected males and controls (*n* = 1,769). Table S2. The most differentiating and non-redundant probes selected from the epi-signature of Claes-Jensen syndrome (*n* = 198). Figure S1. Volcano plot of the methylation analysis. (DOCX 1865 kb)

## Abbreviations

ATRX: Alpha-thalassemia/mental retardation X-linked
DD/ID: Developmental delay/intellectual disability
DNMT1: DNA methyltransferase 1
DNMT3B1: DNA cytosine-5 methyltransferase 3B isoform 1
FWER: Family-wise error rate
H3K4: Histone H3 lysine (K) 4
KDM5C: Lysine (K)-specific demethylase 5C
SNP: Single nucleotide polymorphism
SVM: Support vector machine
VUS: Variants of unknown significance
