# Supplementary material for: Peripheral blood epi-signature of Claes-Jensen syndrome enables sensitive and specific identification of patients and healthy carriers with pathogenic mutations in KDM5C
Source: Clin Epigenetics. 2018 Feb 14;10:21. doi: 10.1186/s13148-018-0453-8 (PMC5813334; doi:10.1186/s13148-018-0453-8)

Table S1- CpG probes differentially methylated between the affected males and controls (n=1,769)

| **Probe** | **Chr** | **Position** | **Gene** | **CpG island** | **Relation to island** | **Methylation difference** | **Corrected P-value** |
| --- | --- | --- | --- | --- | --- | --- | --- |
| cg12097325 | chr1 | 1076431 |  | chr1:1072369-1072847 | S_Shelf | 0.14 | 0.00030985 |
| cg11572942 | chr1 | 1656592 | *CDK11A* | chr1:1655395-1656231 | S_Shore | -0.11 | 0.004304734 |
| cg06203364 | chr1 | 2137307 | *C1orf86* | chr1:2136808-2137351 | Island | -0.12 | 0.00458184 |
| cg23626733 | chr1 | 2345398 | *PEX10* | chr1:2345891-2346156 | N_Shore | -0.10 | 0.001212209 |
| cg15090440 | chr1 | 3099233 | *PRDM16* | chr1:3102540-3103352 | N_Shelf | -0.19 | 0.000104771 |
| cg25165880 | chr1 | 6454326 | *ACOT7* | chr1:6452814-6454185 | S_Shore | -0.19 | 4.74E-10 |
| cg27539986 | chr1 | 6664103 | *KLHL21* | chr1:6661776-6663844 | S_Shore | -0.27 | 9.68E-12 |
| cg09159285 | chr1 | 6664134 | *KLHL21* | chr1:6661776-6663844 | S_Shore | -0.18 | 1.10E-09 |
| cg19884658 | chr1 | 6664268 | *KLHL21* | chr1:6661776-6663844 | S_Shore | -0.22 | 1.00E-11 |
| cg17328665 | chr1 | 7887199 | *PER3* | chr1:7887198-7887476 | Island | -0.26 | 0.001086364 |
| cg04725166 | chr1 | 7887271 | *PER3* | chr1:7887198-7887476 | Island | -0.19 | 0.000376081 |
| cg09168692 | chr1 | 7887560 | *PER3* | chr1:7887198-7887476 | S_Shore | -0.18 | 0.007124858 |
| cg24352878 | chr1 | 8772712 | *RERE* |  | OpenSea | -0.11 | 0.006089363 |
| cg04340595 | chr1 | 9789174 | *CLSTN1* | chr1:9790292-9790704 | N_Shore | 0.11 | 0.003430972 |
| cg04015962 | chr1 | 10949192 |  |  | OpenSea | 0.15 | 0.005301585 |
| cg03546360 | chr1 | 12675968 | *DHRS3* | chr1:12676983-12678642 | N_Shore | 0.12 | 0.003316857 |
| cg20464068 | chr1 | 14026054 | *PRDM2* | chr1:14026481-14027200 | N_Shore | -0.10 | 0.006713693 |
| cg02330214 | chr1 | 14026216 | *PRDM2* | chr1:14026481-14027200 | N_Shore | -0.14 | 0.000110691 |
| cg11344626 | chr1 | 14026360 | *PRDM2* | chr1:14026481-14027200 | N_Shore | -0.13 | 0.001270881 |
| cg24735235 | chr1 | 16163479 | *FLJ37453* | chr1:16163752-16164123 | N_Shore | -0.20 | 0.000127664 |
| cg04889800 | chr1 | 16163555 | *FLJ37453* | chr1:16163752-16164123 | N_Shore | -0.12 | 0.002829436 |
| cg19025461 | chr1 | 16163610 | *FLJ37453* | chr1:16163752-16164123 | N_Shore | -0.15 | 0.000411659 |
| cg05821634 | chr1 | 16164109 | *FLJ37453* | chr1:16163752-16164123 | Island | -0.11 | 0.000875469 |
| cg03978675 | chr1 | 16679500 | *FBXO42* | chr1:16678574-16679192 | S_Shore | -0.12 | 4.09E-05 |
| cg08947790 | chr1 | 17877451 | *ARHGEF10L* |  | OpenSea | 0.11 | 0.000779171 |
| cg08727996 | chr1 | 21880024 | *ALPL* |  | OpenSea | -0.11 | 0.008661459 |
| cg24117274 | chr1 | 21945805 | *RAP1GAP* | chr1:21948674-21949263 | N_Shelf | 0.16 | 0.009322557 |
| cg02623028 | chr1 | 23003569 |  | chr1:23003416-23003831 | Island | 0.10 | 7.61E-05 |
| cg27447006 | chr1 | 23763279 | *ASAP3* | chr1:23763154-23763971 | Island | 0.14 | 0.005039965 |
| cg19827787 | chr1 | 23763612 | *ASAP3* | chr1:23763154-23763971 | Island | 0.11 | 0.003221031 |
| cg24435747 | chr1 | 24514557 | *IL28RA* | chr1:24513540-24514477 | S_Shore | -0.13 | 0.006723268 |
| cg27298252 | chr1 | 24645380 | *GRHL3* | chr1:24648202-24648985 | N_Shelf | -0.16 | 0.003063819 |
| cg04471507 | chr1 | 26233376 | *STMN1* | chr1:26231950-26233624 | Island | -0.12 | 4.92E-05 |
| cg11709544 | chr1 | 26233404 | *STMN1* | chr1:26231950-26233624 | Island | -0.12 | 0.00091516 |
| cg01811796 | chr1 | 26233565 | *STMN1* | chr1:26231950-26233624 | Island | -0.10 | 0.001783603 |
| cg23323671 | chr1 | 26233623 | *STMN1* | chr1:26231950-26233624 | Island | -0.10 | 0.005657809 |
| cg08134856 | chr1 | 26233709 | *STMN1* | chr1:26231950-26233624 | S_Shore | -0.11 | 0.005151006 |
| cg01236573 | chr1 | 26248897 |  | chr1:26249205-26249613 | N_Shore | -0.17 | 6.01E-07 |
| cg25841309 | chr1 | 27248983 | *NUDC* | chr1:27248048-27248619 | S_Shore | -0.16 | 3.63E-09 |
| cg18936052 | chr1 | 28051702 | *FAM76A* | chr1:28052145-28053002 | N_Shore | -0.10 | 0.000211175 |
| cg02905426 | chr1 | 28051771 | *FAM76A* | chr1:28052145-28053002 | N_Shore | -0.10 | 3.87E-05 |
| cg24675730 | chr1 | 28994850 | *GMEB1* |  | OpenSea | -0.13 | 0.000312928 |
| cg20280170 | chr1 | 29101609 |  | chr1:29101790-29102069 | N_Shore | -0.10 | 0.002445078 |
| cg16554164 | chr1 | 32410678 |  | chr1:32410188-32410630 | S_Shore | -0.15 | 9.05E-07 |
| cg19163067 | chr1 | 36042438 | *TFAP2E* | chr1:36042432-36043444 | Island | -0.14 | 0.004553423 |
| cg17139085 | chr1 | 36787356 |  | chr1:36786500-36789402 | Island | -0.21 | 0.000105469 |
| cg22721579 | chr1 | 36787932 | *FAM176B* | chr1:36786500-36789402 | Island | -0.22 | 0.006302343 |
| cg09514174 | chr1 | 36916681 | *OSCP1* | chr1:36915796-36916324 | S_Shore | -0.12 | 0.00160563 |
| cg11596009 | chr1 | 36916744 | *OSCP1* | chr1:36915796-36916324 | S_Shore | -0.10 | 0.002911834 |
| cg24375409 | chr1 | 38200920 | *EPHA10* | chr1:38200919-38201200 | Island | 0.22 | 0.00064213 |
| cg04622024 | chr1 | 38201001 | *EPHA10* | chr1:38200919-38201200 | Island | 0.11 | 0.000428043 |
| cg11664987 | chr1 | 38201123 | *EPHA10* | chr1:38200919-38201200 | Island | 0.14 | 0.000156297 |
| cg09814029 | chr1 | 39212920 |  |  | OpenSea | 0.10 | 0.002584529 |
| cg16510040 | chr1 | 40042432 | *PABPC4* | chr1:40041317-40042667 | Island | -0.11 | 0.001698745 |
| cg10512203 | chr1 | 40780905 | *COL9A2* | chr1:40781004-40781445 | N_Shore | -0.11 | 0.000653164 |
| cg03387723 | chr1 | 41708464 | *SCMH1* | chr1:41706957-41708327 | S_Shore | -0.36 | 1.23E-20 |
| cg06520846 | chr1 | 42921582 | *PPCS* | chr1:42921716-42922575 | N_Shore | -0.10 | 6.09E-07 |
| cg06346081 | chr1 | 42921584 | *PPCS* | chr1:42921716-42922575 | N_Shore | -0.12 | 3.52E-06 |
| cg07865091 | chr1 | 43814306 | *MPL* | chr1:43814305-43815277 | Island | -0.12 | 0.007590942 |
| cg11839415 | chr1 | 43814764 | *MPL* | chr1:43814305-43815277 | Island | -0.10 | 0.003756222 |
| cg21219903 | chr1 | 43920090 | *HYI* | chr1:43919083-43919814 | S_Shore | -0.17 | 9.35E-12 |
| cg02283735 | chr1 | 44011587 | *PTPRF* |  | OpenSea | 0.17 | 4.48E-05 |
| cg15620905 | chr1 | 44024150 | *PTPRF* |  | OpenSea | 0.11 | 5.71E-06 |
| cg03578951 | chr1 | 44678791 | *DMAP1* | chr1:44679003-44679465 | N_Shore | -0.15 | 1.22E-06 |
| cg07113006 | chr1 | 45097282 | *RNF220* | chr1:45097281-45098049 | Island | -0.10 | 0.000304903 |
| cg02072975 | chr1 | 45249541 | *BEST4* | chr1:45249860-45250352 | N_Shore | -0.12 | 5.91E-05 |
| cg06766016 | chr1 | 45278971 | *BTBD19* | chr1:45278765-45279644 | Island | -0.18 | 0.000279461 |
| cg19530728 | chr1 | 45279132 | *BTBD19* | chr1:45278765-45279644 | Island | -0.13 | 0.000662215 |
| cg14611683 | chr1 | 45452580 | *EIF2B3* | chr1:45452037-45452444 | S_Shore | -0.21 | 1.91E-12 |
| cg16604218 | chr1 | 45452605 | *EIF2B3* | chr1:45452037-45452444 | S_Shore | -0.22 | 4.25E-13 |
| cg07424440 | chr1 | 45453003 | *EIF2B3* | chr1:45452037-45452444 | S_Shore | -0.17 | 1.39E-08 |
| cg13951491 | chr1 | 45793032 | *HPDL* | chr1:45792419-45793301 | Island | -0.22 | 9.09E-12 |
| cg16593917 | chr1 | 45793300 | *HPDL* | chr1:45792419-45793301 | Island | -0.11 | 8.81E-07 |
| cg24332710 | chr1 | 46016173 | *AKR1A1* | chr1:46016403-46016694 | N_Shore | -0.17 | 1.01E-08 |
| cg25369262 | chr1 | 46649132 | *TSPAN1* |  | OpenSea | -0.12 | 0.004545476 |
| cg18169994 | chr1 | 47329048 | *CYP4Z2P* |  | OpenSea | 0.12 | 0.003761423 |
| cg23803206 | chr1 | 47798847 | *CMPK1* | chr1:47799411-47800426 | N_Shore | -0.13 | 3.15E-05 |
| cg12640469 | chr1 | 53067717 | *GPX7* | chr1:53067880-53068608 | N_Shore | -0.10 | 0.007590942 |
| cg16646600 | chr1 | 54562543 | *C1orf83* |  | OpenSea | -0.10 | 0.007570132 |
| cg18384460 | chr1 | 63771428 |  |  | OpenSea | 0.13 | 0.007276008 |
| cg20457732 | chr1 | 71172486 |  |  | OpenSea | 0.34 | 3.94E-06 |
| cg27565650 | chr1 | 88151065 |  |  | OpenSea | -0.11 | 8.56E-05 |
| cg01128109 | chr1 | 89989507 | *LRRC8B* | chr1:89989810-89990986 | N_Shore | -0.15 | 8.73E-06 |
| cg10621610 | chr1 | 91175627 |  | chr1:91176404-91176701 | N_Shore | 0.12 | 0.000238922 |
| cg04603796 | chr1 | 91965822 | *CDC7* | chr1:91966263-91966999 | N_Shore | -0.12 | 0.008902373 |
| cg12403142 | chr1 | 92012408 |  | chr1:92012336-92012656 | Island | -0.24 | 0.003129279 |
| cg13639937 | chr1 | 92012655 |  | chr1:92012336-92012656 | Island | -0.12 | 0.004659661 |
| cg19245011 | chr1 | 92414221 | *BRDT* | chr1:92414718-92414986 | N_Shore | 0.11 | 0.009904616 |
| cg01515515 | chr1 | 92414295 | *BRDT* | chr1:92414718-92414986 | N_Shore | 0.20 | 0.006656071 |
| cg24517501 | chr1 | 92952702 | *GFI1* | chr1:92945907-92952609 | S_Shore | -0.13 | 0.000256797 |
| cg15512289 | chr1 | 97026071 |  |  | OpenSea | -0.15 | 0.005796608 |
| cg14994947 | chr1 | 101448135 |  |  | OpenSea | -0.13 | 0.000181879 |
| cg07133347 | chr1 | 107600762 | *PRMT6* | chr1:107599205-107600410 | S_Shore | -0.11 | 0.003769246 |
| cg11947712 | chr1 | 109203268 | *C1orf59* | chr1:109203593-109204378 | N_Shore | -0.19 | 6.56E-06 |
| cg24737783 | chr1 | 109204304 | *C1orf59* | chr1:109203593-109204378 | Island | -0.11 | 1.91E-10 |
| cg00328227 | chr1 | 109204325 | *C1orf59* | chr1:109203593-109204378 | Island | -0.26 | 5.00E-19 |
| cg10807101 | chr1 | 110282274 | *GSTM3* | chr1:110282351-110283306 | N_Shore | -0.12 | 0.005906041 |
| cg03257417 | chr1 | 111189871 |  |  | OpenSea | -0.14 | 0.001002218 |
| cg20432350 | chr1 | 112438321 | *KCND3* |  | OpenSea | 0.12 | 0.00036391 |
| cg07790079 | chr1 | 116021925 |  |  | OpenSea | 0.13 | 0.000343204 |
| cg25599129 | chr1 | 116022006 |  |  | OpenSea | 0.14 | 0.0003007 |
| cg19675142 | chr1 | 116107015 |  |  | OpenSea | 0.14 | 0.000102079 |
| cg07790752 | chr1 | 147101904 |  |  | OpenSea | -0.16 | 0.00556394 |
| cg13067553 | chr1 | 147782452 |  | chr1:147782066-147782473 | Island | 0.15 | 0.008768698 |
| cg13502125 | chr1 | 147826191 |  | chr1:147826167-147826388 | Island | 0.14 | 0.003555409 |
| cg05950212 | chr1 | 149860711 | *HIST2H2AB* | chr1:149857769-149859470 | S_Shore | -0.17 | 3.72E-06 |
| cg27541317 | chr1 | 149871625 | *BOLA1* | chr1:149871079-149871946 | Island | -0.15 | 1.68E-17 |
| cg24364827 | chr1 | 149871658 | *BOLA1* | chr1:149871079-149871946 | Island | -0.12 | 1.26E-10 |
| cg09045105 | chr1 | 149871945 | *BOLA1* | chr1:149871079-149871946 | Island | -0.24 | 1.04E-09 |
| cg25066665 | chr1 | 150335507 | *RPRD2* | chr1:150336831-150337289 | N_Shore | -0.31 | 0.00134127 |
| cg10589385 | chr1 | 150898437 | *SETDB1* | chr1:150898619-150898839 | N_Shore | -0.21 | 0.001287335 |
| cg04065086 | chr1 | 151104186 | *SEMA6C* | chr1:151103685-151106100 | Island | -0.13 | 0.000130135 |
| cg07371589 | chr1 | 151104367 | *SEMA6C* | chr1:151103685-151106100 | Island | -0.25 | 0.008256913 |
| cg27529647 | chr1 | 151300868 |  | chr1:151300522-151300724 | S_Shore | -0.19 | 1.35E-05 |
| cg10069121 | chr1 | 152009711 | *S100A11* | chr1:152008838-152009112 | S_Shore | -0.12 | 0.007363602 |
| cg23107878 | chr1 | 152161397 |  | chr1:152161321-152161928 | Island | -0.17 | 0.00580082 |
| cg00891995 | chr1 | 153113262 | *SPRR2C* |  | OpenSea | -0.14 | 0.000175794 |
| cg13392022 | chr1 | 154600693 | *ADAR* | chr1:154599763-154600505 | S_Shore | -0.16 | 2.58E-09 |
| cg16680214 | chr1 | 154839983 | *KCNN3* |  | OpenSea | 0.11 | 0.006634314 |
| cg23803022 | chr1 | 154908781 | *PMVK* | chr1:154908962-154909931 | N_Shore | -0.16 | 2.37E-10 |
| cg26189283 | chr1 | 155109378 | *RAG1AP1* | chr1:155108007-155108611 | S_Shore | -0.15 | 2.24E-05 |
| cg20253251 | chr1 | 155579369 | *MSTO1* | chr1:155579732-155580328 | N_Shore | -0.14 | 0.000498641 |
| cg06208270 | chr1 | 156046344 | *MEX3A* | chr1:156046343-156047384 | Island | -0.10 | 0.007471819 |
| cg06355129 | chr1 | 156646293 | *NES* | chr1:156646292-156647260 | Island | 0.14 | 0.000947455 |
| cg07580762 | chr1 | 161171810 | *NDUFS2* | chr1:161171809-161172256 | Island | -0.18 | 9.85E-09 |
| cg21691116 | chr1 | 161171819 | *NDUFS2* | chr1:161171809-161172256 | Island | -0.16 | 1.02E-06 |
| cg23915527 | chr1 | 161368787 |  | chr1:161368863-161369932 | N_Shore | -0.24 | 1.80E-07 |
| cg03441844 | chr1 | 161368947 |  | chr1:161368863-161369932 | Island | -0.30 | 2.31E-09 |
| cg15391651 | chr1 | 165132273 |  |  | OpenSea | -0.12 | 0.004836889 |
| cg19708554 | chr1 | 166136816 | *FAM78B* | chr1:166134258-166136448 | S_Shore | -0.11 | 0.001604689 |
| cg04275362 | chr1 | 167598560 | *RCSD1* | chr1:167599464-167599839 | N_Shore | -0.15 | 2.99E-05 |
| cg09372808 | chr1 | 167791030 | *ADCY10* | chr1:167789396-167789647 | S_Shore | 0.14 | 0.002083477 |
| cg05393023 | chr1 | 168194826 | *SFT2D2* | chr1:168194945-168195839 | N_Shore | -0.21 | 2.58E-05 |
| cg24317406 | chr1 | 174967663 | *CACYBP* | chr1:174968490-174969624 | N_Shore | -0.13 | 0.000428371 |
| cg04085339 | chr1 | 174967695 | *CACYBP* | chr1:174968490-174969624 | N_Shore | -0.21 | 3.36E-11 |
| cg26787020 | chr1 | 174968089 | *CACYBP* | chr1:174968490-174969624 | N_Shore | -0.28 | 7.33E-17 |
| cg24338780 | chr1 | 174968123 | *CACYBP* | chr1:174968490-174969624 | N_Shore | -0.11 | 1.62E-13 |
| cg16743289 | chr1 | 174968144 | *CACYBP* | chr1:174968490-174969624 | N_Shore | -0.28 | 6.63E-17 |
| cg21033965 | chr1 | 178455800 |  | chr1:178455799-178456286 | Island | -0.16 | 0.000358574 |
| cg09938479 | chr1 | 178455912 |  | chr1:178455799-178456286 | Island | -0.23 | 3.22E-05 |
| cg26004771 | chr1 | 178456093 |  | chr1:178455799-178456286 | Island | -0.23 | 0.000581525 |
| cg26915952 | chr1 | 178456270 |  | chr1:178455799-178456286 | Island | -0.25 | 3.94E-05 |
| cg13606991 | chr1 | 182556113 | *RNASEL* |  | OpenSea | 0.13 | 0.005082528 |
| cg12222949 | chr1 | 184005360 | *GLT25D2* | chr1:184005359-184006766 | Island | 0.17 | 0.001188105 |
| cg06811094 | chr1 | 200272112 |  | chr1:200271276-200271538 | S_Shore | 0.13 | 0.004641653 |
| cg22810423 | chr1 | 200272180 |  | chr1:200271276-200271538 | S_Shore | 0.11 | 0.003576507 |
| cg07138399 | chr1 | 200272215 |  | chr1:200271276-200271538 | S_Shore | 0.11 | 0.004233477 |
| cg19315653 | chr1 | 203096230 | *ADORA1* | chr1:203097233-203097496 | N_Shore | -0.11 | 0.000345655 |
| cg13858742 | chr1 | 204621504 | *LRRN2* |  | OpenSea | 0.11 | 0.000171706 |
| cg08128734 | chr1 | 206685423 | *RASSF5* | chr1:206680236-206681444 | S_Shelf | -0.12 | 0.004383805 |
| cg04290171 | chr1 | 207924482 | *CD46* | chr1:207925171-207925964 | N_Shore | -0.26 | 3.59E-08 |
| cg10210594 | chr1 | 208132787 |  | chr1:208132327-208133117 | Island | -0.14 | 0.001002218 |
| cg08422645 | chr1 | 210446261 |  |  | OpenSea | 0.11 | 0.00076084 |
| cg22332066 | chr1 | 210501621 | *HHAT* | chr1:210502133-210502793 | N_Shore | -0.11 | 7.97E-06 |
| cg01421119 | chr1 | 211555733 | *C1orf97* | chr1:211555939-211556332 | N_Shore | -0.15 | 0.004809099 |
| cg00567190 | chr1 | 211556508 | *C1orf97* | chr1:211555939-211556332 | S_Shore | -0.12 | 0.001042714 |
| cg02756939 | chr1 | 211556576 | *C1orf97* | chr1:211555939-211556332 | S_Shore | -0.13 | 0.000992457 |
| cg24642169 | chr1 | 212731669 |  | chr1:212731668-212732457 | Island | -0.14 | 1.64E-05 |
| cg14576824 | chr1 | 213224402 | *RPS6KC1* | chr1:213224611-213224867 | N_Shore | -0.26 | 3.36E-11 |
| cg13195486 | chr1 | 218457882 | *RRP15* | chr1:218458019-218458684 | N_Shore | -0.25 | 5.86E-08 |
| cg03329019 | chr1 | 221051117 |  | chr1:221051966-221053673 | N_Shore | -0.11 | 0.003439239 |
| cg11145399 | chr1 | 224034478 | *TP53BP2* | chr1:224033085-224034263 | S_Shore | -0.22 | 5.72E-09 |
| cg06939851 | chr1 | 226112534 | *PYCR2* | chr1:226111642-226112552 | Island | -0.23 | 9.21E-08 |
| cg20680802 | chr1 | 226299139 |  | chr1:226297287-226298586 | S_Shore | -0.11 | 0.004078874 |
| cg10778994 | chr1 | 226497636 | *LIN9* | chr1:226496361-226497501 | S_Shore | -0.10 | 3.72E-06 |
| cg04208928 | chr1 | 226498038 | *LIN9* | chr1:226496361-226497501 | S_Shore | -0.20 | 1.44E-07 |
| cg06829788 | chr1 | 228581405 | *TRIM11* | chr1:228582428-228582894 | N_Shore | -0.10 | 0.008837073 |
| cg26345105 | chr1 | 228634265 |  | chr1:228633382-228634127 | S_Shore | 0.10 | 0.001888565 |
| cg24070990 | chr1 | 228634724 |  | chr1:228633382-228634127 | S_Shore | 0.18 | 0.00070699 |
| cg01525538 | chr1 | 228785987 | *DUSP5P* | chr1:228785986-228786204 | Island | 0.10 | 0.006315866 |
| cg26340700 | chr1 | 231663886 | *TSNAX-DISC1* | chr1:231663999-231664608 | N_Shore | -0.10 | 2.07E-06 |
| cg05820241 | chr1 | 232442534 |  |  | OpenSea | 0.13 | 0.000658968 |
| cg09674468 | chr1 | 234300299 | *SLC35F3* |  | OpenSea | 0.10 | 0.004209196 |
| cg24455383 | chr1 | 243736307 | *AKT3* |  | OpenSea | 0.18 | 0.00114392 |
| cg00533390 | chr1 | 245028657 | *HNRNPU* | chr1:245026434-245028020 | S_Shore | -0.18 | 7.35E-08 |
| cg08529295 | chr1 | 245132782 | *EFCAB2* | chr1:245133028-245134711 | N_Shore | -0.16 | 0.002797527 |
| cg09368188 | chr1 | 245330018 | *KIF26B* |  | OpenSea | 0.11 | 0.005379037 |
| cg26060667 | chr1 | 247681242 |  | chr1:247681399-247681951 | N_Shore | -0.12 | 0.000703394 |
| cg22374474 | chr1 | 247681297 |  | chr1:247681399-247681951 | N_Shore | -0.10 | 0.000314102 |
| cg11166453 | chr1 | 247681781 |  | chr1:247681399-247681951 | Island | -0.22 | 0.000874901 |
| cg04899629 | chr1 | 247694531 | *LOC148824* | chr1:247694035-247694501 | S_Shore | -0.19 | 0.000634772 |
| cg14955916 | chr10 | 459968 | *DIP2C* | chr10:459692-460058 | Island | 0.10 | 8.86E-05 |
| cg22954052 | chr10 | 743392 |  | chr10:743240-743479 | Island | -0.12 | 0.00187577 |
| cg02939598 | chr10 | 11386395 |  | chr10:11386302-11386875 | Island | -0.20 | 0.006118334 |
| cg11103390 | chr10 | 11784920 | *ECHDC3* | chr10:11784407-11784937 | Island | -0.19 | 0.000405979 |
| cg17379932 | chr10 | 12085598 | *UPF2* | chr10:12084236-12085431 | S_Shore | -0.23 | 1.51E-10 |
| cg00513208 | chr10 | 12085641 | *UPF2* | chr10:12084236-12085431 | S_Shore | -0.26 | 2.73E-11 |
| cg10368935 | chr10 | 18240316 | *SLC39A12* |  | OpenSea | -0.11 | 0.001698365 |
| cg03174507 | chr10 | 21789582 |  | chr10:21788633-21789588 | Island | -0.11 | 0.000329971 |
| cg06874403 | chr10 | 21799047 |  | chr10:21797632-21799341 | Island | -0.15 | 1.91E-06 |
| cg04714110 | chr10 | 21799143 |  | chr10:21797632-21799341 | Island | -0.12 | 0.000149764 |
| cg04707519 | chr10 | 21799314 |  | chr10:21797632-21799341 | Island | -0.11 | 0.000470511 |
| cg05775862 | chr10 | 31026101 |  |  | OpenSea | 0.13 | 0.000354176 |
| cg06528823 | chr10 | 32303107 | *KIF5B* |  | OpenSea | -0.16 | 0.002207007 |
| cg05454446 | chr10 | 43277757 | *BMS1* | chr10:43277849-43278532 | N_Shore | -0.12 | 0.001262644 |
| cg00377344 | chr10 | 43857301 |  | chr10:43857486-43858205 | N_Shore | -0.11 | 0.000555694 |
| cg27438128 | chr10 | 43951555 | *ZNF487* | chr10:43950884-43951437 | S_Shore | -0.10 | 2.29E-06 |
| cg06012872 | chr10 | 45374841 |  |  | OpenSea | -0.11 | 0.004808704 |
| cg05187965 | chr10 | 45406764 | *TMEM72* |  | OpenSea | 0.14 | 0.006656071 |
| cg26686009 | chr10 | 45495739 | *C10orf25* | chr10:45496275-45496550 | N_Shore | -0.35 | 3.89E-11 |
| cg00848594 | chr10 | 45495782 | *C10orf25* | chr10:45496275-45496550 | N_Shore | -0.29 | 1.69E-08 |
| cg00899659 | chr10 | 45495971 | *C10orf25* | chr10:45496275-45496550 | N_Shore | -0.14 | 0.000660732 |
| cg26888227 | chr10 | 45697308 |  | chr10:45693718-45694890 | S_Shelf | 0.16 | 0.006126201 |
| cg27040468 | chr10 | 60456715 | *BICC1* |  | OpenSea | -0.12 | 0.006384409 |
| cg27357306 | chr10 | 63657059 |  |  | OpenSea | -0.13 | 0.000194276 |
| cg23661466 | chr10 | 63657173 |  |  | OpenSea | -0.14 | 0.000564863 |
| cg12845268 | chr10 | 63657363 |  |  | OpenSea | -0.19 | 1.40E-05 |
| cg03201337 | chr10 | 69609995 |  | chr10:69609477-69609722 | S_Shore | -0.10 | 0.00222967 |
| cg09978996 | chr10 | 70321668 | *TET1* | chr10:70320130-70320822 | S_Shore | 0.11 | 0.000206949 |
| cg15254238 | chr10 | 70321874 | *TET1* | chr10:70320130-70320822 | S_Shore | 0.10 | 0.000980375 |
| cg25926515 | chr10 | 70321889 | *TET1* | chr10:70320130-70320822 | S_Shore | 0.14 | 0.00214906 |
| cg06971773 | chr10 | 71169083 | *TACR2* | chr10:71168699-71168952 | S_Shore | -0.15 | 5.45E-05 |
| cg19225512 | chr10 | 72648693 | *PCBD1* | chr10:72647738-72648317 | S_Shore | -0.17 | 0.003673779 |
| cg04973995 | chr10 | 74057977 |  | chr10:74057609-74058037 | Island | -0.11 | 0.000265363 |
| cg18786171 | chr10 | 75935758 | *ADK* | chr10:75936217-75936726 | N_Shore | -0.12 | 0.002848975 |
| cg09454892 | chr10 | 76870850 | *SAMD8* | chr10:76871055-76871620 | N_Shore | -0.15 | 9.41E-07 |
| cg19094530 | chr10 | 79470169 |  | chr10:79470962-79471425 | N_Shore | 0.12 | 2.21E-05 |
| cg05377034 | chr10 | 81838185 | *C10orf57* | chr10:81838359-81839078 | N_Shore | -0.10 | 0.000959028 |
| cg02307823 | chr10 | 89675901 | *PTEN* |  | OpenSea | -0.16 | 0.005651493 |
| cg15972243 | chr10 | 90343435 | *RNLS* | chr10:90342528-90343221 | S_Shore | -0.12 | 0.002315215 |
| cg16060189 | chr10 | 94350577 |  | chr10:94351356-94351615 | N_Shore | -0.19 | 2.56E-05 |
| cg03771939 | chr10 | 96161916 | *TBC1D12* | chr10:96162023-96163327 | N_Shore | -0.12 | 0.002401469 |
| cg22792461 | chr10 | 97850322 |  | chr10:97849568-97850095 | S_Shore | -0.15 | 5.94E-05 |
| cg06658147 | chr10 | 97889407 | *ZNF518A* | chr10:97889636-97890412 | N_Shore | -0.15 | 0.001379342 |
| cg01056004 | chr10 | 98948259 |  | chr10:98945062-98946239 | S_Shelf | 0.11 | 0.004151465 |
| cg17231677 | chr10 | 102671993 | *FAM178A* | chr10:102672367-102673159 | N_Shore | -0.14 | 0.00023054 |
| cg15302379 | chr10 | 102821848 | *KAZALD1* | chr10:102820488-102822874 | Island | -0.12 | 0.002868363 |
| cg13269555 | chr10 | 102822002 | *KAZALD1* | chr10:102820488-102822874 | Island | -0.10 | 0.002629944 |
| cg24381155 | chr10 | 103289745 | *BTRC* |  | OpenSea | -0.13 | 0.002478967 |
| cg08757448 | chr10 | 104613767 | *C10orf32* | chr10:104614000-104614326 | N_Shore | -0.12 | 1.66E-05 |
| cg12662887 | chr10 | 105343920 | *NEURL* | chr10:105344173-105345039 | N_Shore | -0.23 | 2.65E-06 |
| cg17201651 | chr10 | 111683631 | *XPNPEP1* | chr10:111682775-111683483 | S_Shore | -0.15 | 0.000211524 |
| cg17093267 | chr10 | 111683654 | *XPNPEP1* | chr10:111682775-111683483 | S_Shore | -0.18 | 8.31E-07 |
| cg03962527 | chr10 | 111766879 | *ADD3* | chr10:111767087-111768355 | N_Shore | -0.17 | 0.000306099 |
| cg20925954 | chr10 | 112256640 | *DUSP5* | chr10:112257163-112258684 | N_Shore | 0.11 | 0.001188826 |
| cg15667844 | chr10 | 112256729 | *DUSP5* | chr10:112257163-112258684 | N_Shore | 0.11 | 0.001067087 |
| cg05141574 | chr10 | 112290177 |  | chr10:112289947-112290305 | Island | 0.11 | 1.15E-05 |
| cg11967480 | chr10 | 112290198 |  | chr10:112289947-112290305 | Island | 0.13 | 2.94E-09 |
| cg04917197 | chr10 | 112290331 |  | chr10:112289947-112290305 | S_Shore | 0.11 | 4.95E-06 |
| cg08877357 | chr10 | 113120532 |  |  | OpenSea | -0.19 | 0.001429199 |
| cg19223579 | chr10 | 115370894 | *NRAP* |  | OpenSea | 0.12 | 1.67E-05 |
| cg08354406 | chr10 | 115370978 | *NRAP* |  | OpenSea | 0.12 | 1.62E-05 |
| cg09656541 | chr10 | 115932823 | *C10orf118* | chr10:115933379-115934262 | N_Shore | 0.22 | 0.003528985 |
| cg14351425 | chr10 | 120968739 | *GRK5* | chr10:120966127-120967623 | S_Shore | 0.16 | 7.72E-05 |
| cg26203879 | chr10 | 120968844 | *GRK5* | chr10:120966127-120967623 | S_Shore | 0.14 | 0.002627689 |
| cg09859240 | chr10 | 121633447 | *C10orf119* | chr10:121631654-121633104 | S_Shore | -0.17 | 4.85E-05 |
| cg16273546 | chr10 | 123070392 |  |  | OpenSea | -0.23 | 0.00177795 |
| cg22633036 | chr10 | 123355576 | *FGFR2* | chr10:123356616-123358285 | N_Shore | 0.24 | 0.001442611 |
| cg11430259 | chr10 | 123355748 | *FGFR2* | chr10:123356616-123358285 | N_Shore | 0.30 | 0.0044592 |
| cg02210151 | chr10 | 123356041 | *FGFR2* | chr10:123356616-123358285 | N_Shore | 0.19 | 0.001950353 |
| cg17681491 | chr10 | 123356205 | *FGFR2* | chr10:123356616-123358285 | N_Shore | 0.13 | 0.000167622 |
| cg18566515 | chr10 | 123356236 | *FGFR2* | chr10:123356616-123358285 | N_Shore | 0.26 | 0.001781229 |
| cg24181174 | chr10 | 123900861 | *TACC2* |  | OpenSea | -0.11 | 0.005086933 |
| cg03804621 | chr10 | 124638756 | *FAM24B* | chr10:124638743-124639793 | Island | -0.20 | 2.49E-07 |
| cg02588889 | chr10 | 124829211 |  |  | OpenSea | -0.11 | 0.00546612 |
| cg05090351 | chr10 | 126851162 |  | chr10:126847224-126851327 | Island | -0.14 | 0.00661147 |
| cg17191109 | chr10 | 126851326 |  | chr10:126847224-126851327 | Island | -0.15 | 3.31E-05 |
| cg05189127 | chr10 | 127059333 |  |  | OpenSea | 0.10 | 0.00571903 |
| cg19049077 | chr10 | 127407606 | *C10orf137* | chr10:127407627-127408566 | N_Shore | -0.11 | 4.31E-05 |
| cg26201213 | chr10 | 131265796 | *MGMT* | chr10:131264948-131265710 | S_Shore | -0.11 | 1.23E-06 |
| cg27102141 | chr10 | 131843798 |  | chr10:131843516-131844219 | Island | 0.12 | 0.002984452 |
| cg13221347 | chr10 | 134062614 | *STK32C* |  | OpenSea | 0.15 | 0.009994997 |
| cg10825471 | chr10 | 134122566 | *STK32C* | chr10:134120447-134122422 | S_Shore | -0.12 | 0.000211057 |
| cg17301379 | chr10 | 134622602 |  | chr10:134622423-134622641 | Island | 0.12 | 0.001831036 |
| cg00056497 | chr10 | 134785521 |  |  | OpenSea | 0.14 | 0.007930758 |
| cg19966745 | chr10 | 134965215 |  |  | OpenSea | 0.13 | 4.49E-05 |
| cg15013527 | chr11 | 316339 |  | chr11:315739-316539 | Island | 0.14 | 0.002658183 |
| cg21565415 | chr11 | 618993 | *MUPCDH* | chr11:617226-617797 | S_Shore | 0.16 | 0.000158728 |
| cg04993130 | chr11 | 726759 | *EPS8L2* | chr11:725596-726870 | Island | -0.11 | 0.005651493 |
| cg14909179 | chr11 | 882687 | *CHID1* | chr11:881368-881666 | S_Shore | -0.14 | 0.00100243 |
| cg15352671 | chr11 | 1331497 | *LOC255512* | chr11:1330390-1331498 | Island | -0.21 | 1.22E-10 |
| cg22424444 | chr11 | 1331736 | *LOC255512* | chr11:1330390-1331498 | S_Shore | -0.25 | 2.31E-06 |
| cg04699162 | chr11 | 1331784 | *LOC255512* | chr11:1330390-1331498 | S_Shore | -0.19 | 4.06E-05 |
| cg13858747 | chr11 | 1331831 | *LOC255512* | chr11:1330390-1331498 | S_Shore | -0.15 | 4.31E-05 |
| cg22079043 | chr11 | 1785631 | *CTSD* | chr11:1784805-1785553 | S_Shore | -0.21 | 4.81E-08 |
| cg20973931 | chr11 | 1785695 | *CTSD* | chr11:1784805-1785553 | S_Shore | -0.10 | 2.03E-07 |
| cg12007048 | chr11 | 1785701 | *CTSD* | chr11:1784805-1785553 | S_Shore | -0.12 | 4.41E-05 |
| cg15017982 | chr11 | 1785723 | *CTSD* | chr11:1784805-1785553 | S_Shore | -0.17 | 7.41E-06 |
| cg14250138 | chr11 | 1785804 | *CTSD* | chr11:1784805-1785553 | S_Shore | -0.15 | 3.49E-06 |
| cg16133872 | chr11 | 1918744 |  |  | OpenSea | -0.20 | 4.09E-05 |
| cg09055519 | chr11 | 1918755 |  |  | OpenSea | -0.19 | 0.000276855 |
| cg11955198 | chr11 | 1918783 |  |  | OpenSea | -0.18 | 0.000376081 |
| cg11744767 | chr11 | 2907672 | *CDKN1C* | chr11:2907308-2907675 | Island | -0.10 | 0.000649603 |
| cg22022957 | chr11 | 2908102 | *CDKN1C* | chr11:2905023-2907024 | S_Shore | -0.15 | 0.002401469 |
| cg26665035 | chr11 | 2919763 | *SLC22A18AS* | chr11:2923301-2923817 | N_Shelf | 0.12 | 6.13E-06 |
| cg21245372 | chr11 | 3819539 | *PGAP2* | chr11:3818447-3819307 | S_Shore | -0.11 | 1.16E-08 |
| cg27603015 | chr11 | 7614420 | *PPFIBP2* |  | OpenSea | -0.16 | 0.000875469 |
| cg09636245 | chr11 | 7699330 |  | chr11:7694711-7695685 | S_Shelf | 0.11 | 0.001244009 |
| cg11132204 | chr11 | 9405884 | *IPO7* | chr11:9406108-9406702 | N_Shore | -0.15 | 3.02E-05 |
| cg26262049 | chr11 | 10628012 | *MRVI1* |  | OpenSea | -0.13 | 0.007570132 |
| cg12487162 | chr11 | 12845864 | *TEAD1* |  | OpenSea | 0.14 | 0.003728576 |
| cg06081951 | chr11 | 14991413 | *CALCA* | chr11:14993452-14993661 | N_Shelf | 0.12 | 9.92E-05 |
| cg18089569 | chr11 | 14996490 |  | chr11:14995128-14995908 | S_Shore | -0.10 | 0.001888565 |
| cg11966998 | chr11 | 15692519 |  |  | OpenSea | 0.15 | 0.000581525 |
| cg12302402 | chr11 | 18230629 | *LOC494141* | chr11:18230619-18230906 | Island | 0.13 | 0.00237063 |
| cg26999154 | chr11 | 43291043 |  |  | OpenSea | 0.20 | 0.000361953 |
| cg25317631 | chr11 | 44541905 |  | chr11:44541537-44541956 | Island | 0.11 | 0.000810317 |
| cg19126615 | chr11 | 47290188 | *MADD* | chr11:47291200-47291858 | N_Shore | -0.12 | 0.0016724 |
| cg13526040 | chr11 | 57093322 | *TNKS1BP1* | chr11:57091614-57092831 | S_Shore | -0.15 | 0.000654002 |
| cg25628989 | chr11 | 57093345 | *TNKS1BP1* | chr11:57091614-57092831 | S_Shore | -0.19 | 2.94E-08 |
| cg13305186 | chr11 | 57508416 | *TMX2* | chr11:57508730-57509569 | N_Shore | -0.11 | 0.000158728 |
| cg21488132 | chr11 | 57508545 | *C11orf31* | chr11:57508730-57509569 | N_Shore | -0.10 | 0.000179701 |
| cg00325125 | chr11 | 58736081 |  |  | OpenSea | 0.10 | 0.007044275 |
| cg18852692 | chr11 | 58830191 |  | chr11:58830063-58830739 | Island | 0.11 | 0.000399145 |
| cg09973514 | chr11 | 58830424 |  | chr11:58830063-58830739 | Island | 0.22 | 5.17E-05 |
| cg24985772 | chr11 | 58830610 |  | chr11:58830063-58830739 | Island | 0.11 | 0.000683098 |
| cg12265130 | chr11 | 59437344 | *PATL1* | chr11:59436201-59437232 | S_Shore | -0.21 | 8.85E-08 |
| cg12828018 | chr11 | 59437416 | *PATL1* | chr11:59436201-59437232 | S_Shore | -0.27 | 1.73E-10 |
| cg17379860 | chr11 | 61159602 | *TMEM216* | chr11:61159836-61160285 | N_Shore | -0.25 | 1.77E-14 |
| cg16316624 | chr11 | 61159649 | *TMEM216* | chr11:61159836-61160285 | N_Shore | -0.14 | 1.57E-13 |
| cg20208600 | chr11 | 61159687 | *TMEM216* | chr11:61159836-61160285 | N_Shore | -0.16 | 1.75E-10 |
| cg02315732 | chr11 | 61732658 | *FTH1* | chr11:61734760-61735932 | N_Shelf | -0.10 | 0.005200988 |
| cg03433986 | chr11 | 62477624 | *BSCL2* | chr11:62476770-62477481 | S_Shore | -0.11 | 1.64E-07 |
| cg17372806 | chr11 | 63448356 | *RTN3* | chr11:63448704-63449386 | N_Shore | -0.10 | 3.14E-05 |
| cg17590162 | chr11 | 63827361 | *MACROD1* |  | OpenSea | 0.12 | 0.006931791 |
| cg10612274 | chr11 | 63827432 | *MACROD1* |  | OpenSea | 0.14 | 0.000287183 |
| cg18545771 | chr11 | 63827879 | *MACROD1* |  | OpenSea | 0.10 | 0.000411943 |
| cg24147428 | chr11 | 65409760 | *SIPA1* | chr11:65409636-65410127 | Island | -0.17 | 0.002560841 |
| cg06456738 | chr11 | 66361081 | *CCDC87* | chr11:66360097-66360834 | S_Shore | -0.11 | 0.000236899 |
| cg18498241 | chr11 | 69286352 |  | chr11:69285724-69286479 | Island | 0.10 | 0.002720643 |
| cg25885280 | chr11 | 70760166 | *SHANK2* |  | OpenSea | 0.13 | 0.004482866 |
| cg21806242 | chr11 | 72532891 | *ATG16L2* | chr11:72532612-72533774 | Island | -0.12 | 0.00148432 |
| cg16028555 | chr11 | 75922172 |  | chr11:75922169-75922376 | Island | 0.11 | 0.007937674 |
| cg13529291 | chr11 | 75922323 |  | chr11:75922169-75922376 | Island | 0.11 | 0.006297916 |
| cg23555155 | chr11 | 86142104 |  | chr11:86142379-86142676 | N_Shore | -0.11 | 4.09E-05 |
| cg09610772 | chr11 | 86142407 |  | chr11:86142379-86142676 | Island | -0.24 | 2.60E-08 |
| cg18725375 | chr11 | 86142478 |  | chr11:86142379-86142676 | Island | -0.24 | 3.58E-05 |
| cg15146462 | chr11 | 86142587 |  | chr11:86142379-86142676 | Island | -0.31 | 1.19E-06 |
| cg08591668 | chr11 | 94150725 | *MRE11A* |  | OpenSea | -0.12 | 0.008488013 |
| cg10001186 | chr11 | 94882829 |  | chr11:94883335-94883565 | N_Shore | -0.17 | 3.28E-05 |
| cg26773791 | chr11 | 94883242 |  | chr11:94883335-94883565 | N_Shore | -0.12 | 0.000797931 |
| cg09527615 | chr11 | 94883350 |  | chr11:94883335-94883565 | Island | -0.18 | 0.003201432 |
| cg06716182 | chr11 | 96074944 | *MAML2* |  | OpenSea | 0.13 | 0.001133568 |
| cg03331229 | chr11 | 102821111 | *MMP13* |  | OpenSea | -0.13 | 0.000413956 |
| cg06978117 | chr11 | 111155014 | *C11orf53* |  | OpenSea | 0.15 | 0.000937332 |
| cg03554573 | chr11 | 111155021 | *C11orf53* |  | OpenSea | 0.15 | 0.001188208 |
| cg22879583 | chr11 | 111750412 | *FDXACB1* | chr11:111749570-111750078 | S_Shore | -0.19 | 1.65E-13 |
| cg26312542 | chr11 | 112038104 | *TEX12* |  | OpenSea | -0.24 | 6.34E-07 |
| cg07809301 | chr11 | 112038173 | *TEX12* |  | OpenSea | -0.18 | 0.000561919 |
| cg21127537 | chr11 | 113184936 | *TTC12* | chr11:113185332-113185663 | N_Shore | -0.14 | 9.95E-07 |
| cg02121547 | chr11 | 113649020 | *CLDN25* |  | OpenSea | -0.12 | 0.004365243 |
| cg19299755 | chr11 | 116706051 |  | chr11:116706481-116706910 | N_Shore | -0.13 | 0.007586776 |
| cg03044513 | chr11 | 116706153 |  | chr11:116706481-116706910 | N_Shore | -0.10 | 0.00424147 |
| cg02431260 | chr11 | 124628888 | *ESAM* | chr11:124628367-124629590 | Island | -0.17 | 5.59E-06 |
| cg24758816 | chr11 | 125756762 | *HYLS1* | chr11:125757055-125758107 | N_Shore | -0.11 | 0.000452775 |
| cg07085632 | chr11 | 125974404 |  |  | OpenSea | 0.10 | 0.009134424 |
| cg10499172 | chr11 | 126173246 | *DCPS* | chr11:126173539-126174042 | N_Shore | -0.12 | 0.008471716 |
| cg17631150 | chr11 | 126173261 | *DCPS* | chr11:126173539-126174042 | N_Shore | -0.14 | 0.000662215 |
| cg20559217 | chr11 | 126173516 | *DCPS* | chr11:126173539-126174042 | N_Shore | -0.16 | 1.30E-05 |
| cg22911867 | chr11 | 126173548 | *DCPS* | chr11:126173539-126174042 | Island | -0.16 | 7.40E-08 |
| cg03382250 | chr11 | 126471892 | *KIRREL3* |  | OpenSea | 0.16 | 4.25E-06 |
| cg20358011 | chr11 | 126581947 | *KIRREL3* |  | OpenSea | -0.13 | 0.00071739 |
| cg10647465 | chr11 | 126582242 | *KIRREL3* |  | OpenSea | -0.14 | 0.001188113 |
| cg02400092 | chr11 | 130185384 | *ZBTB44* | chr11:130183948-130184668 | S_Shore | -0.19 | 1.43E-05 |
| cg14482569 | chr11 | 130185651 | *ZBTB44* | chr11:130183948-130184668 | S_Shore | -0.17 | 9.89E-07 |
| cg05227350 | chr11 | 130185670 | *ZBTB44* | chr11:130183948-130184668 | S_Shore | -0.16 | 1.08E-06 |
| cg19558933 | chr11 | 134632242 |  | chr11:134632045-134632987 | Island | -0.37 | 0.000170698 |
| cg23515411 | chr11 | 134632361 |  | chr11:134632045-134632987 | Island | -0.23 | 3.50E-07 |
| cg05962028 | chr12 | 214000 | *IQSEC3* | chr12:213763-214227 | Island | 0.10 | 0.00662417 |
| cg14760797 | chr12 | 753199 | *NINJ2* | chr12:751752-752014 | S_Shore | -0.17 | 0.002043708 |
| cg06708720 | chr12 | 1099075 | *ERC1* | chr12:1099557-1100785 | N_Shore | -0.13 | 0.000135043 |
| cg23390118 | chr12 | 2339513 | *CACNA1C* | chr12:2339162-2339615 | Island | -0.13 | 0.000715748 |
| cg09576209 | chr12 | 2339614 | *CACNA1C* | chr12:2339162-2339615 | Island | -0.12 | 0.002374829 |
| cg18816701 | chr12 | 2800055 | *CACNA1C* | chr12:2800139-2801062 | N_Shore | -0.12 | 2.31E-06 |
| cg16257219 | chr12 | 3069765 | *TEAD4* | chr12:3067960-3069444 | S_Shore | 0.14 | 2.80E-08 |
| cg01039401 | chr12 | 3070406 | *TEAD4* | chr12:3067960-3069444 | S_Shore | 0.16 | 1.84E-08 |
| cg14976741 | chr12 | 4872921 | *GALNT8* |  | OpenSea | 0.12 | 0.000340758 |
| cg11808633 | chr12 | 6730599 | *LPAR5* | chr12:6729248-6730310 | S_Shore | -0.12 | 0.000670405 |
| cg03621974 | chr12 | 7650334 | *CD163* |  | OpenSea | -0.13 | 0.005291608 |
| cg09912793 | chr12 | 9066382 | *PHC1* | chr12:9066946-9067480 | N_Shore | -0.11 | 0.001871852 |
| cg21250433 | chr12 | 10283763 | *CLEC7A* |  | OpenSea | -0.13 | 0.00937119 |
| cg19360852 | chr12 | 12848977 | *GPR19* | chr12:12849067-12849270 | N_Shore | -0.13 | 4.62E-08 |
| cg18048953 | chr12 | 12867753 |  | chr12:12869798-12871248 | N_Shelf | -0.14 | 0.000256797 |
| cg05693982 | chr12 | 12940390 | *APOLD1* | chr12:12939713-12940391 | Island | -0.13 | 7.63E-06 |
| cg27464065 | chr12 | 12940610 | *APOLD1* | chr12:12939713-12940391 | S_Shore | -0.16 | 8.97E-08 |
| cg15883853 | chr12 | 13080282 |  |  | OpenSea | -0.17 | 0.00070292 |
| cg14754427 | chr12 | 16135308 | *DERA* |  | OpenSea | -0.14 | 0.000347982 |
| cg02723291 | chr12 | 22777465 | *ETNK1* | chr12:22777988-22778301 | N_Shore | -0.18 | 1.77E-05 |
| cg24850296 | chr12 | 25707569 | *IFLTD1* |  | OpenSea | -0.10 | 0.004862017 |
| cg24428144 | chr12 | 28180450 |  |  | OpenSea | -0.12 | 0.008431945 |
| cg02376887 | chr12 | 29542219 |  | chr12:29542218-29542838 | Island | -0.30 | 0.000743639 |
| cg22717478 | chr12 | 29542598 |  | chr12:29542218-29542838 | Island | -0.44 | 1.45E-05 |
| cg08198187 | chr12 | 31882596 | *AMN1* | chr12:31881630-31882461 | S_Shore | -0.16 | 1.73E-05 |
| cg16530981 | chr12 | 42679156 |  | chr12:42680607-42680889 | N_Shore | 0.19 | 0.004995423 |
| cg04101117 | chr12 | 42719598 | *PPHLN1* | chr12:42719761-42720119 | N_Shore | -0.10 | 0.000992457 |
| cg07020967 | chr12 | 44410498 | *TMEM117* |  | OpenSea | -0.12 | 0.007933857 |
| cg23192604 | chr12 | 46151535 | *ARID2* |  | OpenSea | -0.11 | 0.004503349 |
| cg09792000 | chr12 | 47225979 |  | chr12:47224912-47225664 | S_Shore | 0.13 | 0.008764375 |
| cg12075247 | chr12 | 47226029 |  | chr12:47224912-47225664 | S_Shore | 0.17 | 0.000113716 |
| cg12091498 | chr12 | 47226255 |  | chr12:47224912-47225664 | S_Shore | 0.13 | 0.001857213 |
| cg10502244 | chr12 | 48399295 | *COL2A1* | chr12:48399168-48399372 | Island | 0.14 | 0.000973309 |
| cg00985115 | chr12 | 48592818 |  | chr12:48591977-48592785 | S_Shore | -0.10 | 0.007972551 |
| cg10963793 | chr12 | 49111321 | *CCNT1* | chr12:49110055-49110881 | S_Shore | -0.10 | 0.003710902 |
| cg10924779 | chr12 | 49111395 | *CCNT1* | chr12:49110055-49110881 | S_Shore | -0.10 | 0.003063819 |
| cg23327859 | chr12 | 49111433 | *CCNT1* | chr12:49110055-49110881 | S_Shore | -0.12 | 0.00161058 |
| cg06712013 | chr12 | 49759545 | *SPATS2* | chr12:49760221-49761127 | N_Shore | -0.11 | 0.007080159 |
| cg21579107 | chr12 | 49783192 | *SPATS2* | chr12:49782965-49783193 | Island | 0.12 | 7.56E-05 |
| cg09239106 | chr12 | 49937997 | *KCNH3* |  | OpenSea | 0.14 | 1.32E-05 |
| cg10695105 | chr12 | 49943166 | *KCNH3* | chr12:49942786-49943435 | Island | -0.12 | 0.003034354 |
| cg22566355 | chr12 | 49943340 | *KCNH3* | chr12:49942786-49943435 | Island | -0.17 | 2.82E-08 |
| cg01043616 | chr12 | 50475035 | *ACCN2* | chr12:50474869-50475445 | Island | 0.11 | 0.003424462 |
| cg24210717 | chr12 | 50497827 | *GPD1* |  | OpenSea | 0.13 | 0.001067087 |
| cg06817772 | chr12 | 50678007 | *LIMA1* |  | OpenSea | -0.19 | 5.46E-05 |
| cg05655647 | chr12 | 51157023 | *ATF1* | chr12:51157498-51158842 | N_Shore | -0.19 | 0.006374431 |
| cg10377582 | chr12 | 51612794 | *POU6F1* | chr12:51610892-51611724 | S_Shore | -0.19 | 0.005945043 |
| cg02308712 | chr12 | 52598204 |  |  | OpenSea | 0.13 | 0.000252863 |
| cg22813430 | chr12 | 52626427 | *KRT7* | chr12:52626793-52627577 | N_Shore | 0.15 | 4.65E-05 |
| cg22958090 | chr12 | 52627438 | *KRT7* | chr12:52626793-52627577 | Island | 0.10 | 0.001231235 |
| cg12231340 | chr12 | 52685221 | *KRT81* | chr12:52684945-52685222 | Island | 0.11 | 0.000154918 |
| cg02491754 | chr12 | 53773040 | *SP1* | chr12:53774336-53774603 | N_Shore | -0.18 | 5.13E-07 |
| cg12097550 | chr12 | 54068942 | *ATP5G2* | chr12:54069625-54070177 | N_Shore | -0.11 | 0.000985444 |
| cg06868955 | chr12 | 54069197 | *ATP5G2* | chr12:54069625-54070177 | N_Shore | -0.19 | 6.08E-08 |
| cg22997177 | chr12 | 54070527 | *ATP5G2* | chr12:54071053-54071265 | N_Shore | -0.13 | 4.16E-09 |
| cg27479634 | chr12 | 54070545 | *ATP5G2* | chr12:54071053-54071265 | N_Shore | -0.12 | 6.17E-09 |
| cg10859192 | chr12 | 54071672 | *ATP5G2* | chr12:54069625-54070177 | S_Shore | -0.21 | 2.03E-05 |
| cg27597505 | chr12 | 54148217 |  | chr12:54144562-54145470 | S_Shelf | 0.13 | 0.004344106 |
| cg19641804 | chr12 | 54653065 | *CBX5* |  | OpenSea | -0.10 | 0.003756513 |
| cg22979422 | chr12 | 56694490 | *CS* | chr12:56693680-56694345 | S_Shore | -0.16 | 1.69E-08 |
| cg22775789 | chr12 | 56694541 | *CS* | chr12:56693680-56694345 | S_Shore | -0.20 | 1.44E-07 |
| cg16343272 | chr12 | 56694667 | *CS* | chr12:56693680-56694345 | S_Shore | -0.11 | 0.00016138 |
| cg13812230 | chr12 | 57915555 | *MBD6* | chr12:57916322-57916810 | N_Shore | -0.10 | 0.001312902 |
| cg19520710 | chr12 | 57915613 | *MBD6* | chr12:57916322-57916810 | N_Shore | -0.12 | 0.000629483 |
| cg14315430 | chr12 | 57915618 | *MBD6* | chr12:57916322-57916810 | N_Shore | -0.16 | 0.001133568 |
| cg09687907 | chr12 | 57915636 | *MBD6* | chr12:57916322-57916810 | N_Shore | -0.11 | 0.000629319 |
| cg06314111 | chr12 | 58119915 | *AGAP2* | chr12:58119909-58121551 | Island | -0.11 | 0.006398343 |
| cg00626466 | chr12 | 65153982 | *GNS* | chr12:65152869-65153489 | S_Shore | -0.11 | 0.007429005 |
| cg06533408 | chr12 | 69139618 | *SLC35E3* | chr12:69139815-69140206 | N_Shore | -0.11 | 0.002174711 |
| cg02837432 | chr12 | 72232889 | *TBC1D15* | chr12:72233323-72233635 | N_Shore | -0.14 | 7.89E-06 |
| cg04831505 | chr12 | 72233240 | *TBC1D15* | chr12:72233323-72233635 | N_Shore | -0.11 | 3.90E-09 |
| cg11565911 | chr12 | 72233249 | *TBC1D15* | chr12:72233323-72233635 | N_Shore | -0.13 | 2.29E-11 |
| cg03223580 | chr12 | 76742630 | *BBS10* | chr12:76741909-76742286 | S_Shore | -0.10 | 4.08E-09 |
| cg17824939 | chr12 | 88421705 | *C12orf50* |  | OpenSea | 0.16 | 0.007593835 |
| cg08743634 | chr12 | 88428894 | *C12orf29* | chr12:88429105-88429606 | N_Shore | -0.10 | 0.003719868 |
| cg21004565 | chr12 | 94070475 | *CRADD* | chr12:94071293-94071655 | N_Shore | -0.22 | 1.46E-05 |
| cg20922821 | chr12 | 94070578 | *CRADD* | chr12:94071293-94071655 | N_Shore | -0.28 | 2.29E-09 |
| cg13102120 | chr12 | 94070836 | *CRADD* | chr12:94071293-94071655 | N_Shore | -0.14 | 2.83E-05 |
| cg11207300 | chr12 | 95867190 | *METAP2* | chr12:95867584-95868019 | N_Shore | -0.14 | 0.000165349 |
| cg14565151 | chr12 | 95945384 | *USP44* | chr12:95941906-95942979 | S_Shelf | 0.11 | 0.00481677 |
| cg27246571 | chr12 | 96389588 | *HAL* | chr12:96389405-96389675 | Island | -0.12 | 0.00662417 |
| cg14077898 | chr12 | 100750695 | *SLC17A8* |  | OpenSea | 0.10 | 0.008527483 |
| cg20075675 | chr12 | 106458455 | *NUAK1* | chr12:106460673-106460892 | N_Shelf | 0.11 | 0.000428371 |
| cg14573817 | chr12 | 107348689 | *C12orf23* | chr12:107349152-107350089 | N_Shore | -0.15 | 2.48E-07 |
| cg04686545 | chr12 | 107348824 | *C12orf23* | chr12:107349152-107350089 | N_Shore | -0.14 | 1.54E-08 |
| cg10082647 | chr12 | 107348855 | *C12orf23* | chr12:107349152-107350089 | N_Shore | -0.19 | 0.001088477 |
| cg08817693 | chr12 | 107348944 | *C12orf23* | chr12:107349152-107350089 | N_Shore | -0.13 | 5.40E-05 |
| cg06834313 | chr12 | 108851556 |  |  | OpenSea | 0.11 | 0.000869008 |
| cg16624888 | chr12 | 109711141 |  |  | OpenSea | 0.18 | 0.000886291 |
| cg11367159 | chr12 | 110044531 |  |  | OpenSea | 0.17 | 0.0026026 |
| cg10504392 | chr12 | 110044639 |  |  | OpenSea | 0.18 | 0.000175794 |
| cg00851732 | chr12 | 113229793 | *RPH3A* |  | OpenSea | -0.11 | 0.004026885 |
| cg12590521 | chr12 | 121678474 | *CAMKK2* |  | OpenSea | 0.11 | 0.002656616 |
| cg15695155 | chr12 | 121973871 | *KDM2B* | chr12:121975028-121976140 | N_Shore | -0.16 | 0.009692716 |
| cg10378667 | chr12 | 122326303 | *PSMD9* | chr12:122326482-122327044 | N_Shore | -0.10 | 3.16E-07 |
| cg20670946 | chr12 | 122502170 |  |  | OpenSea | -0.16 | 4.11E-05 |
| cg12389043 | chr12 | 124429228 | *CCDC92* |  | OpenSea | -0.16 | 0.003427067 |
| cg26853536 | chr12 | 125399964 | *UBC* | chr12:125398558-125399899 | S_Shore | -0.13 | 0.00021404 |
| cg00254306 | chr12 | 126346041 |  | chr12:126345899-126346531 | Island | -0.10 | 0.003754144 |
| cg10857167 | chr12 | 126346276 |  | chr12:126345899-126346531 | Island | -0.10 | 0.004309868 |
| cg04325312 | chr12 | 126346509 |  | chr12:126345899-126346531 | Island | -0.11 | 0.00103205 |
| cg25825627 | chr12 | 127631198 |  | chr12:127630511-127631199 | Island | -0.25 | 2.25E-06 |
| cg10643271 | chr12 | 129309330 | *SLC15A4* | chr12:129307947-129308724 | S_Shore | -0.11 | 0.001505002 |
| cg23081604 | chr12 | 130824224 | *PIWIL1* | chr12:130822360-130822696 | S_Shore | 0.14 | 0.00040668 |
| cg10832093 | chr12 | 131303194 | *STX2* | chr12:131303093-131303836 | Island | -0.13 | 0.004116936 |
| cg11011512 | chr12 | 131303247 | *STX2* | chr12:131303093-131303836 | Island | -0.11 | 0.007281319 |
| cg20535781 | chr12 | 133465013 | *CHFR* | chr12:133463807-133464858 | S_Shore | -0.11 | 0.000663699 |
| cg25987452 | chr13 | 20531342 | *ZMYM2* | chr13:20531548-20533630 | N_Shore | -0.21 | 1.09E-05 |
| cg01841471 | chr13 | 20879896 |  | chr13:20875518-20876214 | S_Shelf | 0.11 | 1.65E-07 |
| cg11203293 | chr13 | 25777762 |  |  | OpenSea | 0.15 | 0.001245959 |
| cg10776186 | chr13 | 25875020 | *NUPL1* | chr13:25874994-25876200 | Island | -0.18 | 0.000388974 |
| cg21535947 | chr13 | 25875133 | *NUPL1* | chr13:25874994-25876200 | Island | -0.19 | 1.61E-06 |
| cg08532057 | chr13 | 25875436 | *NUPL1* | chr13:25874994-25876200 | Island | -0.15 | 2.40E-05 |
| cg25877436 | chr13 | 27998191 | *GTF3A* | chr13:27998586-27999178 | N_Shore | -0.14 | 4.09E-05 |
| cg20147645 | chr13 | 28023563 | *MTIF3* | chr13:28024165-28025278 | N_Shore | -0.14 | 1.43E-06 |
| cg08003353 | chr13 | 31407120 |  |  | OpenSea | 0.11 | 0.002011673 |
| cg20743905 | chr13 | 34116177 |  | chr13:34116557-34117140 | N_Shore | -0.15 | 0.000393993 |
| cg01757116 | chr13 | 34118017 |  | chr13:34116557-34117140 | S_Shore | -0.18 | 4.61E-07 |
| cg06927812 | chr13 | 41718870 |  |  | OpenSea | -0.15 | 0.001254169 |
| cg21800400 | chr13 | 42845622 | *AKAP11* | chr13:42845853-42846807 | N_Shore | -0.13 | 2.69E-06 |
| cg27021512 | chr13 | 47371635 | *ESD* | chr13:47370763-47371484 | S_Shore | -0.16 | 4.00E-07 |
| cg15894389 | chr13 | 47470857 | *HTR2A* |  | OpenSea | -0.13 | 0.007121711 |
| cg20733077 | chr13 | 50700845 | *DLEU2* | chr13:50697984-50702286 | Island | -0.32 | 1.74E-09 |
| cg01404873 | chr13 | 50701050 | *DLEU2* | chr13:50697984-50702286 | Island | -0.28 | 3.16E-06 |
| cg13204699 | chr13 | 50703841 |  | chr13:50703685-50705025 | Island | 0.11 | 0.001633833 |
| cg19906545 | chr13 | 51416639 | *DLEU7* | chr13:51417371-51418149 | N_Shore | -0.13 | 0.001478689 |
| cg08274637 | chr13 | 51417923 | *DLEU7* | chr13:51417371-51418149 | Island | -0.22 | 3.70E-05 |
| cg13846270 | chr13 | 51417929 | *DLEU7* | chr13:51417371-51418149 | Island | -0.13 | 0.004414181 |
| cg27051129 | chr13 | 51417977 | *DLEU7* | chr13:51417371-51418149 | Island | -0.17 | 0.002345756 |
| cg05965387 | chr13 | 51418053 | *DLEU7* | chr13:51417371-51418149 | Island | -0.12 | 0.003121562 |
| cg17288288 | chr13 | 51418092 | *DLEU7* | chr13:51417371-51418149 | Island | -0.14 | 0.000111501 |
| cg26960333 | chr13 | 92051705 | *GPC5* | chr13:92051153-92051716 | Island | 0.12 | 0.00315869 |
| cg20647118 | chr13 | 92051786 | *GPC5* | chr13:92051153-92051716 | S_Shore | 0.16 | 0.001012737 |
| cg09582042 | chr13 | 95253676 | *GPR180* | chr13:95253814-95254569 | N_Shore | -0.11 | 0.002911834 |
| cg03485172 | chr13 | 95655579 |  | chr13:95655108-95655517 | S_Shore | -0.11 | 0.000297785 |
| cg04015777 | chr13 | 103426481 | *C13orf27* | chr13:103425298-103426887 | Island | -0.15 | 5.50E-08 |
| cg27260867 | chr13 | 112984840 |  | chr13:112984966-112985733 | N_Shore | 0.16 | 0.007201296 |
| cg19916129 | chr13 | 113242878 | *TUBGCP3* | chr13:113241711-113241925 | S_Shore | -0.36 | 2.40E-11 |
| cg08602346 | chr13 | 113242997 | *TUBGCP3* | chr13:113241711-113241925 | S_Shore | -0.34 | 7.67E-13 |
| cg00598009 | chr13 | 113243141 | *TUBGCP3* | chr13:113241711-113241925 | S_Shore | -0.28 | 1.95E-09 |
| cg16169675 | chr13 | 113594722 |  | chr13:113597553-113598303 | N_Shelf | -0.15 | 0.007985714 |
| cg14904725 | chr13 | 113777045 | *F10* |  | OpenSea | 0.10 | 0.003432207 |
| cg16746221 | chr14 | 20666088 | *OR11G2* |  | OpenSea | -0.19 | 0.001753765 |
| cg22872033 | chr14 | 21725703 | *HNRNPC* |  | OpenSea | -0.18 | 0.000739858 |
| cg13182010 | chr14 | 23235291 | *OXA1L* | chr14:23235774-23236578 | N_Shore | -0.26 | 7.09E-08 |
| cg04861271 | chr14 | 23789531 | *PABPN1* | chr14:23790212-23790659 | N_Shore | -0.17 | 0.000308854 |
| cg07606234 | chr14 | 24551147 | *NRL* | chr14:24550317-24551148 | Island | -0.16 | 3.10E-07 |
| cg09399371 | chr14 | 24702584 | *GMPR2* | chr14:24701646-24701992 | S_Shore | -0.15 | 2.20E-08 |
| cg21886367 | chr14 | 24780825 | *LTB4R* | chr14:24779874-24780932 | Island | -0.16 | 0.000709894 |
| cg12853742 | chr14 | 24780890 | *LTB4R* | chr14:24779874-24780932 | Island | -0.30 | 3.66E-10 |
| cg13840968 | chr14 | 24780926 | *LTB4R* | chr14:24779874-24780932 | Island | -0.17 | 1.49E-05 |
| cg01214012 | chr14 | 30585046 |  |  | OpenSea | -0.12 | 0.009371543 |
| cg03839794 | chr14 | 34269541 | *NPAS3* | chr14:34268946-34270438 | Island | 0.11 | 0.003576507 |
| cg15089111 | chr14 | 34270113 | *NPAS3* | chr14:34268946-34270438 | Island | 0.12 | 0.001470796 |
| cg01993818 | chr14 | 34270437 | *NPAS3* | chr14:34268946-34270438 | Island | 0.19 | 0.004054559 |
| cg16874095 | chr14 | 34529121 |  | chr14:34529120-34529959 | Island | -0.16 | 1.52E-06 |
| cg12112434 | chr14 | 45722987 | *C14orf106* | chr14:45722148-45722802 | S_Shore | -0.14 | 0.000221522 |
| cg18174881 | chr14 | 45723353 | *C14orf106* | chr14:45722148-45722802 | S_Shore | -0.51 | 3.56E-13 |
| cg05225684 | chr14 | 53257343 | *GNPNAT1* | chr14:53257664-53258400 | N_Shore | -0.12 | 0.00161058 |
| cg00917437 | chr14 | 55879046 | *KIAA0831* | chr14:55878302-55878681 | S_Shore | -0.14 | 1.94E-08 |
| cg04752257 | chr14 | 58862417 | *TOMM20L* | chr14:58862541-58863209 | N_Shore | -0.12 | 6.56E-06 |
| cg04728863 | chr14 | 58863362 | *TOMM20L* | chr14:58862541-58863209 | S_Shore | -0.12 | 0.000779171 |
| cg15853715 | chr14 | 60955348 |  | chr14:60951976-60952946 | S_Shelf | -0.14 | 0.003623537 |
| cg12120359 | chr14 | 65006041 | *HSPA2* | chr14:65006874-65009197 | N_Shore | -0.13 | 5.44E-05 |
| cg26421140 | chr14 | 65006053 | *HSPA2* | chr14:65006874-65009197 | N_Shore | -0.11 | 8.91E-06 |
| cg23373640 | chr14 | 65696480 |  |  | OpenSea | -0.21 | 1.44E-08 |
| cg15999311 | chr14 | 65749247 |  | chr14:65746329-65746972 | S_Shelf | -0.13 | 0.003192591 |
| cg12813394 | chr14 | 69095057 |  | chr14:69095051-69095407 | Island | -0.20 | 0.007297261 |
| cg00989365 | chr14 | 70014548 |  | chr14:70014543-70014993 | Island | 0.11 | 0.000890272 |
| cg19937979 | chr14 | 70039915 |  | chr14:70038108-70040302 | Island | 0.14 | 0.000238922 |
| cg07004386 | chr14 | 70040391 |  | chr14:70041213-70041662 | N_Shore | 0.18 | 0.001152168 |
| cg20262330 | chr14 | 70826997 | *COX16* | chr14:70826255-70826580 | S_Shore | -0.13 | 0.000799189 |
| cg13244312 | chr14 | 71109707 | *TTC9* | chr14:71108008-71109332 | S_Shore | 0.12 | 0.007049506 |
| cg18364502 | chr14 | 73524688 | *RBM25* | chr14:73524989-73525752 | N_Shore | -0.16 | 4.16E-06 |
| cg08156120 | chr14 | 74111075 | *DNAL1* | chr14:74111560-74111766 | N_Shore | -0.14 | 0.000286204 |
| cg01624173 | chr14 | 75981868 |  |  | OpenSea | -0.16 | 0.003632235 |
| cg19628934 | chr14 | 77499391 |  | chr14:77499154-77499502 | Island | -0.11 | 0.000206754 |
| cg20068496 | chr14 | 78108822 |  | chr14:78108170-78108662 | S_Shore | -0.12 | 0.004661021 |
| cg04293460 | chr14 | 81688339 | *GTF2A1* | chr14:81686385-81687885 | S_Shore | -0.28 | 2.18E-09 |
| cg23934295 | chr14 | 85995655 | *FLRT2* | chr14:85996494-85996958 | N_Shore | 0.13 | 0.000779268 |
| cg10406690 | chr14 | 85995726 | *FLRT2* | chr14:85996494-85996958 | N_Shore | 0.13 | 6.46E-06 |
| cg24058132 | chr14 | 88459866 | *GALC* | chr14:88459118-88459689 | S_Shore | -0.12 | 4.15E-08 |
| cg19998073 | chr14 | 89078443 | *ZC3H14* |  | OpenSea | -0.12 | 0.000935317 |
| cg19194924 | chr14 | 90168307 |  | chr14:90167782-90168352 | Island | -0.10 | 2.51E-07 |
| cg06607384 | chr14 | 91579859 | *C14orf159* |  | OpenSea | -0.13 | 5.05E-07 |
| cg06907680 | chr14 | 91580129 | *C14orf159* |  | OpenSea | -0.19 | 4.74E-10 |
| cg05084793 | chr14 | 91977571 | *SMEK1* | chr14:91975670-91977326 | S_Shore | -0.10 | 0.000155575 |
| cg25293251 | chr14 | 93260171 | *GOLGA5* | chr14:93260307-93260924 | N_Shore | -0.12 | 7.43E-06 |
| cg00748938 | chr14 | 94641781 | *PPP4R4* | chr14:94640366-94641648 | S_Shore | -0.12 | 4.66E-05 |
| cg05293490 | chr14 | 95156546 |  |  | OpenSea | 0.12 | 0.005081711 |
| cg18678107 | chr14 | 100130465 | *HHIPL1* | chr14:100125671-100126694 | S_Shelf | 0.14 | 0.001211716 |
| cg00232105 | chr14 | 100752338 |  | chr14:100751672-100752339 | Island | -0.11 | 7.89E-06 |
| cg02854229 | chr14 | 101962994 |  |  | OpenSea | 0.16 | 8.43E-05 |
| cg12123733 | chr14 | 102025815 |  | chr14:102025989-102031567 | N_Shore | 0.11 | 0.000156731 |
| cg18329187 | chr14 | 103989711 | *CKB* | chr14:103987063-103989796 | Island | -0.13 | 0.005712929 |
| cg01121022 | chr14 | 104338788 |  |  | OpenSea | -0.11 | 1.43E-05 |
| cg20870298 | chr14 | 104569529 | *ASPG* | chr14:104569499-104569780 | Island | 0.10 | 0.000826313 |
| cg26076119 | chr14 | 104666047 |  |  | OpenSea | -0.11 | 0.001116789 |
| cg14543641 | chr14 | 104940939 |  | chr14:104940228-104940466 | S_Shore | 0.10 | 0.000859185 |
| cg10909152 | chr14 | 105218528 | *SIVA1* | chr14:105218366-105218620 | Island | -0.12 | 0.002375611 |
| cg19495714 | chr14 | 105218551 | *SIVA1* | chr14:105218366-105218620 | Island | -0.19 | 0.002527437 |
| cg05881762 | chr15 | 25684849 | *UBE3A* | chr15:25683348-25684415 | S_Shore | -0.14 | 2.57E-07 |
| cg17234513 | chr15 | 27087554 |  | chr15:27083992-27084222 | S_Shelf | -0.13 | 0.005700101 |
| cg09214243 | chr15 | 29968124 |  | chr15:29967209-29967621 | S_Shore | 0.24 | 0.000618202 |
| cg12414681 | chr15 | 29968195 |  | chr15:29967209-29967621 | S_Shore | 0.17 | 2.55E-05 |
| cg27305460 | chr15 | 29969096 |  | chr15:29967209-29967621 | S_Shore | 0.13 | 0.004199337 |
| cg05180717 | chr15 | 34516488 | *TMEM85* |  | OpenSea | -0.20 | 0.003151036 |
| cg14847483 | chr15 | 34516640 | *TMEM85* |  | OpenSea | -0.15 | 0.005440451 |
| cg23120601 | chr15 | 40583227 | *PLCB2* | chr15:40583093-40583526 | Island | -0.24 | 0.001637181 |
| cg09233429 | chr15 | 40583241 | *PLCB2* | chr15:40583093-40583526 | Island | -0.22 | 0.000575319 |
| cg15852446 | chr15 | 40583422 | *PLCB2* | chr15:40583093-40583526 | Island | -0.18 | 0.000991917 |
| cg13897914 | chr15 | 41695294 | *NDUFAF1* | chr15:41694493-41694756 | S_Shore | -0.12 | 0.000135043 |
| cg17082938 | chr15 | 44828179 | *EIF3J* | chr15:44828637-44829949 | N_Shore | -0.14 | 5.63E-06 |
| cg04036182 | chr15 | 45458818 |  | chr15:45458801-45459580 | Island | -0.14 | 6.81E-06 |
| cg07427772 | chr15 | 50412376 | *ATP8B4* |  | OpenSea | -0.12 | 0.006555737 |
| cg25541928 | chr15 | 51973199 | *SCG3* | chr15:51973533-51973838 | N_Shore | -0.11 | 0.004978978 |
| cg12957265 | chr15 | 57026014 | *ZNF280D* | chr15:57025347-57026150 | Island | -0.13 | 0.000269745 |
| cg09190051 | chr15 | 57026056 | *ZNF280D* | chr15:57025347-57026150 | Island | -0.13 | 0.001845647 |
| cg09782034 | chr15 | 61522112 | *RORA* | chr15:61520423-61521716 | S_Shore | 0.11 | 0.000665725 |
| cg23845646 | chr15 | 65116194 | *PIF1* | chr15:65116013-65116567 | Island | -0.11 | 0.007453045 |
| cg27192248 | chr15 | 65285669 |  | chr15:65281928-65282375 | S_Shelf | -0.18 | 0.003432207 |
| cg22706883 | chr15 | 67814880 | *C15orf61* | chr15:67813370-67814181 | S_Shore | -0.12 | 0.008162689 |
| cg05338167 | chr15 | 68498251 | *CALML4* |  | OpenSea | -0.10 | 0.008486082 |
| cg22952142 | chr15 | 68549178 |  |  | OpenSea | -0.29 | 4.16E-09 |
| cg04774597 | chr15 | 69744390 | *RPLP1* | chr15:69745049-69745746 | N_Shore | -0.18 | 1.39E-08 |
| cg26218577 | chr15 | 69744466 | *RPLP1* | chr15:69745049-69745746 | N_Shore | -0.26 | 7.35E-09 |
| cg07513768 | chr15 | 69744528 | *RPLP1* | chr15:69745049-69745746 | N_Shore | -0.14 | 1.75E-07 |
| cg00469015 | chr15 | 69744684 | *RPLP1* | chr15:69745049-69745746 | N_Shore | -0.17 | 1.54E-08 |
| cg11437810 | chr15 | 69744829 | *RPLP1* | chr15:69745049-69745746 | N_Shore | -0.13 | 4.65E-08 |
| cg10716823 | chr15 | 69744850 | *RPLP1* | chr15:69745049-69745746 | N_Shore | -0.11 | 1.65E-07 |
| cg07469063 | chr15 | 69755704 |  |  | OpenSea | -0.12 | 0.003480589 |
| cg18583931 | chr15 | 71378283 |  |  | OpenSea | 0.10 | 7.61E-05 |
| cg20801110 | chr15 | 75136007 | *ULK3* | chr15:75135186-75135905 | S_Shore | -0.20 | 1.82E-07 |
| cg13055199 | chr15 | 75136278 | *ULK3* | chr15:75135186-75135905 | S_Shore | -0.17 | 6.07E-06 |
| cg15611336 | chr15 | 75248496 | *RPP25* | chr15:75248277-75249922 | Island | -0.11 | 0.000568873 |
| cg20630655 | chr15 | 75918700 | *SNUPN* | chr15:75917646-75918212 | S_Shore | -0.15 | 1.20E-06 |
| cg23097139 | chr15 | 75918757 | *SNUPN* | chr15:75917646-75918212 | S_Shore | -0.13 | 3.72E-06 |
| cg16587909 | chr15 | 76031591 | *DNM1P35* | chr15:76030564-76031664 | Island | -0.18 | 1.46E-05 |
| cg12226028 | chr15 | 76484473 | *C15orf27* | chr15:76484093-76484580 | Island | 0.13 | 0.000711186 |
| cg24795825 | chr15 | 79164541 | *MORF4L1* | chr15:79165102-79166057 | N_Shore | -0.28 | 0.00070699 |
| cg03589001 | chr15 | 79164714 | *MORF4L1* | chr15:79165102-79166057 | N_Shore | -0.16 | 0.000452775 |
| cg08084502 | chr15 | 83240612 | *CPEB1* |  | OpenSea | 0.13 | 8.69E-05 |
| cg00202460 | chr15 | 83680873 | *C15orf40* | chr15:83680007-83680532 | S_Shore | -0.13 | 3.63E-09 |
| cg18407136 | chr15 | 83681092 | *C15orf40* | chr15:83680007-83680532 | S_Shore | -0.26 | 1.30E-08 |
| cg10646368 | chr15 | 83974335 |  |  | OpenSea | 0.12 | 2.03E-08 |
| cg02030270 | chr15 | 84215725 | *SH3GL3* |  | OpenSea | -0.11 | 0.003654025 |
| cg08961287 | chr15 | 89919993 | *LOC254559* | chr15:89920793-89922768 | N_Shore | 0.14 | 6.84E-07 |
| cg17301216 | chr15 | 89920348 | *LOC254559* | chr15:89920793-89922768 | N_Shore | 0.38 | 1.12E-14 |
| cg27407147 | chr15 | 89920824 | *LOC254559* | chr15:89920793-89922768 | Island | 0.37 | 5.34E-14 |
| cg21751684 | chr15 | 89920887 | *LOC254559* | chr15:89920793-89922768 | Island | 0.28 | 3.32E-08 |
| cg03766449 | chr15 | 89920918 | *LOC254559* | chr15:89920793-89922768 | Island | 0.23 | 2.75E-12 |
| cg20738500 | chr15 | 89921156 | *LOC254559* | chr15:89920793-89922768 | Island | 0.25 | 8.90E-10 |
| cg00481644 | chr15 | 89921158 | *LOC254559* | chr15:89920793-89922768 | Island | 0.24 | 5.72E-09 |
| cg12598235 | chr15 | 89921182 | *LOC254559* | chr15:89920793-89922768 | Island | 0.31 | 1.58E-12 |
| cg01317586 | chr15 | 89921236 | *LOC254559* | chr15:89920793-89922768 | Island | 0.16 | 0.005872268 |
| cg21029612 | chr15 | 89921258 | *LOC254559* | chr15:89920793-89922768 | Island | 0.15 | 5.20E-06 |
| cg09169617 | chr15 | 89921672 | *LOC254559* | chr15:89920793-89922768 | Island | 0.14 | 8.31E-07 |
| cg08175536 | chr15 | 89927750 | *LOC254559* |  | OpenSea | 0.12 | 2.46E-06 |
| cg20467658 | chr15 | 89933347 | *LOC254559* |  | OpenSea | 0.20 | 2.34E-07 |
| cg27355653 | chr15 | 89940260 | *LOC254559* | chr15:89942592-89943853 | N_Shelf | 0.25 | 2.95E-07 |
| cg26645709 | chr15 | 89987792 |  |  | OpenSea | 0.11 | 0.000670236 |
| cg14295110 | chr15 | 92395641 | *SLCO3A1* | chr15:92396013-92397682 | N_Shore | -0.11 | 3.01E-05 |
| cg20651758 | chr15 | 93425245 |  | chr15:93426151-93426751 | N_Shore | -0.14 | 2.65E-05 |
| cg21848673 | chr15 | 93876581 |  |  | OpenSea | 0.10 | 0.0071835 |
| cg19459332 | chr15 | 98196247 |  | chr15:98196021-98196391 | Island | 0.14 | 0.009481219 |
| cg20891771 | chr15 | 99143542 |  |  | OpenSea | -0.11 | 0.000503471 |
| cg21819984 | chr15 | 101084507 | *LASS3* | chr15:101084428-101085178 | Island | 0.16 | 0.009604753 |
| cg26173375 | chr15 | 101084509 | *LASS3* | chr15:101084428-101085178 | Island | 0.16 | 0.006291004 |
| cg13202221 | chr15 | 102193228 | *TM2D3* | chr15:102192165-102193345 | Island | -0.17 | 2.09E-08 |
| cg06505619 | chr16 | 698072 | *WDR90* | chr16:698826-700244 | N_Shore | -0.23 | 0.000156656 |
| cg01120509 | chr16 | 698230 | *WDR90* | chr16:698826-700244 | N_Shore | -0.15 | 1.59E-05 |
| cg09465703 | chr16 | 733842 | *JMJD8* | chr16:729438-735815 | Island | -0.12 | 0.001936105 |
| cg27436995 | chr16 | 743998 | *FBXL16* | chr16:743924-745943 | Island | 0.20 | 0.000990808 |
| cg05542681 | chr16 | 744328 | *FBXL16* | chr16:743924-745943 | Island | 0.12 | 0.000606537 |
| cg05724492 | chr16 | 979662 | *LMF1* | chr16:979422-979663 | Island | -0.16 | 0.004715174 |
| cg16748643 | chr16 | 1336696 |  |  | OpenSea | 0.14 | 0.003670635 |
| cg26751513 | chr16 | 1542910 | *TELO2* | chr16:1543098-1544426 | N_Shore | -0.17 | 4.64E-09 |
| cg06602086 | chr16 | 1583883 | *IFT140* | chr16:1583809-1584641 | Island | -0.22 | 0.00173491 |
| cg00463982 | chr16 | 1583984 | *IFT140* | chr16:1583809-1584641 | Island | -0.18 | 0.002712047 |
| cg10465839 | chr16 | 1584050 | *IFT140* | chr16:1583809-1584641 | Island | -0.12 | 0.000780294 |
| cg08296037 | chr16 | 1584118 | *IFT140* | chr16:1583809-1584641 | Island | -0.23 | 1.49E-06 |
| cg06565913 | chr16 | 1584452 | *IFT140* | chr16:1583809-1584641 | Island | -0.17 | 0.000246526 |
| cg07639376 | chr16 | 1584516 | *IFT140* | chr16:1583809-1584641 | Island | -0.22 | 0.008277234 |
| cg06736444 | chr16 | 2801793 | *LOC100128788* | chr16:2801913-2803409 | N_Shore | -0.22 | 2.34E-07 |
| cg10575367 | chr16 | 2801914 | *LOC100128788* | chr16:2801913-2803409 | Island | -0.24 | 5.09E-08 |
| cg03507326 | chr16 | 2801952 | *LOC100128788* | chr16:2801913-2803409 | Island | -0.47 | 3.18E-10 |
| cg05485462 | chr16 | 3062349 | *CLDN9* | chr16:3063679-3063981 | N_Shore | 0.14 | 3.52E-06 |
| cg10282491 | chr16 | 3062368 | *CLDN9* | chr16:3063679-3063981 | N_Shore | 0.13 | 0.000246526 |
| cg09674170 | chr16 | 3062382 | *CLDN9* | chr16:3063679-3063981 | N_Shore | 0.14 | 3.99E-05 |
| cg08199758 | chr16 | 3062426 | *CLDN9* | chr16:3063679-3063981 | N_Shore | 0.11 | 0.000208407 |
| cg27009812 | chr16 | 3062597 | *CLDN9* | chr16:3063679-3063981 | N_Shore | 0.18 | 1.42E-05 |
| cg06117341 | chr16 | 3062653 | *CLDN9* | chr16:3063679-3063981 | N_Shore | 0.14 | 0.000193744 |
| cg04715503 | chr16 | 3062795 | *CLDN9* | chr16:3063679-3063981 | N_Shore | 0.30 | 4.93E-06 |
| cg06489965 | chr16 | 3062975 | *CLDN9* | chr16:3063679-3063981 | N_Shore | 0.13 | 0.000170077 |
| cg08842287 | chr16 | 3747018 | *TRAP1* |  | OpenSea | -0.16 | 0.008264903 |
| cg12031863 | chr16 | 4587854 | *C16orf5* | chr16:4587836-4588890 | Island | -0.14 | 0.002523317 |
| cg09549813 | chr16 | 4587862 | *C16orf5* | chr16:4587836-4588890 | Island | -0.15 | 0.001036983 |
| cg09712682 | chr16 | 10647275 | *EMP2* |  | OpenSea | -0.14 | 0.003268307 |
| cg07571928 | chr16 | 22020630 | *C16orf52* | chr16:22018987-22020057 | S_Shore | 0.13 | 0.005966932 |
| cg02747254 | chr16 | 29938183 | *KCTD13* | chr16:29937038-29937761 | S_Shore | -0.13 | 0.002789071 |
| cg03890691 | chr16 | 30023615 | *DOC2A* | chr16:30022651-30023007 | S_Shore | 0.13 | 0.001188113 |
| cg00583733 | chr16 | 30075102 | *ALDOA* | chr16:30076310-30077872 | N_Shore | -0.12 | 5.11E-06 |
| cg03991297 | chr16 | 30429837 | *ZNF771* | chr16:30428870-30429799 | S_Shore | -0.13 | 4.31E-05 |
| cg03776506 | chr16 | 30581384 | *ZNF688* | chr16:30581227-30581534 | Island | -0.10 | 0.00474045 |
| cg16591159 | chr16 | 31487813 | *TGFB1I1* | chr16:31487152-31488967 | Island | -0.14 | 0.004448968 |
| cg05079544 | chr16 | 48644408 | *N4BP1* | chr16:48643589-48643860 | S_Shore | -0.12 | 0.000246526 |
| cg27268835 | chr16 | 51475605 |  |  | OpenSea | -0.11 | 0.002911834 |
| cg00253658 | chr16 | 54210496 |  |  | OpenSea | 0.12 | 0.003693685 |
| cg03232842 | chr16 | 56995201 | *CETP* |  | OpenSea | -0.12 | 0.004496812 |
| cg27206026 | chr16 | 57907171 |  |  | OpenSea | 0.10 | 0.005945292 |
| cg13500388 | chr16 | 67062135 | *CBFB* | chr16:67062613-67064012 | N_Shore | -0.11 | 4.94E-05 |
| cg00589006 | chr16 | 70415864 | *ST3GAL2* | chr16:70414429-70415865 | Island | -0.11 | 2.59E-07 |
| cg08961664 | chr16 | 72821141 | *ZFHX3* | chr16:72821140-72821592 | Island | -0.14 | 0.000496276 |
| cg02282640 | chr16 | 81743587 | *CMIP* |  | OpenSea | -0.12 | 0.006002134 |
| cg00411413 | chr16 | 83171299 | *CDH13* |  | OpenSea | -0.16 | 0.002132089 |
| cg06652011 | chr16 | 83968316 |  |  | OpenSea | -0.23 | 0.00091516 |
| cg01684248 | chr16 | 86536239 |  | chr16:86539118-86539486 | N_Shelf | -0.11 | 0.007783049 |
| cg06301726 | chr16 | 86589283 | *MTHFSD* | chr16:86588435-86589513 | Island | -0.13 | 0.001408912 |
| cg05594286 | chr16 | 86840467 |  |  | OpenSea | 0.14 | 0.004771529 |
| cg01329973 | chr16 | 86912065 |  | chr16:86912403-86912670 | N_Shore | 0.10 | 2.76E-06 |
| cg00104167 | chr16 | 86953195 |  |  | OpenSea | 0.11 | 0.000230521 |
| cg02830202 | chr16 | 86953383 |  |  | OpenSea | 0.13 | 0.00122051 |
| cg09610767 | chr16 | 86960025 |  |  | OpenSea | 0.11 | 0.001147122 |
| cg07304536 | chr16 | 86987635 |  |  | OpenSea | 0.19 | 9.95E-07 |
| cg06286618 | chr16 | 86987702 |  |  | OpenSea | 0.15 | 5.19E-06 |
| cg05324407 | chr16 | 86987828 |  |  | OpenSea | 0.18 | 3.28E-06 |
| cg08196561 | chr16 | 87525539 | *ZCCHC14* |  | OpenSea | -0.19 | 0.00241935 |
| cg05142677 | chr16 | 88601446 | *ZFPM1* | chr16:88599610-88601530 | Island | -0.18 | 1.31E-05 |
| cg00842595 | chr17 | 263193 | *C17orf97* | chr17:263083-263412 | Island | -0.21 | 0.000167824 |
| cg08489349 | chr17 | 656181 | *ELP2P* | chr17:655026-656707 | Island | -0.16 | 0.000616818 |
| cg26682335 | chr17 | 945834 | *ABR* |  | OpenSea | 0.11 | 0.000739858 |
| cg22806229 | chr17 | 1945954 | *OVCA2* | chr17:1944947-1945621 | S_Shore | -0.10 | 0.000932834 |
| cg00668150 | chr17 | 2615720 | *KIAA0664* | chr17:2614320-2615630 | S_Shore | -0.25 | 2.73E-07 |
| cg04794430 | chr17 | 3290071 |  | chr17:3289362-3290072 | Island | -0.11 | 0.003968996 |
| cg16810621 | chr17 | 3290164 |  | chr17:3289362-3290072 | S_Shore | -0.22 | 0.000238581 |
| cg09580393 | chr17 | 4869866 | *SPAG7* | chr17:4870189-4871710 | N_Shore | -0.11 | 0.000118738 |
| cg27507261 | chr17 | 5403053 | *LOC728392* | chr17:5403131-5404602 | N_Shore | -0.10 | 0.003756222 |
| cg16880424 | chr17 | 7287922 | *TNK1* | chr17:7287198-7287940 | Island | -0.12 | 3.44E-06 |
| cg20116579 | chr17 | 7288001 | *TNK1* | chr17:7287198-7287940 | S_Shore | -0.12 | 4.39E-05 |
| cg09711113 | chr17 | 7311964 | *NLGN2* | chr17:7311672-7312063 | Island | -0.20 | 0.001585677 |
| cg10369169 | chr17 | 7312041 | *NLGN2* | chr17:7311672-7312063 | Island | -0.11 | 9.21E-06 |
| cg08870143 | chr17 | 7312081 | *NLGN2* | chr17:7311672-7312063 | S_Shore | -0.23 | 4.93E-06 |
| cg17887478 | chr17 | 7486551 | *MPDU1* | chr17:7486285-7487431 | Island | -0.38 | 1.23E-20 |
| cg11874331 | chr17 | 7486562 | *MPDU1* | chr17:7486285-7487431 | Island | -0.32 | 8.40E-18 |
| cg05086567 | chr17 | 7486615 | *MPDU1* | chr17:7486285-7487431 | Island | -0.40 | 1.74E-11 |
| cg06931941 | chr17 | 7486770 | *MPDU1* | chr17:7486285-7487431 | Island | -0.38 | 1.36E-20 |
| cg09663193 | chr17 | 7486821 | *MPDU1* | chr17:7486285-7487431 | Island | -0.22 | 6.14E-22 |
| cg13546736 | chr17 | 7486858 | *MPDU1* | chr17:7486285-7487431 | Island | -0.22 | 1.17E-18 |
| cg07451297 | chr17 | 7486874 | *MPDU1* | chr17:7486285-7487431 | Island | -0.14 | 2.33E-16 |
| cg21969795 | chr17 | 7759140 | *TMEM88* | chr17:7760281-7761680 | N_Shore | -0.12 | 0.005937212 |
| cg04615859 | chr17 | 7762123 | *CYB5D1* | chr17:7760281-7761680 | S_Shore | -0.14 | 0.000970105 |
| cg05465916 | chr17 | 7819762 | *LOC284023* | chr17:7818938-7819763 | Island | -0.16 | 1.26E-10 |
| cg25518824 | chr17 | 7819783 | *LOC284023* | chr17:7818938-7819763 | S_Shore | -0.10 | 7.06E-07 |
| cg13021301 | chr17 | 7836533 | *TRAPPC1* | chr17:7835171-7835569 | S_Shore | 0.12 | 0.000876967 |
| cg01667702 | chr17 | 7836604 | *TRAPPC1* | chr17:7835171-7835569 | S_Shore | 0.12 | 0.000863515 |
| cg01837574 | chr17 | 7836610 | *TRAPPC1* | chr17:7835171-7835569 | S_Shore | 0.11 | 0.005082528 |
| cg06319579 | chr17 | 8127196 | *C17orf44* | chr17:8126224-8126486 | S_Shore | -0.17 | 1.05E-12 |
| cg21722785 | chr17 | 8127367 | *C17orf44* | chr17:8126224-8126486 | S_Shore | -0.23 | 7.19E-10 |
| cg25228625 | chr17 | 8127373 | *C17orf44* | chr17:8126224-8126486 | S_Shore | -0.17 | 1.11E-06 |
| cg08125503 | chr17 | 16119344 | *NCOR1* | chr17:16118275-16119510 | Island | -0.12 | 2.67E-06 |
| cg22291359 | chr17 | 16119456 | *NCOR1* | chr17:16118275-16119510 | Island | -0.13 | 6.74E-07 |
| cg12778228 | chr17 | 16341601 | *NCRNA00188* | chr17:16342504-16342897 | N_Shore | -0.14 | 0.005398131 |
| cg07138452 | chr17 | 17141020 | *FLCN* | chr17:17140117-17141080 | Island | -0.11 | 0.000204332 |
| cg26588076 | chr17 | 17741631 | *SREBF1* | chr17:17743412-17743988 | N_Shore | -0.11 | 0.009410686 |
| cg14033514 | chr17 | 19483721 |  | chr17:19483305-19483687 | S_Shore | -0.11 | 0.004215452 |
| cg12065943 | chr17 | 19881925 | *AKAP10* | chr17:19883325-19883610 | N_Shore | -0.17 | 5.36E-08 |
| cg07435331 | chr17 | 21178476 |  | chr17:21178819-21179690 | N_Shore | -0.10 | 0.007527534 |
| cg13229782 | chr17 | 21179777 |  | chr17:21178819-21179690 | S_Shore | -0.11 | 0.001745201 |
| cg02620335 | chr17 | 25583370 |  |  | OpenSea | -0.15 | 0.00167278 |
| cg15704699 | chr17 | 26439377 | *NLK* |  | OpenSea | -0.10 | 0.005756942 |
| cg09479241 | chr17 | 27052676 | *TLCD1* | chr17:27052941-27054084 | N_Shore | -0.11 | 0.006443875 |
| cg08217285 | chr17 | 27917879 | *GIT1* | chr17:27918161-27918398 | N_Shore | -0.12 | 3.38E-06 |
| cg22584138 | chr17 | 28562220 | *SLC6A4* | chr17:28562387-28563186 | N_Shore | 0.14 | 9.84E-05 |
| cg19227924 | chr17 | 28565709 |  | chr17:28562387-28563186 | S_Shelf | 0.12 | 1.88E-06 |
| cg08896939 | chr17 | 29297380 | *RNF135* | chr17:29298046-29298606 | N_Shore | -0.13 | 0.002522937 |
| cg04193820 | chr17 | 29297414 | *RNF135* | chr17:29298046-29298606 | N_Shore | -0.12 | 0.006285923 |
| cg19250113 | chr17 | 29298832 | *RNF135* | chr17:29298046-29298606 | S_Shore | -0.19 | 2.42E-08 |
| cg19602315 | chr17 | 30669494 | *C17orf75* | chr17:30669000-30669287 | S_Shore | -0.14 | 0.002053769 |
| cg03237218 | chr17 | 33760527 | *SLFN12* |  | OpenSea | -0.11 | 0.0052223 |
| cg18470427 | chr17 | 33842301 | *SLFN12L* |  | OpenSea | 0.11 | 0.002544523 |
| cg04193015 | chr17 | 35596680 | *ACACA* |  | OpenSea | -0.14 | 0.002103494 |
| cg06549479 | chr17 | 36885900 | *CISD3* | chr17:36886108-36886525 | N_Shore | -0.16 | 2.69E-06 |
| cg27492942 | chr17 | 36885965 | *CISD3* | chr17:36886108-36886525 | N_Shore | -0.13 | 3.52E-06 |
| cg15445000 | chr17 | 37608096 | *MED1* | chr17:37607393-37607718 | S_Shore | -0.20 | 0.000167622 |
| cg04308185 | chr17 | 38084377 | *ORMDL3* | chr17:38083226-38084052 | S_Shore | -0.20 | 7.94E-05 |
| cg10444806 | chr17 | 38084428 | *ORMDL3* | chr17:38083226-38084052 | S_Shore | -0.14 | 0.008747524 |
| cg08932654 | chr17 | 38084459 | *ORMDL3* | chr17:38083226-38084052 | S_Shore | -0.14 | 0.000682233 |
| cg07438660 | chr17 | 38805042 | *SMARCE1* | chr17:38803944-38804703 | S_Shore | -0.11 | 0.000949235 |
| cg19443920 | chr17 | 40075879 | *ACLY* | chr17:40074291-40075380 | S_Shore | -0.11 | 0.001955854 |
| cg23797200 | chr17 | 40174494 | *NKIRAS2* | chr17:40171931-40172281 | S_Shelf | 0.13 | 0.000126495 |
| cg16824477 | chr17 | 40192449 |  | chr17:40191787-40192450 | Island | -0.10 | 0.003769246 |
| cg14787477 | chr17 | 40687596 | *NAGLU* | chr17:40688033-40688745 | N_Shore | -0.10 | 0.001408912 |
| cg18223939 | chr17 | 40687822 | *NAGLU* | chr17:40688033-40688745 | N_Shore | -0.16 | 1.64E-07 |
| cg16347155 | chr17 | 40687830 | *NAGLU* | chr17:40688033-40688745 | N_Shore | -0.12 | 9.43E-06 |
| cg15999590 | chr17 | 40718810 | *MLX* | chr17:40718686-40719633 | Island | -0.13 | 0.000103605 |
| cg20795913 | chr17 | 40718830 | *MLX* | chr17:40718686-40719633 | Island | -0.12 | 4.13E-05 |
| cg18647570 | chr17 | 41594616 | *DHX8* |  | OpenSea | -0.15 | 0.004817185 |
| cg15265102 | chr17 | 41797729 |  | chr17:41797861-41798381 | N_Shore | -0.13 | 1.46E-06 |
| cg20359349 | chr17 | 41994531 | *C17orf88* |  | OpenSea | -0.12 | 6.98E-05 |
| cg08685733 | chr17 | 42215609 |  | chr17:42219153-42219616 | N_Shelf | 0.11 | 0.005440451 |
| cg14686645 | chr17 | 42452426 | *ITGA2B* |  | OpenSea | 0.16 | 0.0026026 |
| cg22968622 | chr17 | 43663579 |  | chr17:43662141-43663807 | Island | 0.19 | 0.00619673 |
| cg05301556 | chr17 | 43971177 | *MAPT* | chr17:43971410-43975040 | N_Shore | -0.14 | 7.36E-05 |
| cg09764761 | chr17 | 44105544 | *MAPT* |  | OpenSea | -0.12 | 0.001326938 |
| cg08358943 | chr17 | 44889584 | *WNT3* |  | OpenSea | 0.13 | 1.81E-05 |
| cg17881353 | chr17 | 44928210 | *WNT9B* | chr17:44928287-44929690 | N_Shore | 0.13 | 0.002024987 |
| cg04650948 | chr17 | 46798298 |  | chr17:46796234-46797292 | S_Shore | -0.10 | 0.002282234 |
| cg07967210 | chr17 | 47022446 | *SNF8* | chr17:47022062-47022344 | S_Shore | -0.12 | 0.000236899 |
| cg10824354 | chr17 | 47049956 |  |  | OpenSea | 0.10 | 0.000418301 |
| cg18588024 | chr17 | 47645609 |  | chr17:47645365-47645833 | Island | 0.10 | 0.000295964 |
| cg27490916 | chr17 | 47645754 |  | chr17:47645365-47645833 | Island | 0.10 | 0.001429919 |
| cg07871456 | chr17 | 48796501 | *LUC7L3* | chr17:48796750-48797317 | N_Shore | -0.14 | 2.31E-06 |
| cg04604142 | chr17 | 48796515 | *LUC7L3* | chr17:48796750-48797317 | N_Shore | -0.13 | 7.22E-05 |
| cg20640281 | chr17 | 53341592 | *HLF* | chr17:53342198-53343061 | N_Shore | -0.12 | 2.85E-07 |
| cg07477034 | chr17 | 53341969 | *HLF* | chr17:53342198-53343061 | N_Shore | -0.12 | 1.19E-06 |
| cg18040354 | chr17 | 53800484 | *TMEM100* |  | OpenSea | 0.19 | 9.34E-05 |
| cg20047349 | chr17 | 54305680 | *ANKFN1* |  | OpenSea | -0.13 | 0.007224433 |
| cg14593033 | chr17 | 56296980 | *MKS1* | chr17:56296488-56296842 | S_Shore | -0.15 | 2.69E-10 |
| cg07908160 | chr17 | 56296982 | *MKS1* | chr17:56296488-56296842 | S_Shore | -0.16 | 3.17E-11 |
| cg20464360 | chr17 | 56564855 | *HSF5* | chr17:56564854-56565891 | Island | -0.10 | 0.002221112 |
| cg00443981 | chr17 | 58499679 | *C17orf64* | chr17:58498711-58499332 | S_Shore | -0.21 | 2.81E-05 |
| cg12131208 | chr17 | 58499700 | *C17orf64* | chr17:58498711-58499332 | S_Shore | -0.26 | 1.07E-05 |
| cg04413853 | chr17 | 58499706 | *C17orf64* | chr17:58498711-58499332 | S_Shore | -0.19 | 7.55E-05 |
| cg21122199 | chr17 | 58499720 | *C17orf64* | chr17:58498711-58499332 | S_Shore | -0.16 | 0.00167278 |
| cg06752482 | chr17 | 58499816 | *C17orf64* | chr17:58498711-58499332 | S_Shore | -0.12 | 0.000116097 |
| cg17628249 | chr17 | 58499854 | *C17orf64* | chr17:58498711-58499332 | S_Shore | -0.17 | 3.35E-05 |
| cg02172058 | chr17 | 58499911 | *C17orf64* | chr17:58498711-58499332 | S_Shore | -0.17 | 0.000236899 |
| cg18223453 | chr17 | 59554746 | *TBX4* |  | OpenSea | 0.11 | 0.004949124 |
| cg02988947 | chr17 | 61778813 | *LIMD2* | chr17:61776890-61778733 | S_Shore | -0.13 | 1.16E-08 |
| cg06873352 | chr17 | 61820015 | *STRADA* | chr17:61818684-61819397 | S_Shore | -0.13 | 0.000662215 |
| cg11122009 | chr17 | 63554536 | *AXIN2* | chr17:63555902-63557362 | N_Shore | 0.12 | 0.000162284 |
| cg21809624 | chr17 | 63569403 |  |  | OpenSea | 0.11 | 0.00368334 |
| cg00874073 | chr17 | 73073329 |  | chr17:73073313-73073941 | Island | -0.11 | 0.000897697 |
| cg19804488 | chr17 | 73760363 | *GALK1* | chr17:73760694-73761269 | N_Shore | -0.12 | 1.40E-05 |
| cg25814096 | chr17 | 73782472 | *UNK* | chr17:73780846-73781308 | S_Shore | -0.10 | 0.000649603 |
| cg01453052 | chr17 | 73892097 | *TRIM65* | chr17:73892496-73893448 | N_Shore | -0.14 | 4.99E-05 |
| cg08122232 | chr17 | 74100132 | *EXOC7* | chr17:74099738-74100055 | S_Shore | -0.40 | 1.55E-20 |
| cg25699034 | chr17 | 74721824 | *C17orf95* | chr17:74722031-74723420 | N_Shore | -0.11 | 0.001940304 |
| cg14625581 | chr17 | 75692478 |  |  | OpenSea | 0.12 | 0.006909707 |
| cg14652587 | chr17 | 75692571 |  |  | OpenSea | 0.17 | 0.006120504 |
| cg22061523 | chr17 | 76183551 | *AFMID* | chr17:76183077-76183449 | S_Shore | -0.10 | 3.82E-05 |
| cg22892904 | chr17 | 77751089 | *CBX2* | chr17:77751378-77751673 | N_Shore | -0.18 | 4.25E-11 |
| cg03000846 | chr17 | 79990263 | *RAC3* | chr17:79987983-79990315 | Island | -0.11 | 0.000137592 |
| cg07343027 | chr17 | 80024200 | *DUS1L* | chr17:80022655-80024178 | S_Shore | -0.11 | 0.000110691 |
| cg07529654 | chr18 | 3447016 | *TGIF1* | chr18:3448006-3452360 | N_Shore | -0.15 | 0.008922497 |
| cg25279586 | chr18 | 7566258 | *PTPRM* | chr18:7566557-7568830 | N_Shore | -0.11 | 0.001522453 |
| cg14910288 | chr18 | 10033220 |  | chr18:10032458-10032907 | S_Shore | -0.35 | 2.22E-09 |
| cg05331731 | chr18 | 11147146 | *FAM38B* | chr18:11148307-11149936 | N_Shore | 0.14 | 9.44E-05 |
| cg06268875 | chr18 | 11147385 | *FAM38B* | chr18:11148307-11149936 | N_Shore | 0.29 | 1.16E-05 |
| cg12049992 | chr18 | 11147785 | *FAM38B* | chr18:11148307-11149936 | N_Shore | 0.19 | 1.65E-05 |
| cg11926473 | chr18 | 11980297 | *IMPA2* | chr18:11980483-11982142 | N_Shore | -0.16 | 6.42E-11 |
| cg25836232 | chr18 | 12306837 | *TUBB6* | chr18:12307554-12309043 | N_Shore | 0.14 | 0.001261497 |
| cg13924974 | chr18 | 12656824 | *SPIRE1* | chr18:12657044-12658727 | N_Shore | -0.14 | 0.004319543 |
| cg25043559 | chr18 | 12948780 | *SEH1L* | chr18:12947924-12948654 | S_Shore | -0.26 | 6.70E-24 |
| cg21235532 | chr18 | 19476870 |  | chr18:19476852-19477103 | Island | 0.13 | 0.000778906 |
| cg10092257 | chr18 | 19476959 |  | chr18:19476852-19477103 | Island | 0.12 | 0.000856494 |
| cg07669403 | chr18 | 19477059 |  | chr18:19476852-19477103 | Island | 0.15 | 0.007977873 |
| cg02962521 | chr18 | 19744694 |  | chr18:19744936-19752363 | N_Shore | 0.13 | 0.000152354 |
| cg20528338 | chr18 | 21976748 | *OSBPL1A* | chr18:21977275-21978110 | N_Shore | -0.12 | 0.004729549 |
| cg03614916 | chr18 | 22007271 | *IMPACT* | chr18:22006310-22007007 | S_Shore | -0.20 | 3.42E-12 |
| cg06033764 | chr18 | 29671448 | *RNF138* | chr18:29671757-29673314 | N_Shore | -0.11 | 0.000189428 |
| cg16245698 | chr18 | 43355989 |  | chr18:43355318-43355698 | S_Shore | -0.21 | 2.26E-05 |
| cg23376861 | chr18 | 43678713 | *ATP5A1* | chr18:43677956-43678352 | S_Shore | -0.11 | 2.72E-07 |
| cg07912766 | chr18 | 45458698 | *SMAD2* | chr18:45456484-45458024 | S_Shore | -0.13 | 0.000841007 |
| cg21616935 | chr18 | 47794231 | *CCDC11* | chr18:47794450-47794996 | N_Shore | -0.13 | 0.001421495 |
| cg00152799 | chr18 | 54306261 | *TXNL1* | chr18:54305419-54306087 | S_Shore | -0.12 | 0.007590942 |
| cg24431033 | chr18 | 54306437 | *TXNL1* | chr18:54305419-54306087 | S_Shore | -0.16 | 0.000502071 |
| cg18687085 | chr18 | 56067462 | *NEDD4L* |  | OpenSea | -0.10 | 0.000909147 |
| cg06487082 | chr18 | 56530106 | *ZNF532* | chr18:56530395-56531288 | N_Shore | -0.14 | 7.09E-08 |
| cg12406559 | chr18 | 56530302 | *ZNF532* | chr18:56530395-56531288 | N_Shore | -0.16 | 1.27E-08 |
| cg14613540 | chr18 | 61616369 | *HMSD* |  | OpenSea | 0.11 | 0.004049396 |
| cg19283806 | chr18 | 66389420 | *CCDC102B* |  | OpenSea | -0.13 | 0.008661772 |
| cg25993608 | chr18 | 76621396 |  | chr18:76616616-76617519 | S_Shelf | -0.13 | 0.003132457 |
| cg06556497 | chr18 | 76828521 | *ATP9B* | chr18:76828638-76829796 | N_Shore | -0.28 | 2.59E-12 |
| cg08617160 | chr19 | 345312 | *MIER2* | chr19:345260-345590 | Island | -0.13 | 0.006653941 |
| cg09759458 | chr19 | 480216 |  |  | OpenSea | 0.18 | 0.004808704 |
| cg01613691 | chr19 | 1105380 | *GPX4* | chr19:1103722-1105758 | Island | -0.14 | 0.000230521 |
| cg01924434 | chr19 | 1275121 | *C19orf24* | chr19:1274469-1276358 | Island | -0.12 | 2.60E-07 |
| cg06653052 | chr19 | 1275167 | *C19orf24* | chr19:1274469-1276358 | Island | -0.15 | 5.24E-07 |
| cg24229188 | chr19 | 3576629 | *HMG20B* | chr19:3576628-3578314 | Island | -0.17 | 9.84E-05 |
| cg04770751 | chr19 | 7701662 | *STXBP2* | chr19:7701661-7702336 | Island | -0.10 | 3.35E-05 |
| cg23850205 | chr19 | 7967913 | *MAP2K7* | chr19:7968325-7969421 | N_Shore | -0.13 | 0.000319538 |
| cg27497751 | chr19 | 7967946 | *MAP2K7* | chr19:7968325-7969421 | N_Shore | -0.14 | 0.000343986 |
| cg06686742 | chr19 | 8273505 | *LASS4* | chr19:8273530-8273920 | N_Shore | 0.19 | 0.005107554 |
| cg08283130 | chr19 | 8591776 | *MYO1F* | chr19:8591294-8591842 | Island | -0.12 | 0.000843846 |
| cg19474047 | chr19 | 10207448 | *ANGPTL6* | chr19:10206624-10207351 | S_Shore | -0.25 | 1.46E-05 |
| cg01875838 | chr19 | 10947446 | *TMED1* | chr19:10946516-10947416 | S_Shore | -0.17 | 0.002225046 |
| cg13520520 | chr19 | 11616957 | *ECSIT* | chr19:11616031-11616847 | S_Shore | -0.16 | 4.67E-10 |
| cg21517055 | chr19 | 14016717 | *C19orf57* | chr19:14016665-14017435 | Island | -0.12 | 3.92E-08 |
| cg03621001 | chr19 | 16221719 | *RAB8A* | chr19:16222112-16223152 | N_Shore | -0.16 | 8.81E-07 |
| cg24878115 | chr19 | 18545062 | *SSBP4* | chr19:18543828-18549161 | Island | -0.17 | 0.000218446 |
| cg01065977 | chr19 | 18549689 | *ISYNA1* | chr19:18543828-18549161 | S_Shore | -0.12 | 0.003576507 |
| cg19492423 | chr19 | 18700933 | *C19orf60* | chr19:18699422-18700554 | S_Shore | -0.25 | 7.39E-07 |
| cg21977234 | chr19 | 18701320 | *C19orf60* | chr19:18699422-18700554 | S_Shore | -0.24 | 5.30E-05 |
| cg04661001 | chr19 | 19217217 | *SLC25A42* | chr19:19221196-19221575 | N_Shelf | -0.14 | 0.003893521 |
| cg02887458 | chr19 | 19495540 | *GATAD2A* | chr19:19495952-19497038 | N_Shore | -0.14 | 0.007234475 |
| cg25274157 | chr19 | 19626576 | *NDUFA13* | chr19:19624954-19627258 | Island | -0.12 | 0.000439622 |
| cg08331981 | chr19 | 19626599 | *NDUFA13* | chr19:19624954-19627258 | Island | -0.12 | 0.000610292 |
| cg03233793 | chr19 | 19626605 | *NDUFA13* | chr19:19624954-19627258 | Island | -0.13 | 0.000197768 |
| cg03657045 | chr19 | 29704422 | *UQCRFS1* | chr19:29703570-29704331 | S_Shore | -0.13 | 3.66E-05 |
| cg05516285 | chr19 | 29704446 | *UQCRFS1* | chr19:29703570-29704331 | S_Shore | -0.11 | 0.001922325 |
| cg23458168 | chr19 | 30864867 | *ZNF536* | chr19:30865683-30866490 | N_Shore | 0.13 | 0.000468969 |
| cg16043651 | chr19 | 30865486 | *ZNF536* | chr19:30865683-30866490 | N_Shore | 0.11 | 0.000170077 |
| cg21498471 | chr19 | 33183713 | *NUDT19* | chr19:33182609-33183562 | S_Shore | -0.16 | 1.40E-07 |
| cg21715751 | chr19 | 33795390 | *LOC80054* | chr19:33792061-33794944 | S_Shore | -0.10 | 0.006262771 |
| cg03339609 | chr19 | 34662636 | *LSM14A* | chr19:34662931-34664060 | N_Shore | -0.12 | 9.67E-11 |
| cg04731926 | chr19 | 35758185 | *LSR* | chr19:35758201-35759670 | N_Shore | -0.13 | 2.51E-06 |
| cg19556473 | chr19 | 37894856 |  | chr19:37894343-37894865 | Island | -0.19 | 0.000947455 |
| cg20700792 | chr19 | 38042009 | *ZNF540* | chr19:38039561-38040395 | S_Shore | -0.10 | 0.002753137 |
| cg15135657 | chr19 | 38346511 |  | chr19:38345808-38346160 | S_Shore | 0.18 | 3.14E-05 |
| cg19261810 | chr19 | 38754529 | *SPINT2* | chr19:38754846-38755699 | N_Shore | -0.12 | 5.90E-05 |
| cg07859880 | chr19 | 39323060 | *ECH1* | chr19:39322264-39322574 | S_Shore | -0.11 | 0.001452993 |
| cg05609536 | chr19 | 41019285 | *SPTBN4* | chr19:41018525-41019486 | Island | 0.12 | 0.004199337 |
| cg02059080 | chr19 | 44124666 | *ZNF428* | chr19:44123485-44124244 | S_Shore | -0.10 | 0.000259247 |
| cg25786696 | chr19 | 44406241 |  | chr19:44405817-44406053 | S_Shore | -0.10 | 0.003488309 |
| cg01057656 | chr19 | 45281140 | *CBLC* | chr19:45281133-45281355 | Island | 0.14 | 0.00088654 |
| cg16529268 | chr19 | 45281284 | *CBLC* | chr19:45281133-45281355 | Island | 0.16 | 0.000114934 |
| cg04683509 | chr19 | 45885800 | *PPP1R13L* | chr19:45885786-45885999 | Island | 0.11 | 0.002643334 |
| cg06484000 | chr19 | 46998383 | *PNMAL2* | chr19:46996327-46998437 | Island | 0.12 | 0.005763646 |
| cg02026498 | chr19 | 47139338 | *GNG8* | chr19:47139337-47139547 | Island | 0.13 | 1.27E-05 |
| cg27550511 | chr19 | 47139496 |  | chr19:47139337-47139547 | Island | 0.20 | 0.000542421 |
| cg27333886 | chr19 | 47713807 | *SAE1* |  | OpenSea | -0.11 | 0.005599037 |
| cg03950599 | chr19 | 48698419 |  | chr19:48697900-48698669 | Island | 0.21 | 1.62E-06 |
| cg05017628 | chr19 | 48698632 |  | chr19:48697900-48698669 | Island | 0.23 | 0.000589869 |
| cg19477346 | chr19 | 48699872 |  | chr19:48697900-48698669 | S_Shore | 0.15 | 0.005225206 |
| cg21519787 | chr19 | 49133421 | *SPHK2* | chr19:49132603-49134291 | Island | -0.10 | 0.004383805 |
| cg21486694 | chr19 | 49957559 | *ALDH16A1* | chr19:49956279-49956837 | S_Shore | -0.12 | 0.004199337 |
| cg19309676 | chr19 | 50191439 | *C19orf76* | chr19:50191330-50191559 | Island | -0.10 | 0.006315866 |
| cg22525294 | chr19 | 50249464 | *TSKS* | chr19:50249492-50249894 | N_Shore | 0.14 | 0.001917534 |
| cg08194323 | chr19 | 50862004 | *NAPSA* | chr19:50861773-50862083 | Island | -0.14 | 8.60E-06 |
| cg26456183 | chr19 | 50862121 | *NAPSA* | chr19:50860846-50861263 | S_Shore | -0.12 | 0.00114525 |
| cg26812169 | chr19 | 51601823 | *CTU1* | chr19:51601822-51602260 | Island | -0.12 | 0.000715057 |
| cg26703182 | chr19 | 51601884 | *CTU1* | chr19:51601822-51602260 | Island | -0.22 | 4.97E-06 |
| cg27500647 | chr19 | 51602230 | *CTU1* | chr19:51601822-51602260 | Island | -0.15 | 0.000398674 |
| cg01413268 | chr19 | 51602462 | *CTU1* | chr19:51601822-51602260 | S_Shore | -0.18 | 0.000889076 |
| cg19806182 | chr19 | 52511900 | *ZNF615* | chr19:52511228-52511582 | S_Shore | -0.13 | 7.41E-05 |
| cg05308495 | chr19 | 54515169 | *CACNG6* | chr19:54515094-54515342 | Island | 0.23 | 3.98E-05 |
| cg10648815 | chr19 | 55013549 | *LAIR2* |  | OpenSea | 0.21 | 0.006845752 |
| cg19504245 | chr19 | 55660620 | *TNNT1* |  | OpenSea | 0.11 | 0.008758776 |
| cg03015952 | chr19 | 55987366 | *ZNF628* | chr19:55987544-55988171 | N_Shore | -0.24 | 2.15E-13 |
| cg12989534 | chr19 | 55987403 | *ZNF628* | chr19:55987544-55988171 | N_Shore | -0.13 | 1.22E-10 |
| cg01290153 | chr19 | 55987415 | *ZNF628* | chr19:55987544-55988171 | N_Shore | -0.19 | 5.70E-10 |
| cg24170248 | chr19 | 56015067 | *SSC5D* | chr19:56014811-56015333 | Island | 0.11 | 0.000159889 |
| cg08324152 | chr19 | 57172404 |  | chr19:57175061-57176407 | N_Shelf | 0.13 | 0.00958213 |
| cg26644049 | chr19 | 57276917 |  | chr19:57276614-57276942 | Island | -0.13 | 0.000955748 |
| cg16519742 | chr19 | 57306847 | *ZIM2* | chr19:57306682-57307032 | Island | 0.10 | 0.001990636 |
| cg22951411 | chr19 | 57375307 |  | chr19:57375842-57376422 | N_Shore | -0.12 | 0.000263331 |
| cg06627087 | chr19 | 57376177 |  | chr19:57375842-57376422 | Island | -0.17 | 0.001184494 |
| cg19568003 | chr19 | 57742217 | *AURKC* | chr19:57741955-57742457 | Island | -0.13 | 0.004873685 |
| cg18644286 | chr19 | 57742255 | *AURKC* | chr19:57741955-57742457 | Island | -0.16 | 0.006067593 |
| cg26332114 | chr19 | 57742260 | *AURKC* | chr19:57741955-57742457 | Island | -0.15 | 0.001236725 |
| cg19603903 | chr19 | 57742345 | *AURKC* | chr19:57741955-57742457 | Island | -0.17 | 0.001441544 |
| cg23371413 | chr19 | 57742394 | *AURKC* | chr19:57741955-57742457 | Island | -0.13 | 0.003971891 |
| cg25432232 | chr19 | 57742423 | *AURKC* | chr19:57741955-57742457 | Island | -0.16 | 0.004285129 |
| cg22711741 | chr19 | 57742444 | *AURKC* | chr19:57741955-57742457 | Island | -0.17 | 0.004266676 |
| cg26282792 | chr19 | 58554479 | *ZSCAN1* | chr19:58554354-58554587 | Island | 0.14 | 0.000347659 |
| cg25648436 | chr19 | 58554527 | *ZSCAN1* | chr19:58554354-58554587 | Island | 0.14 | 2.79E-05 |
| cg18075691 | chr19 | 58566643 |  | chr19:58570393-58571779 | N_Shelf | 0.18 | 0.004555574 |
| cg08701621 | chr19 | 58570419 | *ZNF135* | chr19:58570393-58571779 | Island | 0.14 | 1.32E-06 |
| cg06454760 | chr19 | 58570427 | *ZNF135* | chr19:58570393-58571779 | Island | 0.13 | 3.29E-05 |
| cg02473540 | chr19 | 58570454 | *ZNF135* | chr19:58570393-58571779 | Island | 0.15 | 0.001784593 |
| cg09907936 | chr19 | 58570466 | *ZNF135* | chr19:58570393-58571779 | Island | 0.14 | 3.35E-06 |
| cg18430128 | chr19 | 58570491 | *ZNF135* | chr19:58570393-58571779 | Island | 0.11 | 4.95E-06 |
| cg07502936 | chr19 | 58571638 | *ZNF135* | chr19:58570393-58571779 | Island | -0.16 | 0.004665981 |
| cg11701312 | chr19 | 58897497 | *RPS5* | chr19:58897721-58898878 | N_Shore | -0.20 | 8.84E-13 |
| cg06638023 | chr19 | 59030102 | *ZBTB45* | chr19:59030657-59031426 | N_Shore | -0.13 | 5.36E-07 |
| cg27379715 | chr2 | 875990 |  |  | OpenSea | 0.10 | 0.001323042 |
| cg22745354 | chr2 | 928720 |  |  | OpenSea | 0.11 | 0.000109771 |
| cg23953176 | chr2 | 947515 | *SNTG2* | chr2:945686-946774 | S_Shore | 0.15 | 0.000185147 |
| cg20193363 | chr2 | 947589 | *SNTG2* | chr2:945686-946774 | S_Shore | 0.19 | 0.000123312 |
| cg11691429 | chr2 | 947634 | *SNTG2* | chr2:945686-946774 | S_Shore | 0.15 | 0.0005239 |
| cg05422049 | chr2 | 1595734 |  | chr2:1595402-1596020 | Island | 0.13 | 0.007486158 |
| cg05854217 | chr2 | 1595819 |  | chr2:1595402-1596020 | Island | 0.10 | 0.001708825 |
| cg03550129 | chr2 | 1596054 |  | chr2:1595402-1596020 | S_Shore | 0.12 | 0.000259949 |
| cg01328473 | chr2 | 1711966 | *PXDN* |  | OpenSea | -0.12 | 0.009331624 |
| cg17801352 | chr2 | 1749076 | *PXDN* | chr2:1746833-1748971 | S_Shore | -0.19 | 0.000491732 |
| cg24201793 | chr2 | 9144764 | *MBOAT2* | chr2:9143127-9144630 | S_Shore | -0.31 | 2.28E-09 |
| cg14853772 | chr2 | 9613781 | *IAH1* | chr2:9614194-9615235 | N_Shore | -0.11 | 0.007933857 |
| cg07712663 | chr2 | 16789846 | *FAM49A* |  | OpenSea | -0.11 | 0.000269968 |
| cg01227744 | chr2 | 20644371 |  | chr2:20646428-20647988 | N_Shelf | 0.15 | 0.002735753 |
| cg14273083 | chr2 | 24306495 | *TP53I3* | chr2:24307026-24307678 | N_Shore | -0.13 | 0.000277842 |
| cg08965143 | chr2 | 24308246 | *TP53I3* | chr2:24307026-24307678 | S_Shore | -0.12 | 0.000204934 |
| cg03879180 | chr2 | 24713283 |  | chr2:24713710-24715168 | N_Shore | -0.12 | 0.000660732 |
| cg23809645 | chr2 | 25383851 | *POMC* | chr2:25383939-25384763 | N_Shore | -0.40 | 2.29E-10 |
| cg10045137 | chr2 | 25383940 | *POMC* | chr2:25383939-25384763 | Island | -0.33 | 1.00E-08 |
| cg02716646 | chr2 | 25384293 | *POMC* | chr2:25383939-25384763 | Island | -0.28 | 4.42E-12 |
| cg06846259 | chr2 | 25384654 | *POMC* | chr2:25383939-25384763 | Island | -0.33 | 3.53E-08 |
| cg20807790 | chr2 | 25384762 | *POMC* | chr2:25383939-25384763 | Island | -0.17 | 1.61E-06 |
| cg02757179 | chr2 | 25384809 | *POMC* | chr2:25383939-25384763 | S_Shore | -0.21 | 4.09E-05 |
| cg20422417 | chr2 | 25427108 |  | chr2:25427101-25427577 | Island | -0.36 | 4.61E-09 |
| cg02757970 | chr2 | 25427350 |  | chr2:25427101-25427577 | Island | -0.19 | 0.00210458 |
| cg13349472 | chr2 | 25427451 |  | chr2:25427101-25427577 | Island | -0.18 | 0.000206798 |
| cg13430552 | chr2 | 25427652 |  | chr2:25427101-25427577 | S_Shore | -0.16 | 7.23E-05 |
| cg01738095 | chr2 | 26101831 | *ASXL2* | chr2:26100513-26101653 | S_Shore | -0.12 | 0.001810591 |
| cg22284448 | chr2 | 27008234 | *CENPA* | chr2:27008760-27009465 | N_Shore | -0.15 | 0.003670635 |
| cg06293611 | chr2 | 27308434 | *EMILIN1* | chr2:27308546-27309770 | N_Shore | -0.12 | 0.00013099 |
| cg00719165 | chr2 | 27603886 | *ZNF513* | chr2:27603350-27604064 | Island | -0.10 | 2.19E-05 |
| cg11177693 | chr2 | 27604063 | *ZNF513* | chr2:27603350-27604064 | Island | -0.14 | 4.70E-05 |
| cg21709322 | chr2 | 27632821 | *PPM1G* | chr2:27631884-27632822 | Island | -0.15 | 6.42E-11 |
| cg15661865 | chr2 | 27632926 | *PPM1G* | chr2:27631884-27632822 | S_Shore | -0.17 | 4.00E-07 |
| cg25309888 | chr2 | 27988724 |  |  | OpenSea | -0.13 | 0.000836453 |
| cg06142740 | chr2 | 28973577 | *PPP1CB* | chr2:28974212-28975388 | N_Shore | -0.12 | 1.98E-11 |
| cg14126601 | chr2 | 37384708 | *EIF2AK2* | chr2:37383901-37384402 | S_Shore | -0.13 | 0.001638234 |
| cg14036868 | chr2 | 38604442 | *ATL2* | chr2:38603569-38604510 | Island | 0.12 | 0.005680907 |
| cg20043649 | chr2 | 47537239 |  |  | OpenSea | -0.14 | 0.005754058 |
| cg03936870 | chr2 | 47561578 |  |  | OpenSea | -0.18 | 1.61E-06 |
| cg03664994 | chr2 | 55246602 | *RTN4* |  | OpenSea | -0.13 | 0.004715174 |
| cg03092551 | chr2 | 58273420 | *VRK2* | chr2:58273642-58274422 | N_Shore | -0.18 | 5.11E-08 |
| cg20604286 | chr2 | 58273438 | *VRK2* | chr2:58273642-58274422 | N_Shore | -0.18 | 2.50E-08 |
| cg03754165 | chr2 | 60780427 | *BCL11A* | chr2:60781019-60782284 | N_Shore | -0.11 | 0.009080138 |
| cg10580144 | chr2 | 61372316 | *C2orf74* |  | OpenSea | -0.12 | 0.000129462 |
| cg18206867 | chr2 | 62422351 | *B3GNT2* | chr2:62422191-62424458 | Island | -0.18 | 0.000234556 |
| cg08241318 | chr2 | 64885581 |  | chr2:64880319-64881868 | S_Shelf | -0.13 | 0.008440678 |
| cg09550083 | chr2 | 66672337 | *MEIS1* | chr2:66672431-66673636 | N_Shore | -0.10 | 0.008904155 |
| cg25195673 | chr2 | 69614945 | *GFPT1* | chr2:69614119-69614616 | S_Shore | -0.14 | 0.000320842 |
| cg00752969 | chr2 | 70311984 |  | chr2:70313200-70315490 | N_Shore | -0.30 | 1.52E-10 |
| cg16450677 | chr2 | 70312492 |  | chr2:70313200-70315490 | N_Shore | -0.40 | 2.11E-18 |
| cg19638749 | chr2 | 70312615 |  | chr2:70313200-70315490 | N_Shore | -0.10 | 0.000463825 |
| cg08479476 | chr2 | 70313163 | *PCBP1* | chr2:70313200-70315490 | N_Shore | -0.23 | 1.29E-20 |
| cg26474124 | chr2 | 70368457 |  | chr2:70369139-70370130 | N_Shore | -0.10 | 0.000297468 |
| cg20097407 | chr2 | 71112079 |  | chr2:71114733-71115474 | N_Shelf | -0.16 | 0.000743639 |
| cg19015611 | chr2 | 71126446 | *VAX2* | chr2:71126720-71129209 | N_Shore | -0.20 | 0.002859525 |
| cg21918313 | chr2 | 73496203 | *FBXO41* | chr2:73495920-73496910 | Island | 0.14 | 0.003444677 |
| cg00610692 | chr2 | 74668072 | *RTKN* | chr2:74667593-74669403 | Island | -0.12 | 0.001236827 |
| cg09692695 | chr2 | 74668286 | *RTKN* | chr2:74667593-74669403 | Island | -0.15 | 0.001442484 |
| cg00689340 | chr2 | 74668672 | *RTKN* | chr2:74667593-74669403 | Island | -0.12 | 0.00214906 |
| cg20415811 | chr2 | 74700246 | *CCDC142* | chr2:74699240-74699909 | S_Shore | -0.11 | 3.92E-08 |
| cg20520115 | chr2 | 75063838 | *HK2* | chr2:75060879-75062664 | S_Shore | -0.10 | 0.000185726 |
| cg16229671 | chr2 | 75937132 | *C2orf3* | chr2:75937657-75938139 | N_Shore | -0.14 | 3.79E-05 |
| cg11051158 | chr2 | 75938223 | *C2orf3* | chr2:75937657-75938139 | S_Shore | -0.12 | 3.27E-07 |
| cg20704342 | chr2 | 75938273 | *C2orf3* | chr2:75937657-75938139 | S_Shore | -0.10 | 1.64E-07 |
| cg23051299 | chr2 | 75938289 | *C2orf3* | chr2:75937657-75938139 | S_Shore | -0.13 | 1.38E-05 |
| cg01044662 | chr2 | 75938360 | *C2orf3* | chr2:75937657-75938139 | S_Shore | -0.20 | 3.41E-07 |
| cg23725986 | chr2 | 75938438 | *C2orf3* | chr2:75937657-75938139 | S_Shore | -0.20 | 8.28E-08 |
| cg22975318 | chr2 | 84524975 |  |  | OpenSea | -0.15 | 0.000255122 |
| cg06804210 | chr2 | 87036948 | *CD8A* | chr2:87036600-87037001 | Island | 0.14 | 0.002056221 |
| cg27481559 | chr2 | 87037038 |  | chr2:87036600-87037001 | S_Shore | 0.14 | 0.003268307 |
| cg14410476 | chr2 | 95693023 | *MAL* | chr2:95690857-95692431 | S_Shore | 0.13 | 0.003402527 |
| cg21475150 | chr2 | 101618248 | *RPL31* | chr2:101618247-101618658 | Island | -0.25 | 7.55E-05 |
| cg22809047 | chr2 | 101618261 | *RPL31* | chr2:101618247-101618658 | Island | -0.13 | 0.000662215 |
| cg25428553 | chr2 | 105275706 |  | chr2:105275588-105275940 | Island | -0.10 | 0.008837073 |
| cg12236088 | chr2 | 105275812 |  | chr2:105275588-105275940 | Island | -0.13 | 0.005819298 |
| cg08571918 | chr2 | 105275920 |  | chr2:105275588-105275940 | Island | -0.11 | 0.006686739 |
| cg22360016 | chr2 | 105276153 |  | chr2:105275588-105275940 | S_Shore | -0.13 | 0.007870073 |
| cg24213669 | chr2 | 118616576 |  | chr2:118616575-118618163 | Island | 0.18 | 0.001657563 |
| cg00046336 | chr2 | 118617230 |  | chr2:118616575-118618163 | Island | 0.11 | 0.001451868 |
| cg22545206 | chr2 | 118617499 |  | chr2:118616575-118618163 | Island | 0.22 | 0.000448671 |
| cg03217115 | chr2 | 118942841 |  | chr2:118943823-118944199 | N_Shore | -0.11 | 0.001005263 |
| cg27637303 | chr2 | 118942893 |  | chr2:118943823-118944199 | N_Shore | -0.15 | 0.000730302 |
| cg20234855 | chr2 | 120980555 | *TMEM185B* | chr2:120980332-120981451 | Island | -0.10 | 4.23E-05 |
| cg25334934 | chr2 | 121269348 |  |  | OpenSea | 0.20 | 3.99E-05 |
| cg07218647 | chr2 | 121338498 |  | chr2:121334308-121334801 | S_Shelf | 0.13 | 5.63E-06 |
| cg09408902 | chr2 | 121338544 |  | chr2:121334308-121334801 | S_Shelf | 0.12 | 0.000541192 |
| cg15344192 | chr2 | 121347210 |  | chr2:121344550-121345341 | S_Shore | -0.15 | 0.000603763 |
| cg13872898 | chr2 | 121498194 |  | chr2:121499228-121499578 | N_Shore | 0.11 | 0.007675066 |
| cg17870997 | chr2 | 121498521 |  | chr2:121499228-121499578 | N_Shore | 0.15 | 0.002712047 |
| cg03566527 | chr2 | 121749280 | *GLI2* | chr2:121745840-121746851 | S_Shelf | -0.15 | 0.001133568 |
| cg19153828 | chr2 | 127782651 |  | chr2:127782613-127782829 | Island | 0.12 | 0.00038524 |
| cg00255882 | chr2 | 128569347 | *WDR33* | chr2:128568321-128569064 | S_Shore | -0.11 | 0.00233405 |
| cg26509250 | chr2 | 134884342 |  |  | OpenSea | -0.12 | 0.00675161 |
| cg24608381 | chr2 | 139538746 | *NXPH2* | chr2:139537692-139538650 | S_Shore | 0.16 | 0.006297916 |
| cg00782200 | chr2 | 145280523 |  | chr2:145281736-145282269 | N_Shore | 0.19 | 0.001007634 |
| cg14420245 | chr2 | 148601095 | *ACVR2A* | chr2:148601280-148602859 | N_Shore | -0.12 | 8.99E-07 |
| cg14379965 | chr2 | 150177134 |  | chr2:150176813-150177219 | Island | 0.13 | 0.003528985 |
| cg08719380 | chr2 | 152830505 | *CACNB4* |  | OpenSea | 0.10 | 4.00E-05 |
| cg11025974 | chr2 | 152830521 | *CACNB4* |  | OpenSea | 0.13 | 7.23E-07 |
| cg21376136 | chr2 | 152830572 | *CACNB4* |  | OpenSea | 0.15 | 2.40E-05 |
| cg27664689 | chr2 | 152830665 | *CACNB4* |  | OpenSea | 0.14 | 7.42E-06 |
| cg02276944 | chr2 | 157291826 | *GPD2* | chr2:157292102-157292646 | N_Shore | -0.11 | 0.003254966 |
| cg16682903 | chr2 | 158694670 | *ACVR1* |  | OpenSea | -0.19 | 8.49E-05 |
| cg07151747 | chr2 | 158977473 | *UPP2* |  | OpenSea | -0.15 | 0.001763992 |
| cg09357934 | chr2 | 159651918 | *DAPL1* |  | OpenSea | 0.11 | 0.00586569 |
| cg17319011 | chr2 | 161137002 | *RBMS1* |  | OpenSea | 0.17 | 0.002083477 |
| cg01620164 | chr2 | 164590272 | *FIGN* | chr2:164592917-164593511 | N_Shelf | 0.13 | 0.003623537 |
| cg14771658 | chr2 | 167350883 |  |  | OpenSea | 0.11 | 0.004671282 |
| cg05961700 | chr2 | 169937750 | *DHRS9* |  | OpenSea | 0.12 | 0.00091516 |
| cg09281979 | chr2 | 172289850 | *DCAF17* | chr2:172290364-172291201 | N_Shore | -0.18 | 1.83E-06 |
| cg26817877 | chr2 | 173539621 |  |  | OpenSea | 0.11 | 0.000211175 |
| cg14510926 | chr2 | 174877357 |  | chr2:174877565-174877778 | N_Shore | -0.11 | 0.000300237 |
| cg03478739 | chr2 | 175712381 | *CHN1* |  | OpenSea | 0.11 | 0.008454196 |
| cg16874669 | chr2 | 189237094 | *GULP1* |  | OpenSea | -0.19 | 0.003710902 |
| cg19094243 | chr2 | 193058628 | *TMEFF2* | chr2:193058995-193060812 | N_Shore | 0.14 | 0.000155575 |
| cg03774803 | chr2 | 198650880 | *BOLL* | chr2:198649751-198651599 | Island | 0.12 | 0.003528985 |
| cg00926400 | chr2 | 198651498 | *BOLL* | chr2:198649751-198651599 | Island | 0.14 | 0.000254594 |
| cg16002818 | chr2 | 198651576 | *BOLL* | chr2:198649751-198651599 | Island | 0.11 | 0.004544878 |
| cg26799474 | chr2 | 202098951 | *CASP8* |  | OpenSea | -0.15 | 4.89E-06 |
| cg13372293 | chr2 | 203878778 | *NBEAL1* | chr2:203879249-203879891 | N_Shore | -0.16 | 1.33E-05 |
| cg27517968 | chr2 | 208546083 |  | chr2:208546082-208546562 | Island | -0.12 | 0.001550691 |
| cg19351166 | chr2 | 209133632 | *PIKFYVE* | chr2:209130748-209131065 | S_Shelf | -0.10 | 0.004343731 |
| cg23059946 | chr2 | 217500594 | *IGFBP2* | chr2:217497811-217498847 | S_Shore | 0.10 | 9.14E-12 |
| cg11669516 | chr2 | 217501092 | *IGFBP2* | chr2:217497811-217498847 | S_Shelf | 0.14 | 2.82E-08 |
| cg20366479 | chr2 | 217501125 | *IGFBP2* | chr2:217497811-217498847 | S_Shelf | 0.14 | 5.27E-07 |
| cg22979368 | chr2 | 217501581 | *IGFBP2* | chr2:217497811-217498847 | S_Shelf | 0.13 | 2.32E-06 |
| cg06795233 | chr2 | 219737392 | *WNT6* | chr2:219738081-219738788 | N_Shore | 0.14 | 0.000797355 |
| cg14639163 | chr2 | 219738529 | *WNT6* | chr2:219738081-219738788 | Island | 0.16 | 1.61E-06 |
| cg13903421 | chr2 | 219738714 | *WNT6* | chr2:219738081-219738788 | Island | 0.25 | 0.001143257 |
| cg03225109 | chr2 | 219773473 |  | chr2:219773546-219774103 | N_Shore | -0.11 | 4.43E-05 |
| cg18527574 | chr2 | 222434303 | *EPHA4* | chr2:222436034-222438941 | N_Shore | 0.12 | 0.003121291 |
| cg16786808 | chr2 | 222434497 | *EPHA4* | chr2:222436034-222438941 | N_Shore | 0.11 | 0.003424462 |
| cg21616047 | chr2 | 224467359 | *SCG2* |  | OpenSea | -0.15 | 0.001604689 |
| cg14931062 | chr2 | 227655596 | *IRS1* | chr2:227655903-227656647 | N_Shore | -0.13 | 4.22E-05 |
| cg03018058 | chr2 | 227700114 | *RHBDD1* | chr2:227700313-227701115 | N_Shore | -0.11 | 1.35E-05 |
| cg03727500 | chr2 | 232348334 |  | chr2:232348216-232348866 | Island | -0.25 | 8.28E-08 |
| cg05868531 | chr2 | 232348602 |  | chr2:232348216-232348866 | Island | -0.26 | 7.74E-08 |
| cg15371801 | chr2 | 232348684 |  | chr2:232348216-232348866 | Island | -0.34 | 9.76E-09 |
| cg11559198 | chr2 | 232348794 |  | chr2:232348216-232348866 | Island | -0.27 | 8.85E-08 |
| cg07301105 | chr2 | 232379665 | *C2orf52* | chr2:232378948-232379666 | Island | -0.15 | 1.86E-05 |
| cg10593047 | chr2 | 232526667 |  | chr2:232526666-232527777 | Island | -0.14 | 1.41E-06 |
| cg21124375 | chr2 | 232765392 |  | chr2:232765246-232765491 | Island | 0.10 | 0.000172721 |
| cg18427905 | chr2 | 232825958 | *DIS3L2* | chr2:232826077-232826836 | N_Shore | -0.18 | 2.60E-07 |
| cg20371266 | chr2 | 233390781 | *CHRND* | chr2:233386614-233389161 | S_Shore | 0.15 | 0.008996103 |
| cg22276371 | chr2 | 233390859 | *CHRND* | chr2:233386614-233389161 | S_Shore | 0.13 | 0.009943353 |
| cg24768561 | chr2 | 236401800 | *AGAP1* | chr2:236401799-236403946 | Island | 0.14 | 4.04E-05 |
| cg12157614 | chr2 | 236890427 | *AGAP1* |  | OpenSea | 0.18 | 0.000443958 |
| cg14226755 | chr2 | 236923322 | *AGAP1* |  | OpenSea | 0.12 | 0.001821317 |
| cg18141622 | chr2 | 238525524 |  |  | OpenSea | -0.13 | 0.009212329 |
| cg07063912 | chr2 | 238598832 | *LRRFIP1* | chr2:238599857-238601430 | N_Shore | -0.12 | 0.000698233 |
| cg08129953 | chr2 | 242448802 | *STK25* | chr2:242447017-242448558 | S_Shore | -0.12 | 5.06E-07 |
| cg07889201 | chr20 | 2490030 | *ZNF343* | chr20:2489190-2489644 | S_Shore | -0.11 | 4.16E-06 |
| cg12233487 | chr20 | 2517085 | *TMC2* |  | OpenSea | 0.11 | 0.005601219 |
| cg13518079 | chr20 | 2675072 | *EBF4* | chr20:2672904-2674698 | S_Shore | 0.36 | 6.67E-06 |
| cg05857996 | chr20 | 2675418 | *EBF4* | chr20:2672904-2674698 | S_Shore | 0.29 | 4.93E-06 |
| cg16426670 | chr20 | 2675996 | *EBF4* | chr20:2672904-2674698 | S_Shore | 0.13 | 0.000596631 |
| cg24263062 | chr20 | 2730191 | *EBF4* | chr20:2729997-2730797 | Island | 0.27 | 1.89E-05 |
| cg05825244 | chr20 | 2730488 | *EBF4* | chr20:2729997-2730797 | Island | 0.45 | 9.43E-05 |
| cg17069396 | chr20 | 2731102 | *EBF4* | chr20:2731063-2731395 | Island | 0.23 | 8.72E-08 |
| cg14959908 | chr20 | 2736884 | *EBF4* | chr20:2732746-2733630 | S_Shelf | 0.21 | 8.28E-08 |
| cg26445608 | chr20 | 2780114 | *CPXM1* | chr20:2780978-2781497 | N_Shore | -0.17 | 0.000733732 |
| cg06726390 | chr20 | 2820927 | *VPS16* | chr20:2820509-2821911 | Island | -0.17 | 5.01E-05 |
| cg07364906 | chr20 | 16710288 | *SNRPB2* | chr20:16710507-16711115 | N_Shore | -0.14 | 2.22E-09 |
| cg24899750 | chr20 | 16710314 | *SNRPB2* | chr20:16710507-16711115 | N_Shore | -0.17 | 1.01E-08 |
| cg05373875 | chr20 | 18485171 |  | chr20:18485170-18485580 | Island | -0.16 | 0.000338104 |
| cg05779219 | chr20 | 23032166 |  | chr20:23028403-23032218 | Island | -0.13 | 1.58E-07 |
| cg12037947 | chr20 | 23067752 | *CD93* |  | OpenSea | -0.11 | 0.004737074 |
| cg12873119 | chr20 | 23067771 | *CD93* |  | OpenSea | -0.13 | 0.001247749 |
| cg26787381 | chr20 | 25128777 | *LOC284798* | chr20:25128764-25129610 | Island | 0.12 | 0.001330336 |
| cg09614389 | chr20 | 25450839 | *NINL* |  | OpenSea | 0.11 | 0.001261497 |
| cg20318748 | chr20 | 25605178 | *NANP* | chr20:25603815-25605187 | Island | -0.18 | 2.79E-12 |
| cg22329555 | chr20 | 30777558 | *TSPYL3* | chr20:30777756-30778329 | N_Shore | -0.14 | 4.10E-06 |
| cg01901788 | chr20 | 33145848 | *MAP1LC3A* | chr20:33146135-33147318 | N_Shore | -0.13 | 0.00063302 |
| cg11277126 | chr20 | 33680945 | *TRPC4AP* | chr20:33680438-33681029 | Island | -0.12 | 8.15E-08 |
| cg23414001 | chr20 | 33681070 | *TRPC4AP* | chr20:33680055-33680334 | S_Shore | -0.12 | 9.95E-07 |
| cg11316100 | chr20 | 35233380 | *C20orf24* | chr20:35233829-35234782 | N_Shore | -0.10 | 0.000200413 |
| cg02142074 | chr20 | 36012596 | *SRC* | chr20:36012595-36013439 | Island | -0.13 | 1.07E-07 |
| cg20859731 | chr20 | 36226820 |  | chr20:36226617-36226841 | Island | 0.10 | 0.001046139 |
| cg03792653 | chr20 | 36661448 | *RPRD1B* | chr20:36661834-36662495 | N_Shore | -0.15 | 2.07E-06 |
| cg03539340 | chr20 | 42218758 | *IFT52* | chr20:42219516-42219988 | N_Shore | -0.12 | 0.00035729 |
| cg19931348 | chr20 | 43803224 | *PI3* |  | OpenSea | -0.20 | 0.00424147 |
| cg20015689 | chr20 | 43835190 | *SEMG1* |  | OpenSea | -0.12 | 0.001632825 |
| cg12303084 | chr20 | 45985741 | *ZMYND8* |  | OpenSea | -0.13 | 1.74E-10 |
| cg12093180 | chr20 | 49548626 | *ADNP* | chr20:49547068-49547516 | S_Shore | -0.10 | 0.003137541 |
| cg25983380 | chr20 | 57465439 | *GNAS* | chr20:57463652-57467739 | Island | -0.11 | 4.09E-05 |
| cg03264550 | chr20 | 57465448 | *GNAS* | chr20:57463652-57467739 | Island | -0.14 | 8.10E-06 |
| cg26216876 | chr20 | 57617083 | *SLMO2* | chr20:57617430-57618222 | N_Shore | -0.11 | 0.000980375 |
| cg08363339 | chr20 | 57618487 | *SLMO2* | chr20:57617430-57618222 | S_Shore | -0.16 | 0.001007634 |
| cg15677087 | chr20 | 61584850 | *SLC17A9* | chr20:61583891-61584222 | S_Shore | -0.22 | 0.004195068 |
| cg24092253 | chr20 | 61848099 | *YTHDF1* | chr20:61846843-61848103 | Island | -0.10 | 1.22E-05 |
| cg05358404 | chr20 | 62288950 | *RTEL1* | chr20:62288941-62290058 | Island | -0.16 | 0.001770074 |
| cg01355392 | chr20 | 62679255 | *SOX18* | chr20:62679424-62680883 | N_Shore | -0.17 | 0.00076084 |
| cg02231404 | chr20 | 62679635 | *SOX18* | chr20:62679424-62680883 | Island | -0.25 | 7.89E-05 |
| cg22138735 | chr20 | 62679713 | *SOX18* | chr20:62679424-62680883 | Island | -0.17 | 0.000758991 |
| cg20187011 | chr21 | 28214928 | *ADAMTS1* | chr21:28216558-28218117 | N_Shore | -0.20 | 0.000111885 |
| cg18319984 | chr21 | 30374748 |  | chr21:30374984-30375228 | N_Shore | -0.13 | 0.000101215 |
| cg20220242 | chr21 | 30392188 | *RWDD2B* | chr21:30391264-30391758 | S_Shore | -0.16 | 5.60E-05 |
| cg15920739 | chr21 | 33765737 | *URB1* | chr21:33765512-33765752 | Island | -0.10 | 0.000352558 |
| cg14172108 | chr21 | 34405553 |  | chr21:34405577-34406538 | N_Shore | 0.16 | 0.005186122 |
| cg00274965 | chr21 | 34405681 |  | chr21:34405577-34406538 | Island | 0.23 | 0.006140482 |
| cg10915739 | chr21 | 34405733 |  | chr21:34405577-34406538 | Island | 0.16 | 0.004401182 |
| cg04836472 | chr21 | 34405997 |  | chr21:34405577-34406538 | Island | 0.12 | 0.00546612 |
| cg00622702 | chr21 | 34727950 | *IFNAR1* |  | OpenSea | -0.12 | 0.007593835 |
| cg10599571 | chr21 | 35445161 | *MRPS6* | chr21:35445087-35446013 | Island | -0.14 | 6.18E-08 |
| cg06167719 | chr21 | 38362727 | *HLCS* | chr21:38362015-38362868 | Island | -0.22 | 0.004906167 |
| cg03419014 | chr21 | 38362742 | *HLCS* | chr21:38362015-38362868 | Island | -0.13 | 0.006348041 |
| cg09037712 | chr21 | 38362754 | *HLCS* | chr21:38362015-38362868 | Island | -0.11 | 0.000912745 |
| cg07599979 | chr21 | 38592612 | *DSCR9* | chr21:38592987-38593674 | N_Shore | -0.14 | 1.37E-05 |
| cg18829411 | chr21 | 40722023 | *HMGN1* | chr21:40720185-40721625 | S_Shore | -0.22 | 4.16E-09 |
| cg26390598 | chr21 | 41032396 | *B3GALT5* |  | OpenSea | -0.15 | 0.007712273 |
| cg16412370 | chr21 | 45079144 | *RRP1B* | chr21:45077671-45079821 | Island | -0.13 | 0.000349671 |
| cg11220732 | chr21 | 46065587 | *KRTAP10-11* |  | OpenSea | -0.11 | 1.89E-05 |
| cg17261675 | chr21 | 46102566 | *C21orf29* |  | OpenSea | -0.13 | 0.003794985 |
| cg13732083 | chr21 | 47605072 | *C21orf56* | chr21:47601020-47601229 | S_Shelf | 0.27 | 0.008277234 |
| cg10296238 | chr21 | 47605174 | *C21orf56* | chr21:47601020-47601229 | S_Shelf | 0.28 | 0.0052223 |
| cg25563983 | chr22 | 17517329 | *CECR7* | chr22:17516913-17518500 | Island | 0.15 | 0.001485272 |
| cg06707910 | chr22 | 17640812 | *CECR5* | chr22:17639481-17640714 | S_Shore | -0.17 | 5.72E-05 |
| cg24435209 | chr22 | 17640972 | *CECR5* | chr22:17639481-17640714 | S_Shore | -0.26 | 2.73E-07 |
| cg00227156 | chr22 | 18463646 | *MICAL3* |  | OpenSea | -0.11 | 0.000529471 |
| cg02774935 | chr22 | 22090816 | *YPEL1* | chr22:22089624-22090909 | Island | -0.12 | 0.000212043 |
| cg27653384 | chr22 | 22293118 | *PPM1F* | chr22:22292528-22292875 | S_Shore | -0.14 | 0.00070699 |
| cg21711214 | chr22 | 26875806 | *HPS4* | chr22:26879458-26879930 | N_Shelf | -0.13 | 0.000411943 |
| cg01610915 | chr22 | 29950274 | *THOC5* | chr22:29949478-29949824 | S_Shore | -0.16 | 3.43E-09 |
| cg05450667 | chr22 | 31318103 | *C22orf27* | chr22:31318228-31318489 | N_Shore | 0.11 | 0.004894872 |
| cg00930606 | chr22 | 31740944 | *PATZ1* | chr22:31741045-31743707 | N_Shore | -0.10 | 9.36E-05 |
| cg08152263 | chr22 | 35795274 | *MCM5* | chr22:35795856-35796650 | N_Shore | -0.17 | 3.55E-09 |
| cg00199549 | chr22 | 35795374 | *MCM5* | chr22:35795856-35796650 | N_Shore | -0.17 | 3.11E-09 |
| cg20162146 | chr22 | 35795394 | *MCM5* | chr22:35795856-35796650 | N_Shore | -0.11 | 5.31E-07 |
| cg03992976 | chr22 | 36903496 | *FOXRED2* | chr22:36902024-36903298 | S_Shore | -0.13 | 0.000187527 |
| cg07141002 | chr22 | 38201690 | *H1F0* | chr22:38200672-38201691 | Island | -0.11 | 0.002731989 |
| cg07059402 | chr22 | 38244781 | *MIR659* | chr22:38245341-38245681 | N_Shore | -0.14 | 0.001129475 |
| cg03131358 | chr22 | 42195972 | *CCDC134* | chr22:42195953-42196961 | Island | -0.11 | 0.000994432 |
| cg09667013 | chr22 | 42394590 | *WBP2NL* | chr22:42394589-42395255 | Island | -0.13 | 0.001672685 |
| cg04717802 | chr22 | 42394638 | *WBP2NL* | chr22:42394589-42395255 | Island | -0.11 | 0.002097436 |
| cg01059385 | chr22 | 42394853 | *WBP2NL* | chr22:42394589-42395255 | Island | -0.16 | 0.000632548 |
| cg22189786 | chr22 | 42395067 | *WBP2NL* | chr22:42394589-42395255 | Island | -0.25 | 5.18E-05 |
| cg16740427 | chr22 | 42469976 | *FAM109B* | chr22:42470035-42470669 | N_Shore | -0.21 | 1.62E-09 |
| cg24977886 | chr22 | 42470101 | *FAM109B* | chr22:42470035-42470669 | Island | -0.19 | 2.82E-08 |
| cg08057985 | chr22 | 42470123 | *FAM109B* | chr22:42470035-42470669 | Island | -0.14 | 1.05E-07 |
| cg18034295 | chr22 | 42475135 | *C22orf32* | chr22:42475474-42476094 | N_Shore | -0.12 | 0.000144383 |
| cg20727362 | chr22 | 43045952 | *CYB5R3* | chr22:43045009-43045679 | S_Shore | -0.12 | 0.004216422 |
| cg02233750 | chr22 | 43539658 | *MCAT* | chr22:43538913-43539526 | S_Shore | -0.19 | 0.001000743 |
| cg15477144 | chr22 | 45705034 | *FAM118A* | chr22:45705511-45706616 | N_Shore | -0.20 | 0.004542989 |
| cg00643333 | chr22 | 45705037 | *FAM118A* | chr22:45705511-45706616 | N_Shore | -0.19 | 0.003456823 |
| cg22160612 | chr22 | 45705042 | *FAM118A* | chr22:45705511-45706616 | N_Shore | -0.15 | 0.002682314 |
| cg05742863 | chr22 | 45809319 | *SMC1B* | chr22:45809191-45809953 | Island | -0.15 | 0.00024209 |
| cg09491104 | chr22 | 46646882 | *C22orf40* | chr22:46645764-46646957 | Island | -0.14 | 6.35E-06 |
| cg20744362 | chr22 | 50050164 | *C22orf34* |  | OpenSea | -0.35 | 0.00012917 |
| cg02776313 | chr22 | 50965782 | *TYMP* | chr22:50963608-50970768 | Island | -0.19 | 0.000526476 |
| cg10416593 | chr22 | 50966123 | *TYMP* | chr22:50963608-50970768 | Island | -0.14 | 0.00456422 |
| cg10990081 | chr3 | 3168142 | *TRNT1* | chr3:3168324-3169231 | N_Shore | -0.14 | 1.91E-07 |
| cg05253480 | chr3 | 9944537 | *IL17RE* |  | OpenSea | -0.11 | 0.006058068 |
| cg02968508 | chr3 | 9956698 | *IL17RE* | chr3:9956822-9957664 | N_Shore | -0.13 | 0.00058677 |
| cg01142302 | chr3 | 9988590 | *PRRT3* | chr3:9987895-9989619 | Island | -0.20 | 0.000932834 |
| cg05851542 | chr3 | 9989156 | *PRRT3* | chr3:9987895-9989619 | Island | -0.12 | 0.000549741 |
| cg14583999 | chr3 | 10019040 | *TMEM111* |  | OpenSea | -0.14 | 0.006834773 |
| cg19764594 | chr3 | 10806288 | *LOC285370* |  | OpenSea | 0.10 | 0.000211968 |
| cg00384701 | chr3 | 13974800 |  | chr3:13974400-13974867 | Island | -0.13 | 1.28E-06 |
| ch.3.574787R | chr3 | 27578816 |  |  | OpenSea | -0.11 | 0.001245959 |
| cg18480675 | chr3 | 28281945 | *CMC1* | chr3:28282826-28283457 | N_Shore | -0.12 | 0.00250326 |
| cg13679679 | chr3 | 28282245 | *CMC1* | chr3:28282826-28283457 | N_Shore | -0.13 | 0.003519078 |
| cg13050802 | chr3 | 32509039 |  | chr3:32509265-32509525 | N_Shore | -0.16 | 0.003658724 |
| cg24607398 | chr3 | 37033625 | *EPM2AIP1* | chr3:37034228-37035356 | N_Shore | -0.10 | 0.003670635 |
| cg04726821 | chr3 | 37033791 | *MLH1* | chr3:37034228-37035356 | N_Shore | -0.11 | 0.000927638 |
| cg12984086 | chr3 | 37284287 | *GOLGA4* | chr3:37284558-37285281 | N_Shore | -0.15 | 0.000710852 |
| cg05620558 | chr3 | 38040073 | *VILL* | chr3:38040289-38040535 | N_Shore | -0.11 | 0.007224433 |
| cg12340144 | chr3 | 38388808 | *XYLB* | chr3:38388122-38388769 | S_Shore | -0.12 | 7.61E-05 |
| cg14870223 | chr3 | 39093076 | *WDR48* | chr3:39093469-39094025 | N_Shore | -0.21 | 1.25E-11 |
| cg17621718 | chr3 | 42306737 | *CCK* | chr3:42306149-42307520 | Island | 0.10 | 0.000992457 |
| cg25493589 | chr3 | 42307193 | *CCK* | chr3:42306149-42307520 | Island | 0.10 | 0.000218446 |
| cg25516803 | chr3 | 42307519 | *CCK* | chr3:42306149-42307520 | Island | 0.14 | 0.000127497 |
| cg23009419 | chr3 | 46618597 | *LRRC2* | chr3:46618307-46618669 | Island | 0.11 | 0.001188113 |
| cg04035377 | chr3 | 48693119 | *CELSR3* | chr3:48693118-48694768 | Island | -0.10 | 3.36E-06 |
| cg01919208 | chr3 | 49170496 | *LAMB2* |  | OpenSea | -0.13 | 0.003466877 |
| cg02954987 | chr3 | 49170599 | *LAMB2* |  | OpenSea | -0.17 | 0.006224832 |
| cg08234664 | chr3 | 49170668 | *LAMB2* |  | OpenSea | -0.22 | 0.000863723 |
| cg05654765 | chr3 | 49170727 | *LAMB2* |  | OpenSea | -0.14 | 0.000401232 |
| cg14099457 | chr3 | 49170794 | *LAMB2* |  | OpenSea | -0.18 | 0.000553887 |
| cg11566975 | chr3 | 49170849 | *LAMB2* |  | OpenSea | -0.11 | 0.002065306 |
| cg18605031 | chr3 | 49314155 | *C3orf62* | chr3:49314437-49314815 | N_Shore | -0.24 | 1.04E-16 |
| cg25090325 | chr3 | 49755764 | *AMIGO3* | chr3:49755763-49757317 | Island | -0.12 | 0.000192415 |
| cg22993195 | chr3 | 49756301 | *AMIGO3* | chr3:49755763-49757317 | Island | -0.29 | 1.80E-07 |
| cg20119308 | chr3 | 50376000 | *RASSF1* | chr3:50377803-50378540 | N_Shore | -0.20 | 1.13E-06 |
| cg05546296 | chr3 | 50376006 | *RASSF1* | chr3:50377803-50378540 | N_Shore | -0.22 | 4.69E-09 |
| cg19854901 | chr3 | 50376216 | *RASSF1* | chr3:50377803-50378540 | N_Shore | -0.18 | 1.64E-08 |
| cg23431721 | chr3 | 50387780 | *TUSC4* | chr3:50387959-50388681 | N_Shore | -0.13 | 7.06E-07 |
| cg02857273 | chr3 | 57541377 | *PDE12* | chr3:57541931-57543244 | N_Shore | -0.11 | 0.000154924 |
| cg06028917 | chr3 | 59035407 | *C3orf67* | chr3:59035406-59035944 | Island | -0.11 | 0.000325897 |
| cg07212940 | chr3 | 62304165 | *C3orf14* | chr3:62304514-62304780 | N_Shore | -0.13 | 0.000248272 |
| cg10549088 | chr3 | 64277154 |  |  | OpenSea | -0.12 | 0.006002134 |
| cg09439920 | chr3 | 99979117 | *TBC1D23* | chr3:99979678-99980038 | N_Shore | -0.21 | 6.17E-09 |
| cg04730047 | chr3 | 99979355 | *TBC1D23* | chr3:99979678-99980038 | N_Shore | -0.16 | 2.29E-09 |
| cg16984944 | chr3 | 99979425 | *TBC1D23* | chr3:99979678-99980038 | N_Shore | -0.12 | 2.21E-07 |
| cg18745424 | chr3 | 100401200 | *GPR128* |  | OpenSea | -0.11 | 0.002473785 |
| cg00186462 | chr3 | 100401284 | *GPR128* |  | OpenSea | -0.15 | 0.002092147 |
| cg08889687 | chr3 | 110789300 |  | chr3:110790149-110791401 | N_Shore | 0.12 | 0.00233405 |
| cg23198707 | chr3 | 112023284 |  |  | OpenSea | -0.17 | 0.000558976 |
| cg13537646 | chr3 | 112143970 |  |  | OpenSea | -0.13 | 0.005862126 |
| cg02905245 | chr3 | 112359652 | *CCDC80* |  | OpenSea | 0.10 | 0.000421022 |
| cg12151328 | chr3 | 112709521 | *GTPBP8* | chr3:112709666-112710270 | N_Shore | -0.17 | 7.87E-06 |
| cg17228765 | chr3 | 112738800 | *C3orf17* | chr3:112738244-112738653 | S_Shore | -0.14 | 4.97E-06 |
| cg07804735 | chr3 | 113157476 | *WDR52* | chr3:113160299-113160641 | N_Shelf | 0.12 | 0.000197166 |
| cg18753928 | chr3 | 113234510 | *CCDC52* | chr3:113233663-113234094 | S_Shore | -0.11 | 0.007317611 |
| cg07890553 | chr3 | 119182858 | *TMEM39A* |  | OpenSea | -0.11 | 1.46E-06 |
| cg20696605 | chr3 | 119187426 | *KTELC1* | chr3:119187747-119188308 | N_Shore | -0.20 | 2.56E-06 |
| cg17421623 | chr3 | 119187570 | *KTELC1* | chr3:119187747-119188308 | N_Shore | -0.14 | 5.51E-05 |
| cg24846416 | chr3 | 119529290 | *NR1I2* | chr3:119528920-119529159 | S_Shore | -0.11 | 0.000533624 |
| cg25260865 | chr3 | 124932002 | *SLC12A8* | chr3:124931161-124931747 | S_Shore | -0.11 | 1.78E-05 |
| cg03438417 | chr3 | 126007325 |  | chr3:126007324-126007527 | Island | 0.11 | 0.000901354 |
| cg11830761 | chr3 | 126007485 |  | chr3:126007324-126007527 | Island | 0.12 | 0.000185412 |
| cg21487550 | chr3 | 127056955 |  | chr3:127056836-127057296 | Island | 0.12 | 0.007986147 |
| cg25436886 | chr3 | 127056972 |  | chr3:127056836-127057296 | Island | 0.12 | 0.003284484 |
| cg20920097 | chr3 | 127057149 |  | chr3:127056836-127057296 | Island | 0.11 | 0.001204942 |
| cg17324149 | chr3 | 127725522 |  |  | OpenSea | 0.10 | 0.00269417 |
| cg18303397 | chr3 | 129160135 | *IFT122* | chr3:129158602-129159477 | S_Shore | -0.11 | 0.000683098 |
| cg15484384 | chr3 | 134203672 | *ANAPC13* | chr3:134204520-134205439 | N_Shore | -0.10 | 0.00096419 |
| cg10109841 | chr3 | 135969958 | *PCCB* | chr3:135969040-135969565 | S_Shore | -0.12 | 4.25E-06 |
| cg06137072 | chr3 | 141087187 | *ZBTB38* |  | OpenSea | -0.13 | 0.000831475 |
| cg08360599 | chr3 | 141087261 | *ZBTB38* |  | OpenSea | -0.13 | 0.001936105 |
| cg21370924 | chr3 | 141087313 | *ZBTB38* |  | OpenSea | -0.16 | 0.000720268 |
| cg21474062 | chr3 | 141087363 | *ZBTB38* |  | OpenSea | -0.16 | 0.006297916 |
| cg02802029 | chr3 | 145879686 | *PLOD2* | chr3:145878430-145879287 | S_Shore | -0.12 | 0.007675066 |
| cg06573459 | chr3 | 153840654 | *SGEF* | chr3:153838787-153840380 | S_Shore | 0.12 | 0.002290079 |
| cg08832018 | chr3 | 156543647 | *LEKR1* | chr3:156543891-156544405 | N_Shore | -0.11 | 0.006379487 |
| cg26331343 | chr3 | 156807520 |  | chr3:156806688-156807069 | S_Shore | -0.11 | 0.003241518 |
| cg20892840 | chr3 | 156879058 | *CCNL1* | chr3:156877175-156878364 | S_Shore | -0.17 | 1.37E-05 |
| cg05725721 | chr3 | 160823387 | *B3GALNT1* | chr3:160822494-160823260 | S_Shore | -0.11 | 0.000993992 |
| cg12927753 | chr3 | 160823392 | *B3GALNT1* | chr3:160822494-160823260 | S_Shore | -0.12 | 0.003241204 |
| cg14553504 | chr3 | 169898657 | *PHC3* | chr3:169898946-169899626 | N_Shore | -0.19 | 2.95E-06 |
| cg02694620 | chr3 | 172109284 | *FNDC3B* |  | OpenSea | -0.12 | 0.002865335 |
| cg15721584 | chr3 | 181326755 | *SOX2OT* |  | OpenSea | -0.18 | 0.002523296 |
| cg11729363 | chr3 | 183271175 | *KLHL6* |  | OpenSea | -0.10 | 0.00034986 |
| cg13491563 | chr3 | 183903302 | *ABCF3* | chr3:183903386-183904495 | N_Shore | -0.10 | 2.36E-05 |
| cg09866366 | chr3 | 183903315 | *ABCF3* | chr3:183903386-183904495 | N_Shore | -0.15 | 1.62E-06 |
| cg26152983 | chr3 | 184428654 | *MAGEF1* | chr3:184429366-184429907 | N_Shore | -0.15 | 2.32E-05 |
| cg15487251 | chr3 | 185544216 | *IGF2BP2* | chr3:185541048-185544146 | S_Shore | -0.10 | 0.008154794 |
| cg07693270 | chr3 | 186856928 | *RPL39L* | chr3:186856927-186857659 | Island | -0.11 | 0.000363214 |
| cg27312961 | chr3 | 191048361 | *UTS2D* | chr3:191046948-191047710 | S_Shore | -0.11 | 0.007395135 |
| cg01526748 | chr3 | 191930926 | *FGF12* |  | OpenSea | -0.12 | 0.006416555 |
| cg04134015 | chr3 | 193310532 | *OPA1* | chr3:193310824-193311188 | N_Shore | -0.12 | 0.000655554 |
| cg03456393 | chr3 | 193310565 | *OPA1* | chr3:193310824-193311188 | N_Shore | -0.16 | 5.06E-07 |
| cg03765423 | chr3 | 194014481 |  | chr3:194014480-194014983 | Island | -0.13 | 8.56E-05 |
| cg02153814 | chr3 | 194014525 |  | chr3:194014480-194014983 | Island | -0.21 | 3.20E-06 |
| cg25661792 | chr3 | 194014530 |  | chr3:194014480-194014983 | Island | -0.18 | 1.83E-06 |
| cg10663765 | chr3 | 194014592 |  | chr3:194014480-194014983 | Island | -0.16 | 0.000730302 |
| cg11324650 | chr3 | 194014745 |  | chr3:194014480-194014983 | Island | -0.14 | 0.000191721 |
| cg08991643 | chr3 | 194014928 |  | chr3:194014480-194014983 | Island | -0.14 | 0.000991917 |
| cg18766468 | chr3 | 194090477 | *LRRC15* |  | OpenSea | 0.11 | 0.003219155 |
| cg12681948 | chr3 | 194208784 |  | chr3:194207385-194208785 | Island | -0.15 | 0.004099466 |
| cg08877188 | chr3 | 195943579 | *OSTalpha* |  | OpenSea | 0.13 | 0.000286421 |
| cg05674437 | chr3 | 196694153 | *PIGZ* | chr3:196693765-196694200 | Island | 0.15 | 0.00025309 |
| cg19280206 | chr3 | 196694932 | *PIGZ* | chr3:196695319-196697065 | N_Shore | 0.17 | 0.003391279 |
| cg01947751 | chr3 | 196728969 |  | chr3:196728958-196730878 | Island | -0.15 | 5.71E-06 |
| cg20743744 | chr4 | 1243849 | *C4orf42* | chr4:1241412-1244111 | Island | -0.14 | 1.02E-06 |
| cg26479374 | chr4 | 1243980 | *CTBP1* | chr4:1241412-1244111 | Island | -0.27 | 0.007022419 |
| cg15586393 | chr4 | 1244086 | *CTBP1* | chr4:1241412-1244111 | Island | -0.16 | 0.00160563 |
| cg03150409 | chr4 | 1892317 | *WHSC1* |  | OpenSea | 0.14 | 0.003411625 |
| cg23855319 | chr4 | 2062392 | *NAT8L* | chr4:2059928-2063181 | Island | 0.26 | 0.000150847 |
| cg10432947 | chr4 | 2062441 | *NAT8L* | chr4:2059928-2063181 | Island | 0.22 | 0.0001105 |
| cg18089397 | chr4 | 2264747 | *MXD4* | chr4:2262960-2264669 | S_Shore | -0.13 | 3.65E-05 |
| cg01290904 | chr4 | 5708474 | *EVC2* | chr4:5709985-5710495 | N_Shore | -0.19 | 0.008685036 |
| cg15305511 | chr4 | 5712676 | *EVC2* | chr4:5713035-5713451 | N_Shore | -0.11 | 0.000292164 |
| cg14107488 | chr4 | 7033722 | *TBC1D14* |  | OpenSea | -0.17 | 0.001308749 |
| cg09907542 | chr4 | 7033761 | *TBC1D14* |  | OpenSea | -0.17 | 0.003977103 |
| cg00123478 | chr4 | 10458165 | *ZNF518B* | chr4:10458129-10459353 | Island | -0.12 | 3.90E-05 |
| cg11511795 | chr4 | 10463749 |  | chr4:10462832-10463689 | S_Shore | 0.14 | 7.57E-05 |
| cg15175162 | chr4 | 15657657 | *FBXL5* | chr4:15656364-15657739 | Island | -0.42 | 7.33E-17 |
| cg18099096 | chr4 | 23936764 |  |  | OpenSea | -0.21 | 0.00277776 |
| cg04349839 | chr4 | 25789390 | *SEL1L3* |  | OpenSea | -0.12 | 0.003629644 |
| cg24438217 | chr4 | 39980114 | *PDS5A* | chr4:39978066-39979792 | S_Shore | -0.17 | 0.001955854 |
| cg01166925 | chr4 | 41880280 |  | chr4:41880224-41880500 | Island | 0.12 | 0.002355855 |
| cg16091553 | chr4 | 46126245 | *GABRG1* |  | OpenSea | 0.23 | 0.009322173 |
| cg10021428 | chr4 | 53588038 |  | chr4:53588189-53588735 | N_Shore | -0.11 | 0.006140482 |
| cg06483432 | chr4 | 55523233 | *KIT* | chr4:55523409-55525297 | N_Shore | -0.14 | 0.00163618 |
| cg19676181 | chr4 | 56660398 |  | chr4:56659626-56660144 | S_Shore | 0.12 | 0.000724095 |
| ch.4.1172045R | chr4 | 57343548 | *SRP72* |  | OpenSea | -0.12 | 0.001417858 |
| cg07974833 | chr4 | 73430160 | *ADAMTS3* | chr4:73433783-73434289 | N_Shelf | -0.18 | 0.003424462 |
| cg08572214 | chr4 | 73936052 | *COX18* | chr4:73934944-73935650 | S_Shore | -0.23 | 9.56E-06 |
| cg07868155 | chr4 | 74864709 | *CXCL5* | chr4:74864113-74864329 | S_Shore | -0.10 | 0.000452775 |
| cg03475509 | chr4 | 85402143 |  | chr4:85402764-85403175 | N_Shore | -0.11 | 0.007571062 |
| cg03676636 | chr4 | 99064102 | *C4orf37* | chr4:99064602-99064805 | N_Shore | -0.18 | 0.000363214 |
| cg14972143 | chr4 | 99851003 | *EIF4E* | chr4:99849305-99850552 | S_Shore | -0.12 | 3.23E-06 |
| cg11037477 | chr4 | 99851008 | *EIF4E* | chr4:99849305-99850552 | S_Shore | -0.13 | 8.15E-06 |
| cg18160135 | chr4 | 99851115 | *EIF4E* | chr4:99849305-99850552 | S_Shore | -0.11 | 2.03E-06 |
| cg15633390 | chr4 | 99851211 | *EIF4E* | chr4:99849305-99850552 | S_Shore | -0.13 | 9.99E-05 |
| cg02193956 | chr4 | 103680201 | *MANBA* | chr4:103681836-103682348 | N_Shore | -0.15 | 0.008486082 |
| cg23196549 | chr4 | 110480700 | *CCDC109B* | chr4:110480827-110482060 | N_Shore | -0.13 | 1.95E-05 |
| cg17718401 | chr4 | 119199352 | *SNORA24* | chr4:119199620-119200193 | N_Shore | -0.16 | 4.31E-05 |
| cg06713675 | chr4 | 122721982 | *EXOSC9* | chr4:122722335-122722758 | N_Shore | -0.26 | 0.005945292 |
| cg18236571 | chr4 | 135122410 | *PABPC4L* |  | OpenSea | -0.17 | 3.31E-06 |
| cg14497545 | chr4 | 140754475 | *MAML3* |  | OpenSea | 0.11 | 0.002968612 |
| cg02188818 | chr4 | 141676849 | *TBC1D9* | chr4:141676920-141678170 | N_Shore | -0.11 | 0.00034804 |
| cg20212912 | chr4 | 147557774 |  | chr4:147558231-147558583 | N_Shore | -0.11 | 0.003201432 |
| cg19935951 | chr4 | 151258915 | *LRBA* |  | OpenSea | -0.13 | 0.007466778 |
| cg15736994 | chr4 | 156775941 | *ACCN5* |  | OpenSea | -0.12 | 0.005936372 |
| cg21401642 | chr4 | 174421114 |  | chr4:174421347-174421559 | N_Shore | 0.13 | 6.81E-06 |
| cg08814800 | chr4 | 174421377 |  | chr4:174421347-174421559 | Island | 0.13 | 1.03E-06 |
| cg12519676 | chr4 | 174912210 |  |  | OpenSea | 0.15 | 0.006262771 |
| cg21026199 | chr4 | 174912278 |  |  | OpenSea | 0.15 | 0.005889566 |
| cg10059484 | chr4 | 183839326 | *DCTD* | chr4:183838036-183839286 | S_Shore | -0.10 | 2.95E-10 |
| cg21490662 | chr4 | 185571520 | *CASP3* | chr4:185569961-185570638 | S_Shore | -0.12 | 0.006051439 |
| cg12167823 | chr4 | 185571549 | *CASP3* | chr4:185569961-185570638 | S_Shore | -0.18 | 0.000668482 |
| cg02012338 | chr4 | 187126139 | *CYP4V2* |  | OpenSea | -0.13 | 0.00271534 |
| cg08288016 | chr4 | 187590536 | *FAT1* |  | OpenSea | 0.14 | 0.00336913 |
| cg24716416 | chr4 | 188736112 |  |  | OpenSea | -0.21 | 0.000488943 |
| cg20070659 | chr4 | 188736336 |  |  | OpenSea | -0.15 | 0.001699238 |
| cg19969650 | chr5 | 1634502 | *LOC728613* | chr5:1633901-1634224 | S_Shore | -0.16 | 0.000780294 |
| cg24139639 | chr5 | 3605884 |  | chr5:3606488-3607135 | N_Shore | 0.14 | 1.44E-05 |
| cg17298925 | chr5 | 6828054 |  |  | OpenSea | -0.13 | 0.003063819 |
| cg26330063 | chr5 | 14614735 | *FAM105A* |  | OpenSea | 0.12 | 0.000610055 |
| cg04362002 | chr5 | 23506738 | *PRDM9* |  | OpenSea | -0.12 | 0.001753765 |
| cg25472530 | chr5 | 23507617 | *PRDM9* |  | OpenSea | -0.17 | 0.005023782 |
| cg22079902 | chr5 | 23507644 | *PRDM9* |  | OpenSea | -0.19 | 0.001007935 |
| cg01667892 | chr5 | 23507656 | *PRDM9* |  | OpenSea | -0.20 | 0.002282234 |
| cg08657705 | chr5 | 30662916 |  |  | OpenSea | -0.10 | 0.000914747 |
| cg18198212 | chr5 | 39455917 |  |  | OpenSea | 0.10 | 0.007570132 |
| cg17351974 | chr5 | 40835760 | *RPL37* | chr5:40835241-40835873 | Island | -0.19 | 4.16E-06 |
| cg01025774 | chr5 | 42944457 |  | chr5:42944266-42944582 | Island | 0.12 | 0.000285799 |
| cg20929922 | chr5 | 43484725 | *C5orf28* | chr5:43483519-43484555 | S_Shore | -0.11 | 0.000265625 |
| cg17814758 | chr5 | 45192536 |  |  | OpenSea | -0.12 | 0.002368496 |
| cg17223170 | chr5 | 50468642 |  |  | OpenSea | -0.11 | 0.000170077 |
| cg16619991 | chr5 | 52096811 | *ITGA1* | chr5:52095811-52096025 | S_Shore | -0.18 | 3.38E-06 |
| cg12351310 | chr5 | 52937992 | *NDUFS4* |  | OpenSea | -0.14 | 0.00167278 |
| cg00992687 | chr5 | 54275155 | *ESM1* |  | OpenSea | -0.14 | 0.006262771 |
| cg13028113 | chr5 | 60458778 | *C5orf43* | chr5:60457840-60458654 | S_Shore | -0.15 | 7.59E-08 |
| cg09645336 | chr5 | 60458807 | *C5orf43* | chr5:60457840-60458654 | S_Shore | -0.17 | 1.44E-07 |
| cg09134760 | chr5 | 60921084 |  | chr5:60921534-60922472 | N_Shore | -0.12 | 0.00691806 |
| cg06294700 | chr5 | 64330959 |  | chr5:64331232-64331795 | N_Shore | -0.14 | 1.42E-07 |
| cg03883572 | chr5 | 66461884 | *MAST4* | chr5:66459035-66459595 | S_Shelf | -0.15 | 0.001539545 |
| cg17278401 | chr5 | 66462662 | *MAST4* | chr5:66459035-66459595 | S_Shelf | -0.12 | 0.001948028 |
| cg02580917 | chr5 | 68628240 |  | chr5:68628422-68628739 | N_Shore | -0.13 | 0.0075286 |
| cg21626163 | chr5 | 70882722 | *MCCC2* | chr5:70882936-70883413 | N_Shore | -0.12 | 6.08E-08 |
| cg10441379 | chr5 | 72793693 | *BTF3* | chr5:72794040-72794641 | N_Shore | -0.28 | 6.85E-07 |
| cg07222505 | chr5 | 74161231 | *FAM169A* | chr5:74161536-74162716 | N_Shore | 0.13 | 0.008982952 |
| cg22935653 | chr5 | 74532268 | *ANKRD31* | chr5:74532626-74532899 | N_Shore | -0.16 | 1.00E-10 |
| cg00165981 | chr5 | 74808039 | *COL4A3BP* | chr5:74806581-74807974 | S_Shore | -0.11 | 0.000100531 |
| cg22202786 | chr5 | 76383494 | *LOC728723* | chr5:76382727-76383167 | S_Shore | -0.12 | 1.28E-09 |
| cg07183876 | chr5 | 80257268 | *RASGRF2* | chr5:80255904-80257006 | S_Shore | 0.12 | 0.000154781 |
| cg17330048 | chr5 | 80257322 | *RASGRF2* | chr5:80255904-80257006 | S_Shore | 0.13 | 7.41E-06 |
| cg18783429 | chr5 | 92414398 |  |  | OpenSea | -0.12 | 0.005443478 |
| cg27096298 | chr5 | 92904875 | *FLJ42709* | chr5:92906239-92908875 | N_Shore | 0.10 | 0.002826682 |
| cg12950181 | chr5 | 93447648 | *FAM172A* | chr5:93446953-93447436 | S_Shore | -0.13 | 1.16E-06 |
| cg17253517 | chr5 | 93447700 | *FAM172A* | chr5:93446953-93447436 | S_Shore | -0.11 | 0.003208963 |
| cg15203566 | chr5 | 115697446 |  | chr5:115697134-115697589 | Island | -0.20 | 0.006506142 |
| cg01145903 | chr5 | 115697588 |  | chr5:115697134-115697589 | Island | -0.13 | 0.00365684 |
| cg12532266 | chr5 | 132201652 | *GDF9* | chr5:132201901-132202753 | N_Shore | -0.13 | 3.65E-05 |
| cg11250576 | chr5 | 132386346 | *HSPA4* | chr5:132387100-132388369 | N_Shore | -0.11 | 1.15E-05 |
| cg01952185 | chr5 | 134813213 |  |  | OpenSea | 0.13 | 0.002866099 |
| cg11429111 | chr5 | 134813329 |  |  | OpenSea | 0.11 | 0.001206565 |
| cg02250764 | chr5 | 134878149 |  | chr5:134879443-134879819 | N_Shore | 0.12 | 0.00126887 |
| cg26817546 | chr5 | 135538704 |  |  | OpenSea | 0.13 | 0.002374829 |
| cg17275074 | chr5 | 135701422 | *TRPC7* |  | OpenSea | -0.11 | 0.003019712 |
| cg12414301 | chr5 | 137224213 | *PKD2L2* | chr5:137224986-137225477 | N_Shore | -0.19 | 0.000462812 |
| cg10535132 | chr5 | 137224284 | *PKD2L2* | chr5:137224986-137225477 | N_Shore | -0.22 | 1.18E-05 |
| cg00373499 | chr5 | 137224723 | *PKD2L2* | chr5:137224986-137225477 | N_Shore | -0.18 | 2.57E-07 |
| cg05237436 | chr5 | 138533428 | *SIL1* | chr5:138533870-138534113 | N_Shore | -0.31 | 2.21E-14 |
| cg06660015 | chr5 | 139743303 | *SLC4A9* | chr5:139742899-139743148 | S_Shore | 0.12 | 0.003685788 |
| cg25340050 | chr5 | 140562562 | *PCDHB16* | chr5:140563510-140564361 | N_Shore | -0.15 | 0.009475943 |
| cg18781988 | chr5 | 140723549 | *PCDHGA2* | chr5:140723548-140723900 | Island | 0.12 | 0.00138994 |
| cg21915313 | chr5 | 140774426 | *PCDHGA4* | chr5:140773855-140774441 | Island | 0.13 | 0.001006206 |
| cg02707176 | chr5 | 140798758 | *PCDHGA4* | chr5:140798757-140799359 | Island | 0.15 | 1.60E-05 |
| cg02452944 | chr5 | 140810109 | *PCDHGA4* | chr5:140810494-140812617 | N_Shore | 0.11 | 9.65E-05 |
| cg07730329 | chr5 | 140810137 | *PCDHGA4* | chr5:140810494-140812617 | N_Shore | 0.11 | 0.000234806 |
| cg11830096 | chr5 | 140865433 | *PCDHGA4* | chr5:140864527-140864748 | S_Shore | 0.11 | 0.000225522 |
| cg09546802 | chr5 | 145215546 | *PRELID2* | chr5:145214649-145215139 | S_Shore | -0.18 | 1.24E-05 |
| cg11609571 | chr5 | 145215629 | *PRELID2* | chr5:145214649-145215139 | S_Shore | -0.21 | 0.000492399 |
| cg26588194 | chr5 | 149379518 | *HMGXB3* | chr5:149379964-149380802 | N_Shore | -0.10 | 4.24E-06 |
| cg15699693 | chr5 | 150054944 | *MYOZ3* | chr5:150051116-150052107 | S_Shelf | -0.15 | 0.008021304 |
| cg12924095 | chr5 | 151150029 | *G3BP1* | chr5:151150014-151152086 | Island | -0.29 | 8.13E-13 |
| cg08414643 | chr5 | 153873149 |  | chr5:153872839-153873228 | Island | 0.13 | 0.003424462 |
| cg07371521 | chr5 | 154026371 |  | chr5:154026818-154027257 | N_Shore | -0.13 | 0.005893825 |
| cg00594129 | chr5 | 158524270 | *EBF1* | chr5:158523906-158524598 | Island | -0.14 | 0.001548894 |
| cg02884181 | chr5 | 158689508 | *UBLCP1* | chr5:158690013-158690541 | N_Shore | -0.20 | 0.001136704 |
| cg16391973 | chr5 | 158689566 | *UBLCP1* | chr5:158690013-158690541 | N_Shore | -0.14 | 0.005665898 |
| cg07004950 | chr5 | 159508665 | *PWWP2A* |  | OpenSea | -0.11 | 0.00562233 |
| cg17334359 | chr5 | 162929830 | *MAT2B* | chr5:162932454-162932946 | N_Shelf | -0.10 | 0.007466778 |
| cg11144555 | chr5 | 163723934 |  |  | OpenSea | 0.12 | 0.009198853 |
| cg08765317 | chr5 | 167719742 | *WWC1* | chr5:167718523-167719688 | S_Shore | 0.10 | 0.006379487 |
| cg08477779 | chr5 | 168007137 | *PANK3* | chr5:168006098-168006918 | S_Shore | -0.10 | 2.05E-05 |
| cg16046214 | chr5 | 170289848 | *RANBP17* | chr5:170288879-170289737 | S_Shore | -0.13 | 1.30E-05 |
| cg07140792 | chr5 | 171165686 |  |  | OpenSea | -0.11 | 0.003503874 |
| cg18040788 | chr5 | 173852421 |  |  | OpenSea | -0.11 | 3.69E-05 |
| cg22807681 | chr5 | 174622933 |  |  | OpenSea | 0.12 | 0.000567007 |
| cg11813441 | chr5 | 176543790 |  | chr5:176543900-176544228 | N_Shore | -0.12 | 3.66E-05 |
| cg23476877 | chr5 | 176543964 |  | chr5:176543900-176544228 | Island | -0.12 | 0.003424462 |
| cg19731612 | chr5 | 176559334 | *NSD1* | chr5:176558852-176561652 | Island | -0.31 | 4.74E-07 |
| cg17493885 | chr5 | 176559558 | *NSD1* | chr5:176558852-176561652 | Island | -0.32 | 6.04E-09 |
| cg18121224 | chr5 | 176559563 | *NSD1* | chr5:176558852-176561652 | Island | -0.35 | 2.98E-10 |
| cg17811323 | chr5 | 179004707 | *RUFY1* | chr5:179004497-179004836 | Island | 0.12 | 0.006080132 |
| cg08962798 | chr5 | 179106333 | *CBY3* | chr5:179105169-179106412 | Island | -0.17 | 1.98E-11 |
| cg03671052 | chr5 | 180086260 |  |  | OpenSea | 0.14 | 0.001303217 |
| cg25332357 | chr5 | 180238296 | *MGAT1* | chr5:180236992-180238209 | S_Shore | -0.14 | 4.80E-09 |
| cg21127580 | chr6 | 901081 |  |  | OpenSea | -0.12 | 0.000188267 |
| cg16431787 | chr6 | 1082220 | *LOC285768* |  | OpenSea | -0.14 | 0.003932332 |
| cg09113474 | chr6 | 1601369 |  | chr6:1601624-1602138 | N_Shore | 0.11 | 0.003439239 |
| cg21010202 | chr6 | 1615843 |  | chr6:1604606-1615866 | Island | 0.13 | 0.004132342 |
| cg07905696 | chr6 | 5026164 |  |  | OpenSea | -0.14 | 5.72E-07 |
| cg09548084 | chr6 | 8436218 | *SLC35B3* | chr6:8435314-8436141 | S_Shore | -0.21 | 0.004285129 |
| cg02152351 | chr6 | 8436296 | *SLC35B3* | chr6:8435314-8436141 | S_Shore | -0.13 | 0.006244945 |
| cg20892245 | chr6 | 15244618 |  | chr6:15244877-15246160 | N_Shore | -0.14 | 0.005704793 |
| cg15210999 | chr6 | 15663538 | *DTNBP1* | chr6:15662444-15663412 | S_Shore | -0.14 | 0.000133098 |
| cg05065239 | chr6 | 18990650 |  |  | OpenSea | 0.11 | 2.89E-05 |
| cg06480265 | chr6 | 24722060 |  | chr6:24720387-24721920 | S_Shore | -0.20 | 1.09E-10 |
| cg27583138 | chr6 | 24774877 | *GMNN* | chr6:24775046-24775729 | N_Shore | -0.27 | 2.76E-08 |
| cg26174326 | chr6 | 26016674 |  | chr6:26020671-26021125 | N_Shelf | -0.15 | 7.86E-07 |
| cg23259375 | chr6 | 26016732 |  | chr6:26020671-26021125 | N_Shelf | -0.12 | 0.000104259 |
| cg17261676 | chr6 | 26016808 |  | chr6:26020671-26021125 | N_Shelf | -0.22 | 1.43E-10 |
| cg07816556 | chr6 | 26017280 | *HIST1H1A* | chr6:26020671-26021125 | N_Shelf | -0.27 | 4.16E-06 |
| cg08511651 | chr6 | 26017619 | *HIST1H1A* | chr6:26020671-26021125 | N_Shelf | -0.24 | 4.61E-09 |
| cg05816193 | chr6 | 26018127 | *HIST1H1A* | chr6:26020671-26021125 | N_Shelf | -0.11 | 0.004335891 |
| cg11342453 | chr6 | 26196699 |  | chr6:26197070-26197537 | N_Shore | -0.11 | 0.000403606 |
| cg03785755 | chr6 | 26196794 |  | chr6:26197070-26197537 | N_Shore | -0.12 | 3.52E-06 |
| cg26092675 | chr6 | 26225258 | *HIST1H3E* | chr6:26225386-26225790 | N_Shore | -0.25 | 5.27E-05 |
| cg13836098 | chr6 | 26225268 | *HIST1H3E* | chr6:26225386-26225790 | N_Shore | -0.20 | 0.000193744 |
| cg27490387 | chr6 | 26520793 | *HCG11* | chr6:26521947-26522579 | N_Shore | -0.14 | 1.48E-05 |
| cg10184431 | chr6 | 27783226 | *HIST1H2AJ* | chr6:27782247-27782485 | S_Shore | -0.12 | 1.19E-06 |
| cg03221914 | chr6 | 27783331 | *HIST1H2AJ* | chr6:27782247-27782485 | S_Shore | -0.14 | 0.000879256 |
| cg14930690 | chr6 | 28973828 | *ZNF311* |  | OpenSea | -0.12 | 0.001161401 |
| cg12315353 | chr6 | 29573089 | *GABBR1* |  | OpenSea | -0.11 | 5.51E-05 |
| cg11862551 | chr6 | 29817963 |  | chr6:29818085-29818341 | N_Shore | 0.12 | 0.00740507 |
| cg22425474 | chr6 | 29818086 |  | chr6:29818085-29818341 | Island | 0.17 | 0.004075462 |
| cg10821226 | chr6 | 29818277 |  | chr6:29818085-29818341 | Island | 0.10 | 0.004133244 |
| cg27342919 | chr6 | 30079256 | *TRIM31* |  | OpenSea | 0.13 | 0.006240919 |
| cg21809927 | chr6 | 30079265 | *TRIM31* |  | OpenSea | 0.16 | 0.001158507 |
| cg08222513 | chr6 | 30079280 | *TRIM31* |  | OpenSea | 0.15 | 0.000670935 |
| cg23079252 | chr6 | 30095199 |  | chr6:30095173-30095610 | Island | 0.12 | 0.007741968 |
| cg21334513 | chr6 | 30095248 |  | chr6:30095173-30095610 | Island | 0.12 | 0.005610193 |
| cg10526841 | chr6 | 30182507 | *TRIM26* | chr6:30180933-30182187 | S_Shore | -0.11 | 1.04E-05 |
| cg12433575 | chr6 | 30881464 | *VARS2* | chr6:30881533-30882296 | N_Shore | -0.12 | 2.33E-06 |
| cg05267955 | chr6 | 30881562 | *VARS2* | chr6:30881533-30882296 | Island | -0.12 | 0.000135043 |
| cg15848685 | chr6 | 30882641 | *VARS2* | chr6:30881533-30882296 | S_Shore | -0.21 | 3.42E-05 |
| cg16958594 | chr6 | 30882708 | *VARS2* | chr6:30881533-30882296 | S_Shore | -0.17 | 0.000579775 |
| cg15978899 | chr6 | 30882994 | *VARS2* | chr6:30881533-30882296 | S_Shore | -0.13 | 4.67E-05 |
| cg00933603 | chr6 | 30883001 | *VARS2* | chr6:30881533-30882296 | S_Shore | -0.11 | 3.57E-05 |
| cg27057509 | chr6 | 30883762 | *VARS2* | chr6:30881533-30882296 | S_Shore | -0.18 | 2.02E-05 |
| cg06879746 | chr6 | 30883768 | *VARS2* | chr6:30881533-30882296 | S_Shore | -0.19 | 3.23E-05 |
| cg14324675 | chr6 | 31554848 | *LST1* |  | OpenSea | -0.10 | 0.001836784 |
| cg00811535 | chr6 | 31587543 | *BAT2* | chr6:31587779-31589024 | N_Shore | -0.12 | 0.000423181 |
| cg16500605 | chr6 | 31620861 | *BAT3* | chr6:31620740-31621158 | Island | -0.12 | 0.001550632 |
| cg22235293 | chr6 | 31691539 | *C6orf25* | chr6:31691425-31691718 | Island | -0.14 | 0.007888583 |
| cg23895220 | chr6 | 31691870 | *C6orf25* | chr6:31691425-31691718 | S_Shore | -0.11 | 0.007255001 |
| cg07739478 | chr6 | 31692234 | *C6orf25* | chr6:31691425-31691718 | S_Shore | -0.10 | 0.009935769 |
| cg20640261 | chr6 | 31707019 | *MSH5* | chr6:31707489-31708336 | N_Shore | -0.15 | 0.007593835 |
| cg20980321 | chr6 | 32144667 | *RNF5* |  | OpenSea | -0.12 | 0.000370415 |
| cg08801479 | chr6 | 32165200 | *NOTCH4* | chr6:32163292-32164383 | S_Shore | -0.17 | 0.000863886 |
| cg09715059 | chr6 | 32223336 |  |  | OpenSea | -0.13 | 2.59E-05 |
| cg00523604 | chr6 | 32223341 |  |  | OpenSea | -0.12 | 0.000197628 |
| cg15255946 | chr6 | 32729563 | *HLA-DQB2* | chr6:32729342-32729877 | Island | 0.13 | 0.005893825 |
| cg06210070 | chr6 | 33085063 | *HLA-DPB2* | chr6:33084740-33084995 | S_Shore | 0.12 | 0.001005263 |
| cg15929078 | chr6 | 33267996 | *TAPBP* | chr6:33266302-33267582 | S_Shore | -0.11 | 0.003466669 |
| cg07903144 | chr6 | 33358872 | *KIFC1* | chr6:33359097-33359975 | N_Shore | -0.13 | 0.000488943 |
| cg08371951 | chr6 | 33396610 | *SYNGAP1* | chr6:33396050-33396296 | S_Shore | -0.14 | 0.006140482 |
| cg22875823 | chr6 | 33400543 | *SYNGAP1* |  | OpenSea | -0.13 | 0.009424855 |
| cg03853124 | chr6 | 33875958 |  |  | OpenSea | -0.15 | 0.000992457 |
| cg17762073 | chr6 | 34024220 | *GRM4* | chr6:34024201-34024457 | Island | 0.13 | 0.002060266 |
| cg18735956 | chr6 | 34113908 |  | chr6:34111735-34113944 | Island | 0.12 | 0.000710269 |
| cg08188318 | chr6 | 34984953 | *ANKS1A* |  | OpenSea | -0.15 | 0.004304734 |
| cg16145324 | chr6 | 36020012 | *MAPK14* |  | OpenSea | -0.15 | 0.005087487 |
| cg23737190 | chr6 | 36930021 | *PI16* | chr6:36929837-36930135 | Island | 0.12 | 0.006348041 |
| cg24661236 | chr6 | 36930062 | *PI16* | chr6:36929837-36930135 | Island | 0.13 | 0.004049396 |
| cg08106319 | chr6 | 37849427 | *ZFAND3* |  | OpenSea | -0.14 | 0.00424147 |
| cg00049595 | chr6 | 41650636 |  | chr6:41650672-41651582 | N_Shore | -0.24 | 7.19E-10 |
| cg15823502 | chr6 | 41650768 |  | chr6:41650672-41651582 | Island | -0.17 | 1.39E-05 |
| cg04637454 | chr6 | 41651066 |  | chr6:41650672-41651582 | Island | -0.15 | 1.27E-07 |
| cg13142134 | chr6 | 42695332 |  | chr6:42694963-42695631 | Island | -0.19 | 7.56E-07 |
| cg26069291 | chr6 | 42716310 |  | chr6:42714707-42715042 | S_Shore | -0.12 | 0.0016724 |
| cg06493154 | chr6 | 42859023 | *C6orf226* | chr6:42858169-42858960 | S_Shore | -0.11 | 0.000992457 |
| cg12852800 | chr6 | 42896394 | *CNPY3* | chr6:42897116-42897611 | N_Shore | -0.12 | 3.98E-06 |
| cg16549027 | chr6 | 43149629 | *CUL9* | chr6:43149736-43150009 | N_Shore | -0.11 | 6.35E-06 |
| cg25521481 | chr6 | 43214396 | *TTBK1* | chr6:43214369-43214591 | Island | -0.11 | 0.000100531 |
| cg05394244 | chr6 | 64281439 | *PTP4A1* | chr6:64282151-64283736 | N_Shore | -0.11 | 0.002712047 |
| cg24631428 | chr6 | 64281604 | *PTP4A1* | chr6:64282151-64283736 | N_Shore | -0.10 | 0.001561068 |
| cg01281718 | chr6 | 71376634 | *SMAP1* | chr6:71377385-71378178 | N_Shore | -0.15 | 1.11E-05 |
| cg04086834 | chr6 | 71377064 | *SMAP1* | chr6:71377385-71378178 | N_Shore | -0.11 | 0.002901824 |
| cg17188169 | chr6 | 74104388 | *DDX43* | chr6:74104425-74104878 | N_Shore | -0.18 | 0.0017664 |
| cg03669949 | chr6 | 74104426 | *DDX43* | chr6:74104425-74104878 | Island | -0.21 | 0.0052223 |
| cg06797068 | chr6 | 74104600 | *DDX43* | chr6:74104425-74104878 | Island | -0.19 | 0.006058068 |
| cg16143502 | chr6 | 74104801 | *DDX43* | chr6:74104425-74104878 | Island | -0.16 | 0.004548016 |
| cg08124399 | chr6 | 74104868 | *DDX43* | chr6:74104425-74104878 | Island | -0.21 | 0.000340758 |
| cg11742202 | chr6 | 74364667 | *SLC17A5* | chr6:74363202-74364006 | S_Shore | -0.16 | 1.62E-06 |
| cg13599248 | chr6 | 74364700 | *SLC17A5* | chr6:74363202-74364006 | S_Shore | -0.16 | 1.18E-05 |
| cg06809298 | chr6 | 75953853 | *COX7A2* |  | OpenSea | -0.11 | 0.005352324 |
| cg00880984 | chr6 | 82958011 | *IBTK* | chr6:82956999-82957507 | S_Shore | -0.13 | 4.52E-05 |
| cg05316627 | chr6 | 87861261 |  | chr6:87861526-87862513 | N_Shore | -0.23 | 1.26E-05 |
| cg07937453 | chr6 | 89296807 |  |  | OpenSea | 0.10 | 0.003756222 |
| cg25362652 | chr6 | 106429769 |  | chr6:106429111-106429772 | Island | 0.20 | 1.41E-05 |
| cg15676707 | chr6 | 106434396 |  | chr6:106433984-106434459 | Island | 0.13 | 0.000487791 |
| cg09187505 | chr6 | 106434429 |  | chr6:106433984-106434459 | Island | 0.21 | 0.000877433 |
| cg14951955 | chr6 | 106773949 | *ATG5* | chr6:106773187-106773803 | S_Shore | -0.16 | 7.49E-05 |
| cg09354267 | chr6 | 106774064 | *ATG5* | chr6:106773187-106773803 | S_Shore | -0.11 | 0.000570064 |
| cg21148531 | chr6 | 106774172 | *ATG5* | chr6:106773187-106773803 | S_Shore | -0.15 | 0.001102322 |
| cg01916632 | chr6 | 107781506 | *PDSS2* | chr6:107780235-107780456 | S_Shore | -0.16 | 5.27E-06 |
| cg00847453 | chr6 | 110720501 | *DDO* | chr6:110721059-110721630 | N_Shore | -0.21 | 3.39E-07 |
| cg12078872 | chr6 | 110721629 | *DDO* | chr6:110721059-110721630 | Island | -0.14 | 3.41E-05 |
| cg04140496 | chr6 | 111279511 | *GTF3C6* | chr6:111279644-111280210 | N_Shore | -0.11 | 3.30E-05 |
| cg02359132 | chr6 | 111279585 | *GTF3C6* | chr6:111279644-111280210 | N_Shore | -0.11 | 8.42E-05 |
| cg13526264 | chr6 | 113886304 |  |  | OpenSea | 0.13 | 0.001581954 |
| cg27642470 | chr6 | 117802710 | *DCBLD1* | chr6:117803497-117804671 | N_Shore | 0.11 | 0.003854251 |
| cg23770887 | chr6 | 117995812 | *NUS1* | chr6:117996395-117997375 | N_Shore | -0.13 | 0.000211605 |
| cg18645398 | chr6 | 117995843 | *NUS1* | chr6:117996395-117997375 | N_Shore | -0.10 | 0.00063547 |
| cg21760990 | chr6 | 121656542 | *C6orf170* | chr6:121655615-121656022 | S_Shore | -0.12 | 0.000130135 |
| cg20390711 | chr6 | 127796683 | *C6orf174* | chr6:127796286-127797356 | Island | 0.15 | 0.001360933 |
| cg14079463 | chr6 | 127796989 | *C6orf174* | chr6:127796286-127797356 | Island | 0.30 | 0.000248272 |
| cg04580344 | chr6 | 127797022 | *C6orf174* | chr6:127796286-127797356 | Island | 0.25 | 0.000514791 |
| cg16983110 | chr6 | 128813319 | *PTPRK* |  | OpenSea | -0.10 | 0.000305918 |
| cg07095347 | chr6 | 133253256 |  |  | OpenSea | -0.12 | 0.009150688 |
| cg15797527 | chr6 | 135814781 | *AHI1* | chr6:135818501-135819160 | N_Shelf | 0.13 | 0.001554327 |
| cg05220968 | chr6 | 146057943 | *EPM2A* | chr6:146055896-146056981 | S_Shore | -0.18 | 3.34E-05 |
| cg03354508 | chr6 | 147769498 |  |  | OpenSea | 0.11 | 0.00126632 |
| cg20778199 | chr6 | 148020881 |  |  | OpenSea | -0.17 | 0.001690214 |
| cg07805500 | chr6 | 151380818 | *MTHFD1L* |  | OpenSea | -0.11 | 0.002315215 |
| cg19449067 | chr6 | 152011103 | *ESR1* |  | OpenSea | -0.15 | 0.003688482 |
| cg08161546 | chr6 | 152011415 | *ESR1* |  | OpenSea | -0.13 | 0.001419768 |
| cg09288789 | chr6 | 154832667 | *CNKSR3* | chr6:154830560-154832001 | S_Shore | -0.10 | 0.009233326 |
| cg07292773 | chr6 | 156718177 |  | chr6:156718071-156718547 | Island | -0.14 | 0.001638234 |
| cg21795221 | chr6 | 156718262 |  | chr6:156718071-156718547 | Island | -0.14 | 0.001296032 |
| cg09056876 | chr6 | 156718398 |  | chr6:156718071-156718547 | Island | -0.16 | 0.002221112 |
| cg20021506 | chr6 | 156718546 |  | chr6:156718071-156718547 | Island | -0.13 | 0.001876541 |
| cg15298323 | chr6 | 160182438 | *ACAT2* | chr6:160182429-160183770 | Island | -0.14 | 3.72E-06 |
| cg06131755 | chr6 | 160182447 | *ACAT2* | chr6:160182429-160183770 | Island | -0.11 | 0.000128137 |
| cg25736982 | chr6 | 160182554 | *ACAT2* | chr6:160182429-160183770 | Island | -0.12 | 5.30E-05 |
| cg12754982 | chr6 | 160409942 | *IGF2R* |  | OpenSea | -0.16 | 0.001200679 |
| cg21354781 | chr6 | 164029556 |  |  | OpenSea | 0.13 | 0.00140658 |
| cg00094255 | chr6 | 164092410 |  | chr6:164092861-164093113 | N_Shore | -0.28 | 4.16E-09 |
| cg12094863 | chr6 | 164092588 |  | chr6:164092861-164093113 | N_Shore | -0.27 | 1.99E-07 |
| cg01909738 | chr6 | 164092631 |  | chr6:164092861-164093113 | N_Shore | -0.33 | 1.51E-07 |
| cg08249780 | chr6 | 164092862 |  | chr6:164092861-164093113 | Island | -0.36 | 9.63E-08 |
| cg00244642 | chr6 | 164093014 |  | chr6:164092861-164093113 | Island | -0.38 | 4.45E-09 |
| cg22615992 | chr6 | 164093099 |  | chr6:164092861-164093113 | Island | -0.27 | 9.63E-08 |
| cg18405330 | chr6 | 164171960 |  |  | OpenSea | 0.24 | 0.006436753 |
| cg09080087 | chr6 | 168240272 | *MLLT4* |  | OpenSea | -0.10 | 0.001493339 |
| cg10781468 | chr6 | 168778312 |  |  | OpenSea | 0.11 | 0.000609488 |
| cg12758485 | chr6 | 168785396 |  |  | OpenSea | 0.12 | 0.000422973 |
| cg09462806 | chr7 | 150084 |  | chr7:149494-150038 | S_Shore | -0.11 | 1.92E-05 |
| cg08973950 | chr7 | 1083309 | *C7orf50* | chr7:1084387-1084595 | N_Shore | -0.17 | 0.000255122 |
| cg09650488 | chr7 | 1687007 |  | chr7:1686716-1687008 | Island | 0.12 | 0.00134127 |
| cg13581527 | chr7 | 1708566 |  | chr7:1703861-1710517 | Island | 0.14 | 1.08E-05 |
| cg24301866 | chr7 | 1708659 |  | chr7:1703861-1710517 | Island | 0.10 | 0.001133568 |
| cg15050051 | chr7 | 1782000 | *ELFN1* | chr7:1784027-1786934 | N_Shelf | -0.12 | 0.006196177 |
| cg22703520 | chr7 | 3762272 | *SDK1* |  | OpenSea | 0.15 | 1.67E-05 |
| cg16321846 | chr7 | 4050114 | *SDK1* |  | OpenSea | -0.15 | 0.000211968 |
| cg23474811 | chr7 | 4183807 | *SDK1* |  | OpenSea | 0.10 | 0.005298308 |
| cg04612959 | chr7 | 4183976 | *SDK1* |  | OpenSea | 0.13 | 0.003424167 |
| cg25879142 | chr7 | 4671391 |  |  | OpenSea | 0.12 | 0.009020707 |
| cg15246238 | chr7 | 5635134 | *FSCN1* | chr7:5632335-5634555 | S_Shore | -0.10 | 0.009966041 |
| cg20117519 | chr7 | 8429907 |  |  | OpenSea | 0.12 | 0.003756222 |
| cg27578754 | chr7 | 11012967 | *PHF14* | chr7:11013426-11013888 | N_Shore | -0.12 | 0.008141557 |
| cg09259772 | chr7 | 12726089 | *ARL4A* | chr7:12726156-12727248 | N_Shore | -0.16 | 3.13E-08 |
| cg20400838 | chr7 | 21208923 |  |  | OpenSea | 0.14 | 0.000145673 |
| cg27385193 | chr7 | 21208954 |  |  | OpenSea | 0.13 | 0.000317438 |
| cg24803391 | chr7 | 21209338 |  |  | OpenSea | 0.10 | 0.000402315 |
| cg16448636 | chr7 | 21209509 |  |  | OpenSea | 0.11 | 0.005439159 |
| cg09755872 | chr7 | 23245557 |  | chr7:23245556-23246161 | Island | -0.19 | 3.15E-08 |
| cg16180796 | chr7 | 23245900 |  | chr7:23245556-23246161 | Island | -0.19 | 0.000842528 |
| cg18081818 | chr7 | 23246105 |  | chr7:23245556-23246161 | Island | -0.36 | 9.57E-09 |
| cg23302989 | chr7 | 25174492 | *C7orf31* |  | OpenSea | -0.16 | 0.007985166 |
| cg02248486 | chr7 | 27183196 | *HOXA5* | chr7:27182613-27185562 | Island | 0.15 | 0.009849945 |
| cg13817046 | chr7 | 29609310 |  | chr7:29605827-29606290 | S_Shelf | -0.14 | 0.007471819 |
| cg06257936 | chr7 | 31082763 |  |  | OpenSea | 0.10 | 0.007997691 |
| cg17588800 | chr7 | 42278067 | *GLI3* | chr7:42276003-42277850 | S_Shore | 0.10 | 0.000365472 |
| cg24497732 | chr7 | 42278077 | *GLI3* | chr7:42276003-42277850 | S_Shore | 0.12 | 1.67E-06 |
| cg26557756 | chr7 | 42278096 | *GLI3* | chr7:42276003-42277850 | S_Shore | 0.13 | 0.005200988 |
| cg27549208 | chr7 | 43965451 | *URGCP* | chr7:43965806-43966400 | N_Shore | -0.21 | 0.004030794 |
| cg05072008 | chr7 | 50518647 | *FIGNL1* | chr7:50517627-50518668 | Island | -0.21 | 3.97E-08 |
| cg08311085 | chr7 | 53879422 |  |  | OpenSea | -0.13 | 0.001919606 |
| cg11552287 | chr7 | 55432692 | *LANCL2* | chr7:55433147-55434271 | N_Shore | -0.11 | 0.007828639 |
| cg01587386 | chr7 | 65970924 |  | chr7:65970022-65971421 | Island | -0.14 | 8.67E-05 |
| cg25985355 | chr7 | 65971099 |  | chr7:65970022-65971421 | Island | -0.16 | 0.005459554 |
| cg00277397 | chr7 | 71800412 | *CALN1* | chr7:71800757-71802768 | N_Shore | -0.12 | 0.003886097 |
| cg22558871 | chr7 | 72723150 | *NSUN5* | chr7:72722327-72722967 | S_Shore | -0.15 | 0.007913626 |
| cg27441048 | chr7 | 72972316 | *BCL7B* | chr7:72971527-72972220 | S_Shore | -0.15 | 5.27E-07 |
| cg00506049 | chr7 | 75887380 | *SRRM3* | chr7:75889086-75889345 | N_Shore | 0.11 | 0.001562462 |
| cg01499518 | chr7 | 75890022 | *SRRM3* | chr7:75889086-75889345 | S_Shore | 0.14 | 0.006932247 |
| cg01614764 | chr7 | 76023325 | *SRCRB4D* | chr7:76022369-76023172 | S_Shore | -0.16 | 2.31E-05 |
| cg02407048 | chr7 | 77046134 | *PION* | chr7:77044840-77045719 | S_Shore | -0.14 | 0.006036321 |
| cg04085585 | chr7 | 77648378 | *MAGI2* | chr7:77648451-77648668 | N_Shore | -0.10 | 0.000833884 |
| cg18676790 | chr7 | 97680731 |  |  | OpenSea | 0.11 | 0.000821207 |
| cg06915202 | chr7 | 98029285 | *BAIAP2L1* | chr7:98030097-98030422 | N_Shore | 0.15 | 0.001395968 |
| cg02888048 | chr7 | 98335163 |  |  | OpenSea | 0.11 | 0.006156009 |
| cg12290671 | chr7 | 99195819 |  | chr7:99195683-99196001 | Island | 0.12 | 0.007224433 |
| cg02756107 | chr7 | 99517460 | *TRIM4* | chr7:99516602-99517296 | S_Shore | -0.11 | 5.98E-05 |
| cg25591451 | chr7 | 99517509 | *TRIM4* | chr7:99516602-99517296 | S_Shore | -0.13 | 0.000100203 |
| cg17607973 | chr7 | 100027408 | *MEPCE* | chr7:100025804-100028002 | Island | 0.11 | 0.003157405 |
| cg12616177 | chr7 | 100434510 |  | chr7:100434982-100435193 | N_Shore | -0.13 | 0.000155575 |
| cg22851200 | chr7 | 100465833 | *TRIP6* | chr7:100463758-100464146 | S_Shore | -0.11 | 0.005167586 |
| cg19566658 | chr7 | 100466241 | *TRIP6* | chr7:100463758-100464146 | S_Shelf | -0.13 | 0.000952301 |
| cg18683606 | chr7 | 100471612 | *SRRT* | chr7:100472375-100473393 | N_Shore | -0.12 | 7.80E-05 |
| cg08558340 | chr7 | 100472263 | *SRRT* | chr7:100472375-100473393 | N_Shore | -0.26 | 1.26E-09 |
| cg10426581 | chr7 | 100472382 | *SRRT* | chr7:100472375-100473393 | Island | -0.17 | 3.31E-06 |
| cg17944572 | chr7 | 100888748 | *FIS1* | chr7:100888069-100888488 | S_Shore | -0.11 | 8.42E-05 |
| cg08159594 | chr7 | 102790168 | *NAPEPLD* | chr7:102789395-102790120 | S_Shore | -0.15 | 1.49E-07 |
| cg27282281 | chr7 | 112262583 |  |  | OpenSea | -0.12 | 0.001030776 |
| cg23628563 | chr7 | 112262597 |  |  | OpenSea | -0.11 | 0.000559087 |
| cg25509184 | chr7 | 117119382 | *CFTR* |  | OpenSea | 0.10 | 0.006285923 |
| cg26635219 | chr7 | 117119424 | *CFTR* |  | OpenSea | 0.12 | 0.001073096 |
| cg00480856 | chr7 | 123390035 | *WASL* | chr7:123388399-123389623 | S_Shore | -0.15 | 0.009237831 |
| cg00448395 | chr7 | 124570359 | *POT1* | chr7:124569882-124570199 | S_Shore | -0.21 | 0.001630049 |
| cg02945056 | chr7 | 127225891 | *GCC1* | chr7:127225200-127225950 | Island | -0.10 | 0.001530616 |
| cg23081781 | chr7 | 127225937 | *GCC1* | chr7:127225200-127225950 | Island | -0.14 | 7.26E-05 |
| cg08586737 | chr7 | 127225949 | *GCC1* | chr7:127225200-127225950 | Island | -0.15 | 0.000992457 |
| cg08234689 | chr7 | 127910927 |  | chr7:127910860-127911287 | Island | -0.32 | 4.10E-06 |
| cg21885361 | chr7 | 127911034 |  | chr7:127910860-127911287 | Island | -0.24 | 9.82E-06 |
| cg26209990 | chr7 | 127911258 |  | chr7:127910860-127911287 | Island | -0.28 | 6.22E-05 |
| cg20245362 | chr7 | 127911367 |  | chr7:127911719-127912194 | N_Shore | -0.24 | 0.000160245 |
| cg07617283 | chr7 | 127913660 |  | chr7:127911719-127912194 | S_Shore | -0.14 | 0.005712929 |
| cg24176311 | chr7 | 128696150 | *LOC286016* | chr7:128696026-128696230 | Island | -0.20 | 0.001630049 |
| cg04637704 | chr7 | 128696180 | *LOC286016* | chr7:128696026-128696230 | Island | -0.18 | 0.001022535 |
| cg13986840 | chr7 | 130132453 | *MEST* | chr7:130130739-130133111 | Island | 0.14 | 0.001161401 |
| cg14952237 | chr7 | 130132790 | *MEST* | chr7:130130739-130133111 | Island | 0.11 | 0.000645137 |
| cg27535211 | chr7 | 132160221 | *PLXNA4* |  | OpenSea | 0.11 | 3.29E-05 |
| cg25009327 | chr7 | 148820504 | *ZNF425* | chr7:148823194-148824228 | N_Shelf | 0.16 | 0.002146212 |
| cg22376262 | chr7 | 148959887 | *ZNF783* | chr7:148958858-148959891 | Island | -0.20 | 8.60E-11 |
| cg04388919 | chr7 | 149119133 |  | chr7:149119398-149120307 | N_Shore | -0.14 | 0.000914497 |
| cg08634133 | chr7 | 149570071 | *ATP6V0E2* | chr7:149570366-149571272 | N_Shore | -0.10 | 0.003424462 |
| cg24091438 | chr7 | 150019955 | *LRRC61* | chr7:150019950-150020752 | Island | -0.21 | 1.08E-10 |
| cg06975979 | chr7 | 150020025 | *LRRC61* | chr7:150019950-150020752 | Island | -0.21 | 2.58E-09 |
| cg19183166 | chr7 | 150020108 | *LRRC61* | chr7:150019950-150020752 | Island | -0.15 | 8.81E-07 |
| cg03185704 | chr7 | 150020125 | *LRRC61* | chr7:150019950-150020752 | Island | -0.16 | 1.17E-07 |
| cg18221429 | chr7 | 150020136 | *LRRC61* | chr7:150019950-150020752 | Island | -0.11 | 3.52E-06 |
| cg07914621 | chr7 | 150101498 | *LOC728743* | chr7:150101906-150102968 | N_Shore | -0.11 | 1.46E-05 |
| cg04839131 | chr7 | 150644715 | *KCNH2* | chr7:150644455-150644767 | Island | 0.12 | 0.00353013 |
| cg20498962 | chr7 | 150705760 | *NOS3* | chr7:150705759-150706129 | Island | 0.10 | 0.009662211 |
| cg01572810 | chr7 | 150901654 |  | chr7:150901550-150901978 | Island | 0.17 | 0.000135448 |
| cg04795774 | chr7 | 150901733 |  | chr7:150901550-150901978 | Island | 0.15 | 0.000413956 |
| cg21134096 | chr7 | 151217883 | *RHEB* | chr7:151216068-151217901 | Island | -0.10 | 0.000218147 |
| cg24531520 | chr7 | 151217947 | *RHEB* | chr7:151216068-151217901 | S_Shore | -0.10 | 9.57E-05 |
| cg06603923 | chr7 | 151217961 | *RHEB* | chr7:151216068-151217901 | S_Shore | -0.18 | 9.56E-07 |
| cg13810766 | chr7 | 151542452 | *PRKAG2* |  | OpenSea | 0.10 | 0.001626982 |
| cg00802903 | chr7 | 155592263 |  | chr7:155595692-155599414 | N_Shelf | 0.13 | 0.004989817 |
| cg18628367 | chr7 | 157475548 | *PTPRN2* | chr7:157476886-157486719 | N_Shore | 0.10 | 0.007390848 |
| cg18285788 | chr7 | 157475692 | *PTPRN2* | chr7:157476886-157486719 | N_Shore | 0.11 | 0.000512684 |
| cg03983213 | chr7 | 157475737 | *PTPRN2* | chr7:157476886-157486719 | N_Shore | 0.12 | 5.20E-05 |
| cg11072645 | chr7 | 157754746 | *PTPRN2* |  | OpenSea | 0.12 | 0.00466474 |
| cg23455837 | chr7 | 157809325 | *PTPRN2* |  | OpenSea | 0.11 | 0.000748005 |
| cg04134279 | chr8 | 496327 | *C8orf42* | chr8:494155-496083 | S_Shore | 0.17 | 0.00646685 |
| cg25722644 | chr8 | 6694415 | *XKR5* | chr8:6691833-6693135 | S_Shore | -0.20 | 0.003201432 |
| cg21006539 | chr8 | 6793506 | *DEFA4* |  | OpenSea | -0.10 | 0.004699132 |
| cg19657214 | chr8 | 6794872 | *DEFA4* |  | OpenSea | -0.14 | 0.00154568 |
| cg23703303 | chr8 | 7720259 | *SPAG11A* |  | OpenSea | -0.13 | 0.007427594 |
| cg20165746 | chr8 | 8870185 | *ERI1* |  | OpenSea | -0.14 | 0.007596365 |
| cg12996100 | chr8 | 11470690 |  | chr8:11471014-11471249 | N_Shore | 0.10 | 0.00546612 |
| cg01003803 | chr8 | 16859632 | *FGF20* | chr8:16859044-16859452 | S_Shore | 0.11 | 0.000594345 |
| cg03080147 | chr8 | 17270347 | *MTMR7* | chr8:17270603-17271120 | N_Shore | 0.11 | 0.003681268 |
| cg05056638 | chr8 | 24800824 |  | chr8:24799703-24800147 | S_Shore | 0.23 | 0.006845752 |
| cg25352397 | chr8 | 24800883 |  | chr8:24799703-24800147 | S_Shore | 0.16 | 0.000263852 |
| cg11688949 | chr8 | 27115956 | *STMN4* |  | OpenSea | 0.14 | 0.002932749 |
| cg21210376 | chr8 | 27695667 | *PBK* | chr8:27695095-27695479 | S_Shore | -0.10 | 0.000129352 |
| cg06816054 | chr8 | 27695695 | *PBK* | chr8:27695095-27695479 | S_Shore | -0.10 | 0.000904388 |
| cg21887430 | chr8 | 27695858 | *PBK* | chr8:27695095-27695479 | S_Shore | -0.11 | 0.000789868 |
| cg17973773 | chr8 | 27695870 | *PBK* | chr8:27695095-27695479 | S_Shore | -0.12 | 3.67E-05 |
| cg09322573 | chr8 | 28961356 | *KIF13B* |  | OpenSea | -0.14 | 0.005379637 |
| cg15139038 | chr8 | 29019587 | *KIF13B* |  | OpenSea | -0.12 | 0.002745929 |
| cg20893180 | chr8 | 36957379 |  |  | OpenSea | 0.13 | 3.37E-05 |
| cg11052259 | chr8 | 38964784 | *ADAM32* | chr8:38965013-38965423 | N_Shore | -0.16 | 0.004885513 |
| cg03780701 | chr8 | 38965386 | *ADAM32* | chr8:38965013-38965423 | Island | -0.17 | 0.009344698 |
| cg16540080 | chr8 | 38965492 | *ADAM32* | chr8:38965013-38965423 | S_Shore | -0.11 | 0.001888565 |
| cg20468415 | chr8 | 41592973 | *ANK1* |  | OpenSea | -0.14 | 0.002627689 |
| cg23536158 | chr8 | 48172746 | *KIAA0146* | chr8:48172791-48172993 | N_Shore | -0.12 | 0.001790353 |
| cg00079785 | chr8 | 48172764 | *KIAA0146* | chr8:48172791-48172993 | N_Shore | -0.19 | 2.39E-07 |
| cg12846837 | chr8 | 48172889 | *KIAA0146* | chr8:48172791-48172993 | Island | -0.31 | 2.16E-10 |
| cg04220541 | chr8 | 48172992 | *KIAA0146* | chr8:48172791-48172993 | Island | -0.27 | 2.51E-08 |
| cg18757405 | chr8 | 49183060 |  |  | OpenSea | 0.11 | 0.000374226 |
| cg18932699 | chr8 | 64518293 |  |  | OpenSea | 0.13 | 0.003441782 |
| cg00326908 | chr8 | 66755120 | *PDE7A* | chr8:66753589-66754950 | S_Shore | -0.12 | 0.000127679 |
| cg27618939 | chr8 | 71581788 | *XKR9* | chr8:71581050-71581650 | S_Shore | -0.11 | 0.000398674 |
| cg17662034 | chr8 | 74207518 | *RDH10* | chr8:74206223-74208179 | Island | 0.13 | 0.001997245 |
| cg26754262 | chr8 | 75617860 | *MIR2052* |  | OpenSea | -0.14 | 0.003658724 |
| cg00849990 | chr8 | 86090773 | *E2F5* | chr8:86089201-86090287 | S_Shore | -0.11 | 0.001550691 |
| cg16814680 | chr8 | 91681699 |  |  | OpenSea | 0.28 | 0.007124858 |
| cg18507018 | chr8 | 92035517 | *TMEM55A* |  | OpenSea | -0.23 | 0.005149252 |
| cg17118836 | chr8 | 94508434 |  |  | OpenSea | -0.12 | 2.82E-06 |
| cg06699564 | chr8 | 95651099 |  | chr8:95651206-95651760 | N_Shore | 0.12 | 0.00167278 |
| cg09730795 | chr8 | 97174382 | *GDF6* | chr8:97172634-97173880 | S_Shore | 0.14 | 0.00047347 |
| cg14651183 | chr8 | 97248129 | *UQCRB* |  | OpenSea | -0.11 | 0.000202841 |
| cg01892689 | chr8 | 97657072 | *PGCP* | chr8:97657174-97657943 | N_Shore | -0.10 | 0.002430679 |
| cg22328208 | chr8 | 98289745 | *TSPYL5* | chr8:98289604-98290404 | Island | -0.36 | 1.92E-07 |
| cg15747595 | chr8 | 98289880 | *TSPYL5* | chr8:98289604-98290404 | Island | -0.13 | 0.000244698 |
| cg00186701 | chr8 | 98290510 | *TSPYL5* | chr8:98289604-98290404 | S_Shore | -0.16 | 1.27E-08 |
| cg10409981 | chr8 | 99984763 |  | chr8:99984584-99985072 | Island | 0.17 | 0.000411943 |
| cg20909645 | chr8 | 99985049 |  | chr8:99984584-99985072 | Island | 0.12 | 0.001133568 |
| cg07757887 | chr8 | 104382523 | *CTHRC1* | chr8:104383409-104384109 | N_Shore | -0.11 | 0.003137274 |
| cg19188612 | chr8 | 104384291 | *CTHRC1* | chr8:104383409-104384109 | S_Shore | -0.10 | 0.007760558 |
| cg09305680 | chr8 | 117778069 | *UTP23* | chr8:117778487-117779146 | N_Shore | -0.14 | 0.008179875 |
| cg02161761 | chr8 | 120868748 | *DSCC1* | chr8:120867785-120868440 | S_Shore | -0.13 | 0.000111866 |
| cg07745674 | chr8 | 124171207 |  | chr8:124172800-124173541 | N_Shore | 0.10 | 5.23E-05 |
| cg10105237 | chr8 | 135726091 | *ZFAT* | chr8:135724770-135725552 | S_Shore | -0.16 | 1.75E-06 |
| cg00057272 | chr8 | 135726252 | *ZFAT* | chr8:135724770-135725552 | S_Shore | -0.15 | 0.000139821 |
| cg07437919 | chr8 | 142234483 | *SLC45A4* | chr8:142236388-142236589 | N_Shore | 0.13 | 0.004099676 |
| cg12873476 | chr8 | 142402728 |  | chr8:142401533-142402494 | S_Shore | -0.17 | 2.21E-06 |
| cg24420534 | chr8 | 143177785 |  |  | OpenSea | 0.12 | 0.00474045 |
| cg15154047 | chr8 | 143210309 |  |  | OpenSea | 0.10 | 0.000119076 |
| cg06070970 | chr8 | 144359074 | *GLI4* | chr8:144357997-144359075 | Island | 0.17 | 0.004428463 |
| cg24107665 | chr8 | 144372932 | *ZNF696* | chr8:144372931-144373839 | Island | -0.10 | 0.000992457 |
| cg05913474 | chr8 | 144815204 | *FAM83H* | chr8:144815203-144816518 | Island | -0.16 | 4.00E-07 |
| cg26039042 | chr8 | 144815275 | *FAM83H* | chr8:144815203-144816518 | Island | -0.13 | 5.31E-07 |
| cg01285435 | chr8 | 145654565 | *VPS28* | chr8:145654517-145654810 | Island | -0.17 | 0.000207065 |
| cg26440467 | chr8 | 145654605 | *VPS28* | chr8:145654517-145654810 | Island | -0.16 | 2.31E-06 |
| cg05807444 | chr8 | 145654635 | *VPS28* | chr8:145654517-145654810 | Island | -0.15 | 0.000138601 |
| cg11882377 | chr8 | 145654780 | *VPS28* | chr8:145654517-145654810 | Island | -0.15 | 0.000111885 |
| cg16499645 | chr8 | 145654854 | *VPS28* | chr8:145653335-145654145 | S_Shore | -0.12 | 0.005281262 |
| cg20394284 | chr9 | 4984206 | *JAK2* | chr9:4984543-4985630 | N_Shore | -0.14 | 0.000411943 |
| cg13762612 | chr9 | 6703605 |  | chr9:6703948-6704662 | N_Shore | -0.13 | 0.003751248 |
| cg14577706 | chr9 | 13280025 |  | chr9:13278312-13279805 | S_Shore | -0.14 | 0.007656709 |
| cg05779272 | chr9 | 33168018 | *B4GALT1* | chr9:33166700-33167934 | S_Shore | -0.11 | 5.87E-06 |
| cg14408997 | chr9 | 34050096 | *UBAP2* | chr9:34048639-34049615 | S_Shore | -0.12 | 1.19E-06 |
| cg27416261 | chr9 | 35406590 | *LOC158381* |  | OpenSea | 0.11 | 0.005545507 |
| cg21179305 | chr9 | 37119968 | *ZCCHC7* | chr9:37120050-37120985 | N_Shore | -0.12 | 5.99E-05 |
| cg11304734 | chr9 | 37485592 | *POLR1E* | chr9:37485801-37486099 | N_Shore | -0.10 | 2.69E-07 |
| cg16832407 | chr9 | 73736539 | *TRPM3* |  | OpenSea | 0.12 | 0.000162284 |
| cg21249093 | chr9 | 73737300 | *TRPM3* |  | OpenSea | 0.10 | 0.006757852 |
| cg14134003 | chr9 | 88952877 | *ZCCHC6* |  | OpenSea | -0.14 | 0.006294721 |
| cg13705830 | chr9 | 89518557 |  |  | OpenSea | 0.12 | 0.003761423 |
| cg14159704 | chr9 | 89580839 |  |  | OpenSea | -0.10 | 0.005094673 |
| cg21171204 | chr9 | 89766723 | *C9orf170* | chr9:89763336-89763767 | S_Shelf | -0.12 | 1.54E-05 |
| cg14307853 | chr9 | 91924749 | *CKS2* | chr9:91925372-91926639 | N_Shore | -0.18 | 2.21E-06 |
| cg13754720 | chr9 | 98274853 | *PTCH1* | chr9:98272643-98273383 | S_Shore | 0.11 | 0.008277234 |
| cg14323293 | chr9 | 98280076 | *PTCH1* | chr9:98278128-98279754 | S_Shore | 0.15 | 0.006704676 |
| cg21198712 | chr9 | 98534327 |  |  | OpenSea | 0.12 | 0.004164849 |
| cg14563637 | chr9 | 98931801 |  |  | OpenSea | 0.11 | 0.000272808 |
| cg01825213 | chr9 | 98979965 |  | chr9:98980493-98980759 | N_Shore | 0.11 | 0.000101215 |
| cg14625636 | chr9 | 100000026 | *KIAA1529* | chr9:100000463-100000820 | N_Shore | -0.39 | 5.13E-07 |
| cg13713218 | chr9 | 100000033 | *KIAA1529* | chr9:100000463-100000820 | N_Shore | -0.38 | 0.00022504 |
| cg06643227 | chr9 | 114557239 |  | chr9:114557287-114557576 | N_Shore | -0.14 | 0.001200214 |
| cg13688329 | chr9 | 114557390 |  | chr9:114557287-114557576 | Island | -0.15 | 0.00797492 |
| cg13314167 | chr9 | 114557553 |  | chr9:114557287-114557576 | Island | -0.18 | 0.00248128 |
| cg09619598 | chr9 | 115512530 | *SNX30* | chr9:115512611-115513757 | N_Shore | -0.11 | 0.000111885 |
| cg13967908 | chr9 | 117159457 |  | chr9:117160139-117161064 | N_Shore | -0.11 | 0.004308646 |
| cg18521771 | chr9 | 117373239 | *C9orf91* | chr9:117373484-117374076 | N_Shore | -0.11 | 3.41E-05 |
| cg13501527 | chr9 | 123640474 | *PHF19* | chr9:123638820-123639936 | S_Shore | -0.14 | 3.35E-06 |
| cg14365564 | chr9 | 124133079 | *STOM* | chr9:124132057-124133095 | Island | -0.16 | 9.64E-06 |
| cg18676053 | chr9 | 124133094 | *STOM* | chr9:124132057-124133095 | Island | -0.17 | 4.79E-05 |
| cg13628022 | chr9 | 124726047 | *TTLL11* |  | OpenSea | -0.10 | 0.000148836 |
| cg17930194 | chr9 | 124982834 | *LHX6* | chr9:124981535-124982835 | Island | -0.11 | 0.001230512 |
| cg13655082 | chr9 | 125109046 |  | chr9:125109007-125109644 | Island | -0.19 | 2.62E-06 |
| cg14335349 | chr9 | 127148781 | *PSMB7* |  | OpenSea | -0.20 | 7.35E-06 |
| cg07957491 | chr9 | 134407190 | *UCK1* | chr9:134406025-134407173 | S_Shore | -0.13 | 0.000115918 |
| cg14560040 | chr9 | 136203305 | *SURF6* | chr9:136202804-136203247 | S_Shore | -0.11 | 5.72E-07 |
| cg13465995 | chr9 | 136203387 | *SURF6* | chr9:136202804-136203247 | S_Shore | -0.17 | 2.26E-10 |
| cg06638811 | chr9 | 137030555 | *RNU6ATAC* | chr9:137028717-137030556 | Island | -0.18 | 2.63E-10 |
| cg03997643 | chr9 | 137030726 | *RNU6ATAC* | chr9:137028717-137030556 | S_Shore | -0.11 | 2.34E-05 |
| cg00573124 | chr9 | 139011777 | *C9orf69* | chr9:139009862-139011272 | S_Shore | -0.11 | 3.26E-05 |
| cg14276557 | chr9 | 139538535 |  |  | OpenSea | -0.11 | 0.004673229 |
| cg01594949 | chr9 | 140197890 | *NRARP* | chr9:140195501-140197475 | S_Shore | -0.13 | 0.001860245 |
| cg14495033 | chr9 | 140707378 | *EHMT1* | chr9:140708852-140709066 | N_Shore | 0.14 | 0.002432176 |

Table S2- The most differentiating and non-redundant probes selected from the epi-signature of Claes-Jensen syndrome (n=198)

| **Probe** | **Chr** | **Position** | **Gene** | **Methylation Difference** | **Area Under the Curve** |
| --- | --- | --- | --- | --- | --- |
| cg15090440 | chr1 | 3099233 | *PRDM16* | -0.19 | 1 |
| cg09159285 | chr1 | 6664134 | *KLHL21* | -0.18 | 1 |
| cg03978675 | chr1 | 16679500 | *FBXO42* | -0.12 | 1 |
| cg27298252 | chr1 | 24645380 | *GRHL3* | -0.16 | 1 |
| cg25841309 | chr1 | 27248983 | *NUDC* | -0.16 | 1 |
| cg06346081 | chr1 | 42921584 | *PPCS* | -0.12 | 1 |
| cg21219903 | chr1 | 43920090 | *HYI* | -0.17 | 1 |
| cg13951491 | chr1 | 45793032 | *HPDL* | -0.22 | 1 |
| cg16593917 | chr1 | 45793300 | *HPDL* | -0.11 | 1 |
| cg24737783 | chr1 | 109204304 | *C1orf59* | -0.11 | 1 |
| cg05950212 | chr1 | 149860711 | *HIST2H2AB* | -0.17 | 1 |
| cg24364827 | chr1 | 149871658 | *BOLA1* | -0.12 | 1 |
| cg10589385 | chr1 | 150898437 | *SETDB1* | -0.21 | 1 |
| cg04065086 | chr1 | 151104186 | *SEMA6C* | -0.13 | 1 |
| cg26189283 | chr1 | 155109378 | *RAG1AP1* | -0.15 | 1 |
| cg23915527 | chr1 | 161368787 |  | -0.24 | 1 |
| cg24338780 | chr1 | 174968123 | *CACYBP* | -0.11 | 1 |
| cg00567190 | chr1 | 211556508 | *C1orf97* | -0.12 | 1 |
| cg24455383 | chr1 | 243736307 | *AKT3* | 0.18 | 1 |
| cg00533390 | chr1 | 245028657 | *HNRNPU* | -0.18 | 1 |
| cg17379932 | chr10 | 12085598 | *UPF2* | -0.23 | 1 |
| cg12845268 | chr10 | 63657363 |  | -0.19 | 1 |
| cg04973995 | chr10 | 74057977 |  | -0.11 | 1 |
| cg14351425 | chr10 | 120968739 | *GRK5* | 0.16 | 1 |
| cg09859240 | chr10 | 121633447 | *C10orf119* | -0.17 | 1 |
| cg03804621 | chr10 | 124638756 | *FAM24B* | -0.20 | 1 |
| cg26201213 | chr10 | 131265796 | *MGMT* | -0.11 | 1 |
| cg21565415 | chr11 | 618993 | *MUPCDH* | 0.16 | 1 |
| cg15352671 | chr11 | 1331497 | *LOC255512* | -0.21 | 1 |
| cg22424444 | chr11 | 1331736 | *LOC255512* | -0.25 | 1 |
| cg21245372 | chr11 | 3819539 | *PGAP2* | -0.11 | 1 |
| cg20208600 | chr11 | 61159687 | *TMEM216* | -0.16 | 1 |
| cg24147428 | chr11 | 65409760 | *SIPA1* | -0.17 | 1 |
| cg06456738 | chr11 | 66361081 | *CCDC87* | -0.11 | 1 |
| cg18725375 | chr11 | 86142478 |  | -0.24 | 1 |
| cg21127537 | chr11 | 113184936 | *TTC12* | -0.14 | 1 |
| cg24758816 | chr11 | 125756762 | *HYLS1* | -0.11 | 1 |
| cg17631150 | chr11 | 126173261 | *DCPS* | -0.14 | 1 |
| cg14482569 | chr11 | 130185651 | *ZBTB44* | -0.17 | 1 |
| cg18816701 | chr12 | 2800055 | *CACNA1C* | -0.12 | 1 |
| cg16257219 | chr12 | 3069765 | *TEAD4* | 0.14 | 1 |
| cg19360852 | chr12 | 12848977 | *GPR19* | -0.13 | 1 |
| cg18048953 | chr12 | 12867753 |  | -0.14 | 1 |
| cg23327859 | chr12 | 49111433 | *CCNT1* | -0.12 | 1 |
| cg10695105 | chr12 | 49943166 | *KCNH3* | -0.12 | 1 |
| cg24210717 | chr12 | 50497827 | *GPD1* | 0.13 | 1 |
| cg02308712 | chr12 | 52598204 |  | 0.13 | 1 |
| cg22813430 | chr12 | 52626427 | *KRT7* | 0.15 | 1 |
| cg02491754 | chr12 | 53773040 | *SP1* | -0.18 | 1 |
| cg06868955 | chr12 | 54069197 | *ATP5G2* | -0.19 | 1 |
| cg22997177 | chr12 | 54070527 | *ATP5G2* | -0.13 | 1 |
| cg04831505 | chr12 | 72233240 | *TBC1D15* | -0.11 | 1 |
| cg03223580 | chr12 | 76742630 | *BBS10* | -0.10 | 1 |
| cg11207300 | chr12 | 95867190 | *METAP2* | -0.14 | 1 |
| cg10378667 | chr12 | 122326303 | *PSMD9* | -0.10 | 1 |
| cg20670946 | chr12 | 122502170 |  | -0.16 | 1 |
| cg26853536 | chr12 | 125399964 | *UBC* | -0.13 | 1 |
| cg01841471 | chr13 | 20879896 |  | 0.11 | 1 |
| cg20147645 | chr13 | 28023563 | *MTIF3* | -0.14 | 1 |
| cg01757116 | chr13 | 34118017 |  | -0.18 | 1 |
| cg01404873 | chr13 | 50701050 | *DLEU2* | -0.28 | 1 |
| cg08274637 | chr13 | 51417923 | *DLEU7* | -0.22 | 1 |
| cg04015777 | chr13 | 103426481 | *C13orf27* | -0.15 | 1 |
| cg00917437 | chr14 | 55879046 | *KIAA0831* | -0.14 | 1 |
| cg04752257 | chr14 | 58862417 | *TOMM20L* | -0.12 | 1 |
| cg23373640 | chr14 | 65696480 |  | -0.21 | 1 |
| cg19194924 | chr14 | 90168307 |  | -0.10 | 1 |
| cg06607384 | chr14 | 91579859 | *C14orf159* | -0.13 | 1 |
| cg01121022 | chr14 | 104338788 |  | -0.11 | 1 |
| cg05881762 | chr15 | 25684849 | *UBE3A* | -0.14 | 1 |
| cg17082938 | chr15 | 44828179 | *EIF3J* | -0.14 | 1 |
| cg04036182 | chr15 | 45458818 |  | -0.14 | 1 |
| cg00202460 | chr15 | 83680873 | *C15orf40* | -0.13 | 1 |
| cg10646368 | chr15 | 83974335 |  | 0.12 | 1 |
| cg08961287 | chr15 | 89919993 | *LOC254559* | 0.14 | 1 |
| cg09169617 | chr15 | 89921672 | *LOC254559* | 0.14 | 1 |
| cg08296037 | chr16 | 1584118 | *IFT140* | -0.23 | 1 |
| cg09549813 | chr16 | 4587862 | *C16orf5* | -0.15 | 1 |
| cg00589006 | chr16 | 70415864 | *ST3GAL2* | -0.11 | 1 |
| cg01329973 | chr16 | 86912065 |  | 0.10 | 1 |
| cg05324407 | chr16 | 86987828 |  | 0.18 | 1 |
| cg12065943 | chr17 | 19881925 | *AKAP10* | -0.17 | 1 |
| cg22584138 | chr17 | 28562220 | *SLC6A4* | 0.14 | 1 |
| cg19602315 | chr17 | 30669494 | *C17orf75* | -0.14 | 1 |
| cg07438660 | chr17 | 38805042 | *SMARCE1* | -0.11 | 1 |
| cg16347155 | chr17 | 40687830 | *NAGLU* | -0.12 | 1 |
| cg07967210 | chr17 | 47022446 | *SNF8* | -0.12 | 1 |
| cg10824354 | chr17 | 47049956 |  | 0.10 | 1 |
| cg20640281 | chr17 | 53341592 | *HLF* | -0.12 | 1 |
| cg21122199 | chr17 | 58499720 | *C17orf64* | -0.16 | 1 |
| cg25814096 | chr17 | 73782472 | *UNK* | -0.10 | 1 |
| cg01453052 | chr17 | 73892097 | *TRIM65* | -0.14 | 1 |
| cg05331731 | chr18 | 11147146 | *FAM38B* | 0.14 | 1 |
| cg06033764 | chr18 | 29671448 | *RNF138* | -0.11 | 1 |
| cg06487082 | chr18 | 56530106 | *ZNF532* | -0.14 | 1 |
| cg06556497 | chr18 | 76828521 | *ATP9B* | -0.28 | 1 |
| cg01875838 | chr19 | 10947446 | *TMED1* | -0.17 | 1 |
| cg21517055 | chr19 | 14016717 | *C19orf57* | -0.12 | 1 |
| cg03233793 | chr19 | 19626605 | *NDUFA13* | -0.13 | 1 |
| cg21498471 | chr19 | 33183713 | *NUDT19* | -0.16 | 1 |
| cg04731926 | chr19 | 35758185 | *LSR* | -0.13 | 1 |
| cg26703182 | chr19 | 51601884 | *CTU1* | -0.22 | 1 |
| cg11177693 | chr2 | 27604063 | *ZNF513* | -0.14 | 1 |
| cg20604286 | chr2 | 58273438 | *VRK2* | -0.18 | 1 |
| cg19638749 | chr2 | 70312615 |  | -0.10 | 1 |
| cg20415811 | chr2 | 74700246 | *CCDC142* | -0.11 | 1 |
| cg20520115 | chr2 | 75063838 | *HK2* | -0.10 | 1 |
| cg23725986 | chr2 | 75938438 | *C2orf3* | -0.20 | 1 |
| cg22809047 | chr2 | 101618261 | *RPL31* | -0.13 | 1 |
| cg20234855 | chr2 | 120980555 | *TMEM185B* | -0.10 | 1 |
| cg26799474 | chr2 | 202098951 | *CASP8* | -0.15 | 1 |
| cg13372293 | chr2 | 203878778 | *NBEAL1* | -0.16 | 1 |
| cg13903421 | chr2 | 219738714 | *WNT6* | 0.25 | 1 |
| cg03727500 | chr2 | 232348334 |  | -0.25 | 1 |
| cg08129953 | chr2 | 242448802 | *STK25* | -0.12 | 1 |
| cg07889201 | chr20 | 2490030 | *ZNF343* | -0.11 | 1 |
| cg13518079 | chr20 | 2675072 | *EBF4* | 0.36 | 1 |
| cg06726390 | chr20 | 2820927 | *VPS16* | -0.17 | 1 |
| cg07364906 | chr20 | 16710288 | *SNRPB2* | -0.14 | 1 |
| cg05779219 | chr20 | 23032166 |  | -0.13 | 1 |
| cg20318748 | chr20 | 25605178 | *NANP* | -0.18 | 1 |
| cg22329555 | chr20 | 30777558 | *TSPYL3* | -0.14 | 1 |
| cg23414001 | chr20 | 33681070 | *TRPC4AP* | -0.12 | 1 |
| cg12303084 | chr20 | 45985741 | *ZMYND8* | -0.13 | 1 |
| cg03264550 | chr20 | 57465448 | *GNAS* | -0.14 | 1 |
| cg15920739 | chr21 | 33765737 | *URB1* | -0.10 | 1 |
| cg10599571 | chr21 | 35445161 | *MRPS6* | -0.14 | 1 |
| cg18829411 | chr21 | 40722023 | *HMGN1* | -0.22 | 1 |
| cg03131358 | chr22 | 42195972 | *CCDC134* | -0.11 | 1 |
| cg22189786 | chr22 | 42395067 | *WBP2NL* | -0.25 | 1 |
| cg08057985 | chr22 | 42470123 | *FAM109B* | -0.14 | 1 |
| cg18034295 | chr22 | 42475135 | *C22orf32* | -0.12 | 1 |
| cg20744362 | chr22 | 50050164 | *C22orf34* | -0.35 | 1 |
| cg02776313 | chr22 | 50965782 | *TYMP* | -0.19 | 1 |
| cg25516803 | chr3 | 42307519 | *CCK* | 0.14 | 1 |
| cg05654765 | chr3 | 49170727 | *LAMB2* | -0.14 | 1 |
| cg22993195 | chr3 | 49756301 | *AMIGO3* | -0.29 | 1 |
| cg05546296 | chr3 | 50376006 | *RASSF1* | -0.22 | 1 |
| cg23431721 | chr3 | 50387780 | *TUSC4* | -0.13 | 1 |
| cg09439920 | chr3 | 99979117 | *TBC1D23* | -0.21 | 1 |
| cg16984944 | chr3 | 99979425 | *TBC1D23* | -0.12 | 1 |
| cg17228765 | chr3 | 112738800 | *C3orf17* | -0.14 | 1 |
| cg05725721 | chr3 | 160823387 | *B3GALNT1* | -0.11 | 1 |
| cg14553504 | chr3 | 169898657 | *PHC3* | -0.19 | 1 |
| cg09866366 | chr3 | 183903315 | *ABCF3* | -0.15 | 1 |
| cg26152983 | chr3 | 184428654 | *MAGEF1* | -0.15 | 1 |
| cg04134015 | chr3 | 193310532 | *OPA1* | -0.12 | 1 |
| cg03456393 | chr3 | 193310565 | *OPA1* | -0.16 | 1 |
| cg10432947 | chr4 | 2062441 | *NAT8L* | 0.22 | 1 |
| cg18089397 | chr4 | 2264747 | *MXD4* | -0.13 | 1 |
| cg08572214 | chr4 | 73936052 | *COX18* | -0.23 | 1 |
| cg18160135 | chr4 | 99851115 | *EIF4E* | -0.11 | 1 |
| cg08814800 | chr4 | 174421377 |  | 0.13 | 1 |
| cg10059484 | chr4 | 183839326 | *DCTD* | -0.10 | 1 |
| cg21490662 | chr4 | 185571520 | *CASP3* | -0.12 | 1 |
| cg17351974 | chr5 | 40835760 | *RPL37* | -0.19 | 1 |
| cg13028113 | chr5 | 60458778 | *C5orf43* | -0.15 | 1 |
| cg10441379 | chr5 | 72793693 | *BTF3* | -0.28 | 1 |
| cg17330048 | chr5 | 80257322 | *RASGRF2* | 0.13 | 1 |
| cg12532266 | chr5 | 132201652 | *GDF9* | -0.13 | 1 |
| cg12924095 | chr5 | 151150029 | *G3BP1* | -0.29 | 1 |
| cg16046214 | chr5 | 170289848 | *RANBP17* | -0.13 | 1 |
| cg27583138 | chr6 | 24774877 | *GMNN* | -0.27 | 1 |
| cg07816556 | chr6 | 26017280 | *HIST1H1A* | -0.27 | 1 |
| cg13836098 | chr6 | 26225268 | *HIST1H3E* | -0.20 | 1 |
| cg27490387 | chr6 | 26520793 | *HCG11* | -0.14 | 1 |
| cg10184431 | chr6 | 27783226 | *HIST1H2AJ* | -0.12 | 1 |
| cg00811535 | chr6 | 31587543 | *BAT2* | -0.12 | 1 |
| cg13142134 | chr6 | 42695332 |  | -0.19 | 1 |
| cg11742202 | chr6 | 74364667 | *SLC17A5* | -0.16 | 1 |
| cg04140496 | chr6 | 111279511 | *GTF3C6* | -0.11 | 1 |
| cg02359132 | chr6 | 111279585 | *GTF3C6* | -0.11 | 1 |
| cg05220968 | chr6 | 146057943 | *EPM2A* | -0.18 | 1 |
| cg15298323 | chr6 | 160182438 | *ACAT2* | -0.14 | 1 |
| cg09259772 | chr7 | 12726089 | *ARL4A* | -0.16 | 1 |
| cg09755872 | chr7 | 23245557 |  | -0.19 | 1 |
| cg05072008 | chr7 | 50518647 | *FIGNL1* | -0.21 | 1 |
| cg27441048 | chr7 | 72972316 | *BCL7B* | -0.15 | 1 |
| cg08558340 | chr7 | 100472263 | *SRRT* | -0.26 | 1 |
| cg08159594 | chr7 | 102790168 | *NAPEPLD* | -0.15 | 1 |
| cg08586737 | chr7 | 127225949 | *GCC1* | -0.15 | 1 |
| cg19183166 | chr7 | 150020108 | *LRRC61* | -0.15 | 1 |
| cg21134096 | chr7 | 151217883 | *RHEB* | -0.10 | 1 |
| cg24531520 | chr7 | 151217947 | *RHEB* | -0.10 | 1 |
| cg19657214 | chr8 | 6794872 | *DEFA4* | -0.14 | 1 |
| cg00186701 | chr8 | 98290510 | *TSPYL5* | -0.16 | 1 |
| cg02161761 | chr8 | 120868748 | *DSCC1* | -0.13 | 1 |
| cg07745674 | chr8 | 124171207 |  | 0.10 | 1 |
| cg10105237 | chr8 | 135726091 | *ZFAT* | -0.16 | 1 |
| cg26039042 | chr8 | 144815275 | *FAM83H* | -0.13 | 1 |
| cg26440467 | chr8 | 145654605 | *VPS28* | -0.16 | 1 |
| cg20394284 | chr9 | 4984206 | *JAK2* | -0.14 | 1 |
| cg11304734 | chr9 | 37485592 | *POLR1E* | -0.10 | 1 |
| cg21171204 | chr9 | 89766723 | *C9orf170* | -0.12 | 1 |
| cg18521771 | chr9 | 117373239 | *C9orf91* | -0.11 | 1 |
| cg13655082 | chr9 | 125109046 |  | -0.19 | 1 |
| cg14560040 | chr9 | 136203305 | *SURF6* | -0.11 | 1 |
| cg14495033 | chr9 | 140707378 | *EHMT1* | 0.14 | 1 |

Figure S1 – Volcano plot of the methylation analysis


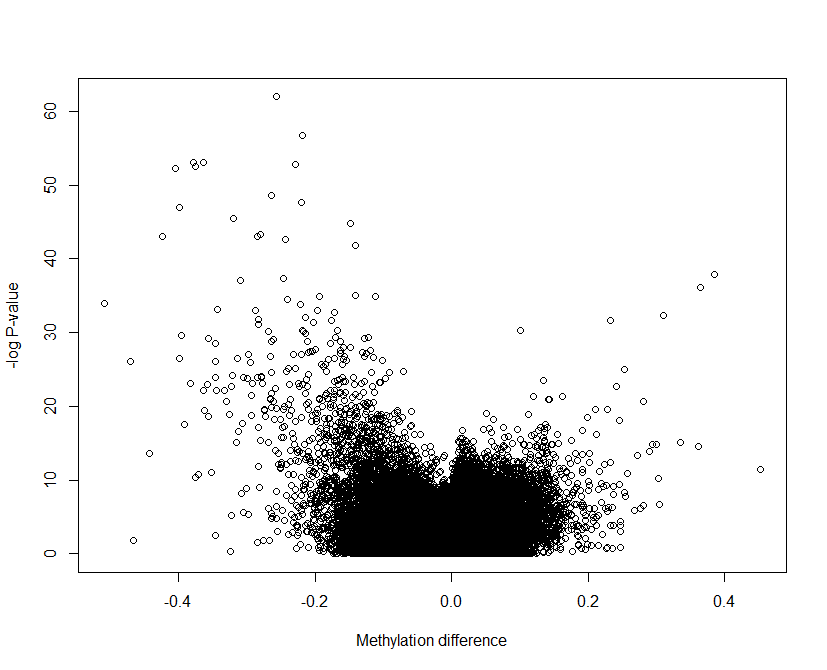

Supplement: Supplementary file 1 — Table S1. CpG probes differentially methylated between the affected males and controls (n = 1,769). Table S2. The most differentiating and non-redundant probes selected from the epi-signature of Claes-Jensen syndrome (n = 198). Figure S1. Volcano plot of the methylation analysis. (DOCX 1865 kb) [file 13148_2018_453_MOESM1_ESM.docx]
